# Data-driven mechanisms for network freight platforms: An evolutionary game perspective

**DOI:** 10.1371/journal.pone.0319842

**Published:** 2025-06-27

**Authors:** Chan He, Qun Xu, Xu Xu, Feng Kong, Feng Shen

**Affiliations:** 1 Business School, Shanghai Dianji University, Pudong, Shanghai, China; 2 Xiangfu Laboratory, Jiashan, Zhejiang, China; Shanghai Jiao Tong University, CHINA

## Abstract

This paper examines the impact of data-driven mechanisms in network freight platforms. The main objective is to understand how these mechanisms can improve operational efficiency and encourage cooperation among key stakeholders, including a risk-neutral shipper, a loss-averse carrier and network freight platform. The study uses evolutionary game theory to model the interactions between these parties. Numerical simulations are conducted to evaluate the effects of initial conditions and important parameters on cooperation. The results show that consistent implementation of data-driven mechanisms fosters stable and honest cooperation. Key factors, such as subsidies, penalties for dishonest behavior, the likelihood of detecting dishonesty, and incentives for honest actions, motivate carriers and shippers to participate in fair transactions. Additionally, specific costs are identified as deterrents to dishonest practices. These findings contribute to our understanding of digital transformation and provide valuable insights for enhancing resilience and collaboration within network freight platforms. The risk appetite can obviously influence the decision of the three parties. The study also highlights important implications for policymakers and industry practitioners, emphasizing the importance of effective data governance and the strategic use of information to shape the future of freight logistics.

## Introduction

The Network freight platforms (NFP) have transformed traditional supply chains by connecting shippers and carriers through digital platforms, enabling more efficient freight matching and resource optimization [1–6]. They leverage technology to streamline the logistics process, optimize route planning and freight operations, enable seamless transactions, and enhance visibility and transparency [5–8]. By leveraging data analytics, machine learning, and advanced marketing techniques, NFPs have the potential to open fresh avenues for expansion, breakthroughs in innovation, and the generation of value within the freight transportation sector [6,8–12].

Many scholars have paid attention to and studied the NFP and its dynamic role, challenges and opportunities in supply chain [9–16]. Some scholars examined the effects of NFP on efficiency, collaboration, and value creation, as well as the obstacles faced in supply chain management [10,11,17–19]. Rodrigue [7] underscored the contribution of NFPs to the advancement of supply chain clarity, openness, and agility. Researchers are increasingly aware of the importance of data-driven freight platforms [20–24]. Evolutionary game model provides a powerful framework for the dynamic interplay of incentives, preferences, and strategies by stakeholders as they seek to optimize utility and achieve competitive advantage [21–26]. NFPs represent a sophisticated infrastructure wherein multiple stakeholders interact and transact to facilitate the movement of goods across diverse geographic locations. Some scholars have discussed this problem from the perspective of evolutionary game [27–30]. However, from the perspective of data-driven mechanism, there are few topics to discuss whether the NFP adopts positive data-driven mechanisms (DDM) to the shipper and carrier.

The integration of data-driven mechanisms (DDMs) in network freight platforms (NFPs) addresses pressing challenges in modern logistics, such as reducing inefficiencies, fostering collaboration, and improving decision-making. Existing studies highlight the transformative potential of DDMs, including their ability to enhance real-time visibility, optimize freight allocation, and mitigate information asymmetry among stakeholders [1,3,7]. For instance, blockchain-enabled platforms have been shown to increase trust between shippers and carriers by ensuring transaction transparency, while predictive analytics improve route optimization and demand forecasting [8,9]. These advancements have demonstrated measurable improvements in supply chain efficiency, including cost savings, reduced lead times, and increased resource utilization [10–12].

Despite these advancements, there is limited research exploring how DDMs influence strategic behaviors and interactions among stakeholders in the NFP ecosystem. Most existing contributions focus on technical capabilities and operational outcomes, with insufficient attention given to the mechanisms by which DDMs foster trust, cooperation, and long-term engagement. By addressing this gap, the present study provides valuable insights into how DDMs can drive collaboration and elevate supply chain performance through improved stakeholder dynamics.

This study investigates how data-driven mechanisms can improve cooperation between stakeholders in network freight platforms (NFPs), resulting in more stable and efficient outcomes. The research utilizes evolutionary game theory (EGT) to examine the interactions and strategic behaviors among NFPs, shippers, and carriers and identify the impact of key factors on decision-making in complex systems. EGT is well-suited for this analysis because it models how stakeholders adapt their strategies over time, responding to incentives and penalties that either promote or inhibit cooperation. This dynamic approach is crucial for understanding the evolving nature of stakeholder interactions in the context of NFPs, where incentives and penalties can significantly impact decision-making and behavior. The paper aims to advance the innovation of traditional NFPs and foster the development of efficient and sustainable supply chain management.

To achieve these objectives, numerical simulations are conducted to analyze how different initial conditions and key parameters influence the decision-making of shippers, carriers, and platform operators. The key findings show that strategic interventions—such as offering subsidies or imposing penalties—can significantly influence stakeholder behavior, leading to more cooperative and stable outcomes. These findings contribute to the theoretical understanding of NFP operations and provide practical recommendations to enhance cooperation, resilience, and competitiveness in digital logistics systems.

Through mathematical modeling and numerical simulations, the study demonstrates that consistent implementation of data-driven mechanisms promotes stable and honest cooperation among stakeholders. However, while the model assumes that all participants make purely rational decisions based on financial incentives and penalties, we acknowledge that real-world decision-making often involves cognitive biases, emotions, and social factors that are not captured in traditional economic models. This limitation reduces the model’s ability to fully represent the complexities of human behavior in networked supply chains, where stakeholders might deviate from purely rational economic calculations.

This study builds upon a rich body of literature on network freight platforms (NFPs) and data-driven mechanisms (DDMs), offering novel contributions in three distinct areas.

1) Integration of DDMs in Evolutionary Game Models. While prior research has extensively examined the role of government policies and static strategies in logistics management [1,7,10], this paper uniquely focuses on the dynamic influence of DDMs within NFPs. By incorporating parameters such as subsidies, penalties, and bonuses, adjusted based on evolving market conditions and stakeholder behaviors, the study bridges a critical gap in understanding how DDMs actively shape the strategic interactions among NFP stakeholders.
2) Comprehensive Analysis of DDM Impacts. Previous studies have primarily investigated the technical aspects of DDMs, such as data analytics and real-time monitoring [8,14,19]. In contrast, this paper delves into their strategic dimensions, evaluating the effects of incentives and costs on carriers’ and shippers’ behaviors. The loss aversion preference of members is also considered. The study highlights how DDMs can mitigate dishonest practices and foster cooperative behavior, contributing to a more ethical and efficient logistics ecosystem.
3) Stability Analysis of Strategy Equilibria. Unlike prior works that emphasize operational outcomes [15,21,25], this research adopts an evolutionary game perspective to analyze the stability of equilibrium points under various conditions. By identifying evolutionarily stable strategy combinations, the paper provides actionable insights for designing robust mechanisms that enhance cooperation and trust within NFPs.

## Theoretical development

The integration of data-driven mechanisms within the domain of network freight platforms (NFPs) has garnered significant attention from both academic researchers and industry practitioners. The objective of this literature synthesis is to present a comprehensive summary of pivotal research, theoretical frameworks, and applied practices concerning data-centric methods within NFP supply chains, with a specific focus on the lens of EGM.

This literature review aims to provide an overview of key studies, theories, and practical applications related to data-driven approaches in NFP supply chains, particularly from an evolutionary game theory perspective. The literature review is organized into three main parts. The first part provides a foundational understanding of Network Freight Platforms (NFPs), including their structure, operational mechanisms, and technological underpinnings. The second part highlights recent advancements in NFPs, focusing on their integration with data-driven mechanisms (DDMs) and the resulting benefits and challenges. The final part delves into the theoretical framework of EGT. It highlights how EGT provides a robust model for analyzing adaptive strategies among NFP stakeholders under dynamic conditions.

### Network freight platforms

Network freight platforms (NFPs) is as a vital part of modern logistics systems, providing a digital space where shippers and carriers can converge for optimized freight pairing and route optimization [2–6]. According to Christopher [1], NFPs utilize Internet of Things (IoT), artificial intelligence (AI), and blockchain technologies to enable real-time shipment visibility, tracking, and coordination. These innovations not only improve transparency and reliability in the supply chain but also offer substantial cost savings and operational efficiencies [3–5].

Rodrigue [7] emphasizes the critical role of NFPs in fostering supply chain visibility, transparency, and flexibility. By providing a centralized platform for transactional activities, NFPs reduce transaction costs, mitigate inefficiencies associated with traditional freight brokerage methods, and improve decision-making through advanced data analytics. Additionally, NFPs support dynamic resource allocation, allowing carriers to optimize fleet utilization and shippers to access on-demand transportation services [8]. Beyond these operational benefits, NFPs are recognized for their potential to drive market innovation. Herold [9] highlighted the disruption caused by digital freight platforms in traditional logistics markets, transforming conventional business models by automating processes and creating more competitive pricing structures. By aggregating vast amounts of data, NFPs facilitate predictive analytics and real-time decision-making, which can adapt to shifting market conditions and evolving customer demands.

Despite these advancements, NFPs face several challenges that limit their scalability and effectiveness. One key challenge is the reliance on critical mass to achieve network effects. The success of an NFP often depends on its ability to attract enough shippers and carriers, creating a self-sustaining ecosystem of transactions [11,18]. Furthermore, issues related to data security, interoperability, and regulatory compliance present significant hurdles. For instance, ensuring the protection of sensitive logistics information while maintaining operational transparency requires robust cybersecurity measures and regulatory frameworks [10,16].

The role of trust among stakeholders also remains a critical barrier. Information asymmetry between shippers and carriers often leads to mistrust and opportunistic behavior, such as misrepresentation of shipment conditions or renegotiation of agreed terms [15,20]. To address these challenges, NFPs have increasingly turned to data-driven mechanisms (DDMs), integrating advanced algorithms and real-time monitoring systems to foster cooperation and accountability among stakeholders. These mechanisms not only enhance operational efficiency but also establish a foundation for ethical collaboration by penalizing dishonest behaviors and rewarding integrity [21,24].

NFPs continue to evolve as key players in the logistics sector, driving digital transformation and innovation. However, to fully realize their potential, future research must focus on addressing the scalability, trust, and regulatory challenges faced by these platforms. Moreover, the integration of data-driven mechanisms and advanced technologies will remain critical in shaping their role in achieving sustainable and resilient supply chains.

### Data-driven mechanism in NFPs

Despite the advantages, NFPs encounter challenges, particularly in fostering cooperation and trust among ship-pers, carriers, and platforms. He [10] highlighted issues such as data security concerns, interoperability challenges, and regulatory complexities as barriers to the widespread adoption of NFPs. Moreover, the scalability and sustainability of NFPs depend on the ability to achieve critical mass and network effects, requiring collaboration among multiple stakeholders and an alignment of incentives [11]. Strategic behaviors among stakeholders in NFPs are influenced by various incentives, including pricing strategies, capacity utilization, and information asymmetries [13–17]. Chen [16] pointed out that for NFPs stewards, leveraging information-oriented incentives can be a more potent method for activating logistics information providers and for refining logistics information accuracy when contrasted with traditional incentives; they are also tasked with instituting a regulatory system with punitive aspects as part of the informatization process. Almotairi proposed that the port’s function within supply chains can be understood as a key element when analyzed through the soft systems perspective [18]. Additionally, investments in infrastructure, digital literacy, and talent development are essential to support the growth and resilience of NFPs in an increasingly digitized and interconnected world [20].

The integration of algorithms and predictive analytics enhances the capabilities of NFPs, allowing stakeholders to predict demand oscillations, to refine inventory policies, and to hedge against supply chain uncertainties [21]. Policy-makers are key contributors to the development of a regulatory ecosystem that fosters and supports innovative practices in NFP supply chain processes. Wu discussed policy recommendations for promoting the adoption of data-driven mechanisms in logistics, including investments in infrastructure, standardization of data formats, and support for research and development initiatives [19]. The evolution of NFPs is closely intertwined with technological innovations that drive digital transformation in logistics. According to Wang [22], DDM supports instant access to supply chain insights, forward-looking analytics, and the improvement of operational effectiveness. The integration of data-driven mechanisms within NFPs has transformative implications for supply chain management. Li [23] highlighted how data analytics can enable live monitoring of cargo, optimize delivery routes, and identification of potential disruptions. By harnessing data-driven mechanism, NFPs have the potential to boost visibility, consistency, and responsiveness within supply chain activities. Deng [24] emphasizes the importance of IoT devices in providing continuous tracking and monitoring solutions for shipments, ensuring visibility and accountability throughout the supply chain. The existing literature focuses on technical capabilities of data-driven mechanism but lacks a focus on their impact on strategic stakeholder behavior and how these mechanisms promote honest interactions. To fill this gap, this study analyzes how data-driven mechanism enhances trust and cooperative behaviors in NFPs.

### Evolutionary game theory in Networked Systems

Evolutionary game theory offers a powerful analytical framework for studying the adaptive strategies of participants within dynamic and interconnected systems, such as supply chains and network freight platforms (NFPs) [25]. EGT’s applicability in complex networked environments is well-established, as it models how strategic interactions evolve over time, considering both competitive and cooperative behaviors.

While EGT has long been used to explore cooperation and competition in various domains—such as pricing dynamics, inventory management, and organizational collaboration—recent advancements extend its application to more complex and dynamic systems. Perc [26], for example, demonstrated that EGT is particularly useful for capturing the evolution of cooperation and competition in complex systems, where players may adjust their strategies based on real-time information. One of the central themes in EGT is the balance between cooperation and competition among participants [27]. Vasile [28] utilizes the various factors that shape the rewards of specific strategies that companies can adopt and identifies the requirements for cooperation or competition while simultaneously developing scenarios and forecasts for the evolution of these phenomena. Li [29] explored the role of regulatory measures in driving green innovation among firms, revealing how governments use systemic regulation and subsidies to influence cooperation. Similarly, Du [30] stated that the EGM reveals the movement and determinants of the cross-network influence, thereby shaping the overall system dynamics. These studies underscore the robustness of EGT in capturing the strategic interactions and feedback loops that drive networked systems.

While much of the existing research applies EGT to static or deterministic models of interaction, there is a gap in understanding how dynamic and probabilistic factors—such as real-time feedback and data-driven incentives—affect stakeholder behavior. Most studies focus on rational decision-making based on cost-benefit analyses and fixed strategies. However, real-world interactions often involve uncertainty, asymmetry, and bounded rationality, where stakeholders must adapt to evolving market conditions, regulatory changes, and stakeholder behaviors. Recent research in behavioral economics highlights how psychological factors, such as trust, risk aversion, and loss aversion, can significantly influence decision-making in collaborative environments [31,32]. These insights suggest that EGT can be further enriched by incorporating real-time, dynamic parameters, which is particularly relevant in the context of NFPs, where digital tools such as data-driven mechanisms (DDMs) continuously adjust incentives based on evolving conditions.

In this study, we extend EGT by applying it to the dynamic interactions in NFPs, incorporating DDMs that influence stakeholder strategies in real time. Unlike previous studies, which have primarily used static models, this paper integrates the probabilistic nature of stakeholder behavior and feedback loops. The model accounts for varying detection probabilities, penalties, rewards, and market conditions, providing a nuanced understanding of how DDMs influence the evolution of strategies and cooperation in NFP ecosystems. This approach contributes to the broader body of EGT applications by extending its principles to digital logistics platforms, with implications not only for NFPs but also for other networked systems characterized by dynamic interactions, information asymmetry, and adaptive behaviors.

## Problem description and modeling assumptions

### Problem description

The NFP supply chain comprises three key participants: shippers, carriers, and the NFP itself. The behavior of participants in NFPs is driven by several critical factors: incentives, penalties, market conditions, and the need to optimize operational efficiency. The interactions between these stakeholders form a complex, adaptive system where cooperation and competition coexist, requiring careful management of incentives to ensure the efficient functioning of the platform.

Shippers entrust goods, pay for transportation and aim to minimize transportation costs while ensuring reliable and timely deliveries. Their decisions often depend on the available carriers, freight options, and the platform’s service offerings. Shippers’ decision-making is influenced by factors such as cost, carrier reputation, and service quality. Research has highlighted the role of price sensitivity and the importance of reputation in shaping shipper behavior in logistics networks [22–24,33]. Moreover, data-driven mechanisms, such as real-time tracking and performance metrics, can increase transparency, thereby allowing shippers to make more informed decisions [18–21].

Carriers transport goods under NFP authorization and seek to optimize their fleet utilization, maximize profits, and reduce operational costs. Their strategies are typically based on market demand, pricing mechanisms, and the availability of cargo. The dynamics between carriers and shippers are often shaped by price negotiations, load balancing, and capacity management. Previous studies show that carriers’ behavior is influenced by economic factors such as fuel prices, demand fluctuations, and fleet management constraints [10]. Furthermore, the introduction of dynamic data-driven incentives, such as bonuses for on-time deliveries and penalties for delays, can promote greater cooperation among carriers, leading to improved service levels and optimized resource usage [3–8,19–21,34].

NFP operators are responsible for managing the platform, ensuring that incentives, penalties, and regulatory mechanisms align with the platform’s strategic goals. They act as intermediaries between shippers and carriers, setting rules that govern cooperation and competition. The role of platform operators is crucial for maintaining a balance between these two forces, ensuring that shippers and carriers have sufficient incentives to cooperate while competing for the best deals. The NFP’s role extends to supervising the quality of services, ensuring the integrity of all parties, and managing risks through various measures such as carrier qualification control, deposit collection, and insurance procurement. The platform also implements performance-based rewards and penalties for users. Previous research has emphasized the importance of operator-driven mechanisms, such as information sharing, capacity allocation, and real-time performance tracking, which help reduce inefficiencies and promote collaboration [19–22].

The relationships between shippers, carriers, and NFP operators are both cooperative and competitive. Shippers and carriers must collaborate to ensure the efficient flow of goods, but they also engage in competitive behavior to secure favorable terms. This dynamic is often referred to as ‘coopetition’—a term used to describe simultaneous cooperation and competition between actors within the same system [34]. In the context of NFPs, this dynamic is further complicated by information asymmetry, where different stakeholders may have access to different levels of information regarding market conditions, shipment status, and performance metrics. Data-driven mechanisms, such as real-time tracking systems, predictive analytics, and machine learning-based decision support, have become integral in modern NFPs. DDMs enhance transparency by providing all stakeholders with access to critical data that can inform their decisions, helping to reduce information asymmetry and promote fair competition. For instance, the use of blockchain for secure and transparent transactions ensures that shippers and carriers can trust the platform, thereby fostering cooperation [3,8,10,16].

By integrating these insights, this study models how DDMs influence the strategic behavior of NFP participants, focusing on their ability to alter decision-making processes, enhance cooperation, and optimize platform performance. This dynamic approach is essential for understanding how the integration of data-driven tools can promote sustainable and resilient logistics ecosystems. To address the limited realism of purely rational assumptions, the bounded rationality hypothesis that considers loss aversion is included in this model.

**Fig 1:**
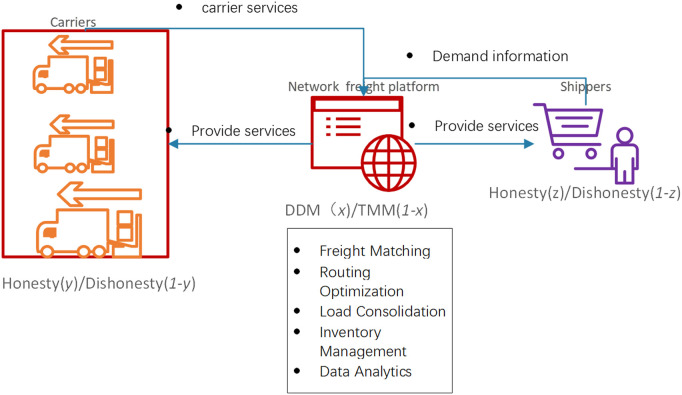
Relationship among the participants in network freight platform supply chain. (DDM stands for digital-driven mechanisms and TMM stands for traditional means marketing).

The relationship among the participants in the NFP supply chain is illustrated in Fig 1.

Fig 1 illustrates the interactions and relationships among the three key stakeholders in the NFP supply chain: shippers, carriers, and the NFP itself. It shows how information and resources flow among these entities and highlights the role of the NFP as a digital intermediary that facilitates efficient freight matching and coordination.

Data-driven mechanisms offer significant advantages for NFPs, but they require investments in technology and analysis. Maintaining honesty among carriers is vital for NFP success. Honest carriers fulfill their obligations and complete tasks reliably, while dishonest carriers can cause delays, break contracts, deliver incorrect or lost goods, provide inaccurate documentation, and complicate customs clearance. These actions lead to dissatisfied customers, damage to the reputations of carriers and platforms, and potential legal issues. Shippers depend on platforms for efficient transportation, and any issues erode trust. NFPs must monitor carriers, enforce rewards and penalties, and assist shippers in resolving problems.

Honesty for shippers involves integrity, transparency, compliance, and responsible behavior. Dishonest shippers may provide false information, misrepresent goods, or forge documents. They may withhold information about cargo nature, hazards, or special handling requirements, causing complications. Shippers might under declare cargo values to avoid taxes or fees, leading to legal consequences and losses. Misrepresenting cargo condition can result in disputes and financial losses. Robust verification processes, documentation checks, and collaboration between carriers, NFPs, and authorities are essential to detect and prevent dishonesty. Data-driven techniques facilitate the identification and resolution of dishonest practices.

To ensure practical relevance, we relate the model’s parameters to real-world NFP operations. For instance, penalties imposed on dishonest carriers align with the practices of leading platforms like Uber Freight, where delays or misreported freight conditions incur financial fines ranging from $100 to $500. Similarly, subsidies for shippers and carriers, such as bonuses for on-time deliveries or accurate documentation, are common incentives observed in platforms like Maersk, where performance-based rewards improve compliance. Detection probabilities, represented in this study, reflect the growing use of technologies like AI-powered monitoring and blockchain systems, which increase the likelihood of detecting dishonest behavior to over 90% in modern digital logistics environments.

### Modeling assumptions

The model is based on bounded rationality and considers costs, penalties, and benefits associated with each participant’s strategies. In this study, several key modeling assumptions are made to capture the dynamics of stakeholders in network freight platforms (NFPs). These assumptions, while necessary for simplifying the complex interactions between shippers, carriers, and platform operators, are grounded in previous literature and established research.

#### Assumption 1. Assumption of immediate feedback from Data-Driven Mechanisms (DDMs).

The model assumes that stakeholders receive real-time feedback from the platform’s data-driven mechanisms, which adjust incentives based on performance. This assumption is supported by studies on the impact of real-time data and performance-based incentives in logistics platforms [3,8,9,14,16,19,33]. Although the impact of real-time feedback might vary depending on the technological infrastructure, the assumption is reasonable given the increasing prevalence of data-driven systems in modern supply chain.

#### Assumption 2. Assumption of loss preference and bounded rationality.

Loss aversion refers to the psychological bias where losses are perceived as more impactful than equivalent gains. This leads to more conservative or defensive strategies [33,35]. Carriers are normally multinational corporations, meaning that they have various measures to counter loss, whereas shippers are focused on one specific industry and are less able to diversify risk. Therefore, in this paper, we take the loss-aversion of the shipper into account [36], the coefficient of loss-aversion is $L_{S}$. The NFP is generally considered to be weak loss-aversion [33,34,37], with the coefffcient of loss-aversion is $L_{P}$. In the game model, loss aversion is usually reflected by adjusting the utility function or the payment function to reflect the player’s sensitivity to loss [33,35,37,38]. Bounded rationality is increasingly recognized as a critical factor in stakeholder decision-making, where individuals often act based on limited information or heuristic reasoning. To better simulate real-world decision-making processes, this paper assumes that all three parties are bounded rationality

#### Assumption 3. Strategy choices and probabilities.

The NFP typically faces two strategic options: adopting digital-driven mechanisms (DDM) or relying on traditional means marketing (TMM). However, the probability of adopting either strategy is not fixed; instead, it evolves dynamically over time as the NFP responds to market conditions, competitive pressures, and the observed behavior of stakeholders. This dynamic probability, denoted as *x* for DDM and 1−x for TMM, reflects the NFP’s mixed strategy. The carrier’s strategy set includes being “Honest” with probability y or “Dishonest” with probability 1−y. The shipper’s strategy set is similar, with honesty probability z and dishonesty probability 1 − z.

#### Assumption 4. NFP earnings and costs.

The NFP gains basic earnings $\psi_{1\;}$ from its operation. If the NFP adopts DDM, it incurs additional costs $C_{1}$ but gains extra revenue $R_{1}$ and corporate reputation benefits W. Honest behavior from carriers and shippers earns them subsidies $S_{1}$ and $S_{2}$, while dishonesty results in penalties $T_{1}$ and $T_{2}$.

#### Assumption 5. Carrier and shipper earnings.

The carrier access platform, with a base income of $\psi_{2}$, incurs a higher cost $C_{2}$ under an honest strategy than a dishonest one. The shipper access platform, with a base income of $\psi_{3}$, also experiences a higher cost $C_{3}$ with honesty versus dishonesty. Dishonest strategies by carriers and shippers yield additional revenues $G_{1}$ and $G_{2}$, while the NFP provides compensation for third-party fraud $H_{1}$ and $H_{2}$ for the carrier and shipper, respectively.

#### Assumption 6. Detection of dishonesty.

Assume the probability of the carrier’s or shipper’s dishonest strategy being detected by the NFP using DDM is α, with penalties denoted as $\alpha T_{1}$ and $\alpha T_{2}$, and subsidies as $(1-\alpha )S_{1}$ and ${(1-\alpha )S}_{2}$, and the NFP’s compensations for such behavior are ${\alpha H}_{1}$ and $\alpha H_{2}$ for the carrier and shipper, respectively. Similarly, under TMM, the probability of detection is β, with penalties $\beta T_{1}$ and $\beta T_{2}$, subsidies $(1-\beta )S_{1}$ and ${(1-\beta )S}_{2}$, and compensations ${\beta H}_{1}$ and $\beta H_{2}$ for the carrier and shipper, respectively.

#### Assumption 7.

Suppose the shipper suffers a loss $K_{1}$ if the carrier is dishonest, and the carrier suffers a loss $K_{2}$ from dishonest shippers. The additional benefits under DDM for the NFP, carrier, and shipper are $R_{1},\;R_{2}$ and $R_{3}$, respectively.

By grounding these modeling assumptions in existing literature, this study ensures that the proposed framework is not arbitrary but instead builds on well-established theories and findings.

Table 1 presents the definitions and explanations of the principal parameters and variables utilized in this study.

**Table 1:** Parameters and meaning.

Parameters	Meaning
x	The probability of the NFP choosing the “DDM” strategy, and 0≤x≤1.
y	The probability of the carrier choosing the “Honest” strategy, and 0≤y≤1.
z	The probability of the shipper choosing the “Honest” strategy, and 0≤z≤1.
$\psi_{1}$	The basic earnings from the operation of the NFP;
$\psi_{2}/\psi_{3}$	The underlying income of the carrier(shipper) access platform;
$R_{i},i=1,2,3$	Additional benefits for the NFP/ carrier/shipper under DDM system conditions;
α	The probability of the carrier and shipper’s dishonest strategy discovered by the NFP under DDM;
β	The probability of the carrier and shipper’s dishonest strategy discovered by the NFP under TMM;
$C_{1}$	The extra cost for DDM spent by the NFP;
$C_{2}/C_{3}$	The extra cost for honest strategy spent by the carrier/shipper;
$S_{1}/S_{2}$	Subsidy given by the NFP to the carrier/shipper with honest strategy, respectively;
$T_{1}/T_{2}$	The penalty to the carrier/ shipper with dishonest strategy;
$H_{1}/H_{2}$	The remediation from the NFP to the carrier/ shipper under the dishonest behavior of the others;
$G_{1}/G_{2}$	The additional revenue obtained by the carrier/ shipper from dishonest strategy;
W	Corporate reputation benefits for adoption of DDM;
$K_{1}$	A loss the shipper suffered if the carrier is dishonest;
$K_{2}$	A loss the shipper suffered if the shipper is dishonest;
$L_{P}$	Coefffcient of loss-aversion of the NFP;
$L_{S}$	Coefffcient of loss-aversion of the shipper.

## Evolutionary game model and equilibrium analysis

### Income matrix in EGM

The income matrix for the NFP, carrier, and shipper under different strategies is summarized in Table 2. Each cell in the matrix represents the income when the NFP adopts DDM (x) or traditional methods (1−x), and when the carrier and shipper choose honest (y,z) or dishonest (1−y, 1−z) strategies.

**Table 2:** Income matrix for the NFP, carrier, and shipper under various strategies.

The NFP	The Carrier	The shipper	
		Honest (z)	Dishonest (1−z)
**DDM** (x)	**Honest** (y)	${\psi_{1}+R_{1}+W{-L_{P}(C}_{1}+S}_{1}+S_{2})$	$\psi_{1}+R_{1}+W-L_{P}C_{1}-L_{P}S_{1}-{(1-\alpha )L_{P}S}_{2}+{\alpha T}_{2}-\alpha L_{P}H_{2}$
		$\psi_{2}+R_{2}+S_{1}-C_{2}$	$\psi_{2}+R_{2}+S_{1}-C_{2}-K_{2}+\alpha H_{2}$
		$\psi_{3}+R_{3}+S_{2}-L_{S}C_{3}$	$\psi_{3}+R_{3}+G_{2}{+(1-\alpha )S}_{2}-\alpha L_{S}T_{2}$
	Dishonest (1−y)	$\psi_{1}+R_{1}+W{-L_{P}C}_{1}-(1-\alpha )L_{P}S_{1}-L_{P}S_{2}+{\alpha T}_{1}-{\alpha L_{P}H}_{1}$	$\psi_{1}+R_{1}+W-L_{P}C_{1}-(1-\alpha )L_{P}S_{1}-{(1-\alpha )L_{P}S}_{2}+\alpha T_{1}+\alpha T_{2}-\alpha {L_{P}H}_{1}-\alpha {L_{P}H}_{2}$
		$\psi_{2}+R_{2}+{(1-\alpha )S}_{1}+G_{1}-\alpha T_{1}$	$\psi_{2}+R_{2}+G_{1}-\alpha T_{1}-K_{2}+\alpha H_{2}$
		$\psi_{3}+R_{3}+S_{2}-L_{S}C_{3}-L_{S}K_{1}+\alpha H_{1}$	$\psi_{3}+R_{3}+G_{2}-\alpha L_{S}T_{2}-L_{S}K_{1}+\alpha H_{1}$
**TMM(1 −** x)	**Honest** (y)	$\psi_{1}-L_{P}S_{1}-{L_{P}S}_{2}$	$\psi_{1}-L_{P}S_{1}-{(1-\beta )L_{P}S}_{2}+\beta T_{2}-\beta {L_{P}H}_{2}$
		$\psi_{2}+S_{1}-C_{2}$	$\psi_{2}+S_{1}-C_{2}-K_{2}+{\beta H}_{2}$
		$\psi_{3}+S_{2}-L_{S}C_{3}$	$\psi_{3}+G_{2}+{(1-\beta )S}_{2}-{\beta L_{S}T}_{2}$
	**Dishonest** (1−y)	$\psi_{1}+\beta T_{1}-(1-\beta )L_{P}S_{1}-{L_{P}S}_{2}-\beta L_{P}H_{1}$	$\psi_{1}-(1-\beta )L_{P}S_{1}-{(1-\beta )L_{P}S}_{2}+\beta T_{1}+\beta T_{2}-\beta L_{P}H_{1}-\beta {L_{P}H}_{2}$
		$\psi_{2}+G_{1}-\beta T_{1}+(1-\beta )S_{1}$	$\psi_{2}+G_{1}+(1-\beta )S_{1}-\beta T_{1}-K_{2}+\beta H_{2}$
		$\psi_{3}+S_{2}-{L_{S}C}_{3}-L_{S}K_{1}+\beta H_{1}$	$\psi_{3}+G_{2}-L_{S}K_{1}-\beta {L_{S}T}_{2}+\beta H_{1}$

### Analysis of tripartite game equilibrium

This subsection will analyze the evolutionary equilibrium strategies within the tripartite game involving the NFP, carrier and shipper, which includes the constructions of their replication dynamic equations and the analysis of equilibrium evolutionary stability strategy based on the constructed replication dynamic equations. The replication dynamic for NFP is given as $F_{NFP}(x)=\frac{dx}{dt}$, where x denotes the probability that the NFP adopts DDM. Similarly, the replication dynamics for the carrier and shipper are $F_{C}(y)=\frac{dy}{dt}$ and $F_{S}(z)=\frac{dz}{dt}$, respectively.

#### Replication dynamic equation for NFP.

If the NFP adopts DDM, the numerical equation for its anticipated return can be expressed as follows:


$$\begin{array}{cc} E_{11}= & yz\left({\psi_{1}+R_{1}+W{-L_{P}C}_{1}-L_{P}S}_{1}-L_{P}S_{2}\right)+y(1-z)\left(\psi_{1}+R+W-L_{P}C_{1}-L_{P}S_{1}-{(1-\alpha )L_{P}S}_{2}+{\alpha T}_{2}-\alpha {L_{P}H}_{2}\right)\\ & +(1-y)z\left(\psi_{1}+R_{1}+W{-L_{P}C}_{1}-(1-\alpha ){L_{P}S}_{1}-{L_{P}S}_{2}+{\alpha T}_{1}-{\alpha L_{P}H}_{1}\right)\\ & +(1-y)(1-z)\left(\psi_{1}+R_{1}+W-L_{P}C_{1}-(1-\alpha ){L_{P}S}_{1}-{(1-\alpha )L_{P}S}_{2}+\alpha T_{1}+\alpha T_{2}-\alpha {L_{P}H}_{1}-\alpha L_{P}H_{2}\right) \end{array}$$ (1)

When the NFP doesn’t choose DDM, the expected return is:


$$\begin{array}{cc} E_{12}= & \;yz\left(\psi_{1}-{L_{P}S}_{1}-L_{P}S_{2}\right)+y(1-z)\left(\psi_{1}-{L_{P}S}_{1}-{(1-\beta )L_{P}S}_{2}+\beta T_{2}-\beta L_{P}H_{2}\right)\\ & +(1-y)z\left(\psi_{1}+\beta T_{1}-(1-\beta ){L_{P}S}_{1}-{L_{P}S}_{2}-\beta {L_{P}H}_{1}\right)\\ & +(1-y)(1-z)\left(\psi_{1}-(1-\beta ){L_{P}S}_{1}-{(1-\beta )L_{P}S}_{2}+\beta T_{1}+\beta T_{2}-\beta L_{P}H_{1}-\beta L_{P}H_{2}\right) \end{array}$$ (2)

The anticipated earning of the NFP is:


$$E_{1}=E_{11}x+E_{12}(1-x)$$ (3)

Thus, the replication dynamic equation under the NFP’ DDM tactics is:


$$\begin{array}{cc} F_{NFP}(x) & =\frac{dx}{dt}=x(1-x)(E_{11}-E_{12})=x\left(E_{11}-E_{1}\right)\\ & =\left[R_{1}+W-L_{P}C_{1}+{(\alpha -\beta )({L_{P}S}_{1}+L_{P}S_{2}-L_{P}H}_{1}-L_{P}H_{2}+T_{1}+T_{2}+{L_{P}H}_{1}y+L_{P}H_{2}z-L_{P}S_{1}y-L_{P}S_{2}z-T_{1}y-T_{2}z)\right]\\ & x(1-x) \end{array}$$ (4)

Equation (4) is derived from the principles of evolutionary game theory, where the rate of change in the adoption of a strategy depends on the product of its current frequency, the complement frequency, and the payoff difference between strategies.

#### Replication dynamic equation for carrier.

If the carrier chooses to be honest, the expected return is:


$$\begin{array}{cc} E_{21}= & \;xz\left(\psi_{2}+R_{2}+S_{1}-C_{2}\right)+x(1-z)(\psi_{2}+R_{2}+S_{1}-C_{2}-K_{2}+\alpha H_{2})+(1-x)z\left(\psi_{2}+S_{1}-C_{2}\right)\\ & +(1-x)(1-z)\left(\psi_{2}+S_{1}-C_{2}-K_{2}+{\beta H}_{2}\right) \end{array}$$ (5)

When the carrier chooses to be dishonest, the expected return is:


$$\begin{array}{cc} E_{22}= & \;xz\left(\psi_{2}+R_{2}+{(1-\alpha )S}_{1}+G_{1}-\alpha T_{1}\right)+x(1-z)\left(\psi_{2}+R_{2}+G_{1}-\alpha T_{1}-K_{2}+\alpha H_{2}\right)\\ & +(1-x)z\left(\psi_{2}+G_{1}-\beta T_{1}+(1-\beta )S_{1}\right)+(1-x)(1-z)\left(\psi_{2}+G_{1}+(1-\beta )S_{1}-\beta T_{1}-K_{2}+\beta H_{2}\right) \end{array}$$ (6)

The expected income of the carrier is:


$$E_{2}=E_{21}y+E_{22}(1-y)$$ (7)

The replication dynamic equation of the carrier with an honest strategy is given by:


$$\begin{array}{cc} F_{C}(y) & =\frac{dy}{dt}=y(1-y)(E_{21}-E_{22})=y\left(E_{21}-E_{2}\right)\\ & =\left[{C_{2}+G}_{1}-{\beta (S}_{1}+T_{1})-(1-\beta )S_{1}x-\alpha xT_{1}+\beta xT_{1}+S_{1}xz-\alpha S_{1}xz\right]y(y-1\;) \end{array}$$ (8)

The calculation process of equation (8) is the same as that of equation (4).

#### Replication dynamic equation for shipper.

In the same way, when the shipper chooses to be honest, the expected return is:


$$\begin{array}{cc} E_{31}= & \;xy\left(\psi_{3}+R_{3}+S_{2}-L_{S}C_{3}\right)+x(1-y)\left(\psi_{3}+R_{3}+S_{2}-L_{S}C_{3}-L_{S}K_{1}+\alpha H_{1}\right)\\ & +(1-x)y\left(\psi_{3}+S_{2}-L_{S}C_{3}\right)+(1-x)(1-y)\left(\psi_{3}+S_{2}-L_{S}C_{3}-L_{S}K_{1}+\beta H_{1}\right) \end{array}$$ (9)

When the shipper chooses to be dishonest, the expected return is:


$$\begin{array}{cc} E_{32}= & \;xy\left(\psi_{3}+R_{3}+G_{2}{+(1-\alpha )S}_{2}-\alpha L_{S}T_{2}\right)+x(1-y)\left(\psi_{3}+R_{3}+G_{2}-\alpha L_{S}T_{2}-{L_{S}K}_{1}+\alpha H_{1}\right)\\ & +y(1-x)\left(\psi_{3}+G_{2}+{(1-\beta )S}_{2}-{\beta L_{S}T}_{2}\right)+(1-y)(1-x)\left(\psi_{3}+G_{2}-{L_{S}K}_{1}-\beta {L_{S}T}_{2}+\beta H_{1}\right) \end{array}$$ (10)

The expected income of the shipper is:


$$E_{3}=E_{31}z+E_{32}(1-z)$$ (11)

The following represents the dynamic equation for the shipper with honest strategy:


$$\begin{array}{cc} F_{S}(z) & =\frac{dz}{dt}=z(1-z)(E_{31}-E_{32})=z\left(E_{31}-E_{3}\right)\\ & =\left[L_{S}C_{3}+G_{2}-S_{2}-{\beta L_{S}T}_{2}+S_{2}y-\alpha x{L_{S}T}_{2}-\beta yS_{2}+\beta xL_{S}T_{2}-\alpha x{yS}_{2}+\beta x{yS}_{2}\right]z(z-1) \end{array}$$ (12)

The calculation process of equation (12) is the same as that of equation (4).

The analysis of formulas (4), (8), and (12) is of paramount importance in advancing the understanding and investigation of the mechanisms and behaviors observed in dynamic replication systems. These systems serve as the foundational components in various fields, and thus, the precise mathematical representation provided by these formulas is crucial in facilitating a comprehensive exploration of their intricate and evolving nature. By thoroughly examining these formulas, researchers can uncover latent principles and interaction mechanisms that govern dynamic replication systems. Moreover, these formulas offer invaluable insights for predicting and optimizing system behaviors, enabling researchers to make informed decisions and improve system performance.

Therefore, delving into the intricacies of these formulas holds immense significance for both academic research and practical implementations of dynamic replication systems. It promises to contribute to advancements in associated domains and foster progress in understanding and harnessing the potential of these dynamic replication systems.

### Jacobi matrix of the dynamical system

We can construct the Jacobian matrix by taking the first partial derivatives of equations (4), (8) and (12) with respect to the variables x, y and z, capturing the sensitivity of the system to small perturbations in these variables [39]. The Jacobian matrix can be seen from equation (13) below. The Jacobian provides insights into the local stability of equilibrium points. Specifically, the eigenvalues of the Jacobi matrix at each equilibrium point determine whether the system will return to equilibrium after small disturbances (negative eigenvalues indicate stability, while positive eigenvalues indicate instability). In the asymmetric game, the evolutionary stability strategy is regarded as a pure strategy if the conditions of asymmetric information are satisfied [40]. Thus, the asymptotic stability of the eight local equilibrium points, denoted as P_1_ (0,0,0), P_2_ (1,0,0), P_3_ (0,1,0), P_4_ (0,0,1), P_5_ (1,1,0), P_6_ (1,0,1), P_7_ (0,1,1), and P_8_ (1,1,1), satisfying the conditions $F_{NFP}(x)=0$, $F_{C}(y)=0$ and $F_{S}(z)=0$, is discussed.


$$J=[\begin{array}{ccc} \frac{dF_{NFP}(x))}{d(x)} & \frac{dF_{NFP}(x))}{d(y)} & \frac{dF_{NFP}(x))}{d(z)}\\ \frac{dF_{C}(y)}{d(x)} & \frac{dF_{C}(y)}{d(y)} & \frac{dF_{C}(y)}{d(z)}\\ \frac{dF_{S}(z)}{d(x)} & \frac{dF_{S}(z)}{d(y)} & \frac{dF_{S}(z)}{d(z)} \end{array}]=[\begin{array}{ccc} J_{11} & J_{12} & J_{13}\\ J_{21} & J_{22} & J_{23}\\ J_{31} & J_{32} & J_{33} \end{array}]$$ (13)

$$\begin{array}{cc} J_{11}= & \left(R_{1}+W-{L_{P}C}_{1}+{(\alpha -\beta )(L_{P}S_{1}+L_{P}S_{2}-L_{P}H}_{1}-L_{P}H_{2}+T_{1}+T_{2}+L_{P}H_{1}y+L_{P}H_{2}z-{L_{P}S}_{1}y-L_{P}S_{2}z-T_{1}y-T_{2}z)\right)\\ & (1-2x) \end{array}$$ (14)

$$J_{12}=(\alpha -\beta )\left({L_{P}H}_{1}{-L_{P}S}_{1}-T_{1}\right)x(1-x)$$ (15)

$$J_{13}=(\alpha -\beta )\left({L_{P}H}_{2}{-L_{P}S}_{2}-T_{2}\right)x(1-x)$$ (16)

$$J_{21}=\left[-(1-\beta )S_{1}-\alpha T_{1}+\beta T_{1}+S_{1}z-\alpha S_{1}z\right]y(y-1\;)$$ (17)

$$J_{22}=\left[{C_{2}+G}_{1}-{\beta (S}_{1}+T_{1})-(1-\beta )S_{1}x-\alpha xT_{1}+\beta xT_{1}+S_{1}xz-\alpha S_{1}xz\right](2y-1)\;$$ (18)

$$J_{23}=\left(S_{1}x-\alpha S_{1}x\right)y(y-1\;)$$ (19)

$$J_{31}=(\alpha -\beta )\left({L_{S}T}_{2}+{yS}_{2}\right)z(1-z)$$ (20)

$$J_{32}=(\beta +\alpha x-\beta x-1)S_{2}z(1-z)$$ (21)

$$J_{33}=\left(L_{S}C_{3}+G_{2}-S_{2}-{\beta L_{S}T}_{2}+S_{2}y-\alpha xL_{S}T_{2}-\beta yS_{2}+\beta x{L_{S}T}_{2}-\alpha x{yS}_{2}+\beta x{yS}_{2}\right)(2z-1)$$ (22)

### Stability analysis

In the evolutionary game model, stability is assessed by evaluating the eigenvalues derived from the Jacobian matrix. Stability occurs when the eigenvalues corresponding to the equilibrium points are negative, indicating that the system tends to return to equilibrium after small perturbations. The Jacobian matrix captures how each variable in the system—such as the strategic choices of the NFP, carrier, and shipper—affects the others. For instance, when the eigenvalues of an equilibrium point are all negative, the system is considered locally asymptotically stable. This means that any deviation from the equilibrium will decay over time, leading the system back to stability. Conversely, if one or more eigenvalues are positive, the system is unstable, and small deviations can drive the system away from equilibrium. Conditional stability arises when the eigenvalues are uncertain or include zero, indicating that the stability depends on specific conditions. By calculating the Jacobian matrix for each equilibrium point, we determined whether the system stabilizes when the NFP adopts data-driven mechanisms (DDM) or when it refrains from doing so.

Through plugging in eight stability points into Jacobian matrix above, it can get the eigenvalues $(\lambda_{1},\lambda_{2},\lambda_{3}\backslash )$and steady state which are presented in Table 3.

**Table 3:** Equilibrium points and their eigenvalues.

Equilibrium Points	Eigenvalue1s			Stability
	$\lambda_{1}$	$\lambda_{2}$	$\lambda_{3}$	
(0,0,0)	$S_{2}+{\beta L_{S}T}_{2}-L_{S}C_{3}-G_{2}$	${{\beta S}_{1}+{\beta T}_{1}-C}_{2}-G_{1}$	$R_{1}+W-L_{P}C_{1}+{(\alpha -\beta )(L_{P}S_{1}+{L_{P}S}_{2}-L_{P}H}_{1}-L_{P}H_{2}+T_{1}+T_{2})$	Conditional stable
(1,0,0)	$S_{2}+{\alpha L_{S}T}_{2}-L_{S}C_{3}-G_{2}$	$S_{1}+{\alpha T}_{1}-C_{2}-G_{1}$	${{L_{P}C}_{1}-R}_{1}-W+{(\alpha -\beta )(L_{P}H_{1}+{L_{P}H}_{2}-L_{P}S}_{1}-L_{P})$	Conditional stable
(0,1,0)	${\beta S}_{2}+{\beta L_{S}T}_{2}-{L_{S}C}_{3}-G_{2}$	$C_{2}+G_{1}-{\beta S}_{1}-{\beta T}_{1}$	$R_{1}+W-L_{P}C_{1}+{(\alpha -\beta )({L_{P}S}_{1}-L_{P}H}_{1}+T_{1})$	Conditional stable
(0,0,1)	${L_{S}C}_{3}+G_{2}-S_{2}-{\beta L_{S}T}_{2}$	${{\beta S}_{1}+{\beta T}_{1}-C}_{2}-G_{1}$	$R_{1}+W-L_{P}C_{1}+{(\alpha -\beta )(L_{P}S_{2}-L_{P}H}_{2}+T_{2})$	Conditional stable
(1,1,0)	${\alpha S}_{2}+{\alpha L_{S}T}_{2}-L_{S}C_{3}-G_{2}$	$C_{2}+G_{1}-S_{1}-{\alpha T}_{1}$	$L_{P}{C_{1}-R}_{1}-W+{(\alpha -\beta )(L_{P}H_{2}-L_{P}S}_{2}-T_{2})$	Conditional stable
(1,0,1)	$L_{S}C_{3}+G_{2}-S_{2}-{\alpha L_{S}T}_{2}$	${{\alpha S}_{1}+{\alpha T}_{1}-C}_{2}-G_{1}$	$L_{P}{C_{1}-R}_{1}-W+{(\alpha -\beta )({L_{P}H}_{1}-L_{P}S}_{1}-T_{1})$	Conditional stable
(0,1,1)	$L_{S}C_{3}+G_{2}-\beta S_{2}-{\beta L_{S}T}_{2}$	$C_{2}+G_{1}-{\beta S}_{1}-{\beta T}_{1}$	$R_{1}+W-L_{P}C_{1}$	Conditional stable
(1,1,1)	${L_{S}C}_{3}+G_{2}-\alpha S_{2}-{\alpha L_{S}T}_{2}$	$C_{2}+G_{1}-{\alpha S}_{1}-{\alpha T}_{1}$	$L_{P}{C_{1}-R}_{1}-W$	Conditional stable

For all eight equilibrium points, the corresponding eigenvalues are all not defined by positive or negative sign, and they are conditional stable points.

If $S_{2}+{\beta L_{S}T}_{2}<{L_{S}C}_{3}+G_{2},$ ${{\beta S}_{1}-C}_{2}<G_{1}-{\beta T}_{1}$ and${\;R}_{1}+W-L_{P}C_{1}<{(\alpha -\beta )(L_{P}H}_{1}+L_{P}H_{2}-L_{P}S_{1}-L_{P}S_{2}-T_{1}-T_{2}$hold true, then the system can achieve local stability. In this scenario, the stable strategy is indicated as P_1_ (0,0,0). For the NFP, $R_{1}+W-L_{P}C_{1}<{(\alpha -\beta )(L_{P}H}_{1}+L_{P}H_{2}-L_{P}S_{1}-L_{P}S_{2}-T_{1}-T_{2})$ means that the net revenue increase after implementation DDM is less than the difference between the remediation cost and the subsidy. In this case, the NFP has no incentive to choose DDM and will stabilize at the TMM strategy. For the carrier, ${{\beta S}_{1}-C}_{2}<G_{1}-{\beta T}_{1}$ indicates that the carrier earns less revenue under an honest strategy compared to a dishonest one when the NFP uses TMM. Therefore, the carrier will opt for dishonest strategy. For the shipper, $S_{2}+{\beta L_{S}T}_{2}<L_{S}C_{3}+G_{2}$ is equivalent to $S_{2}-L_{S}C_{3}<G_{2}-{\beta L_{S}T}_{2}.$ This means that the shipper earns less revenue under an honest strategy than under a dishonest one under TMM. In this case, the shipper has no incentive to choose honest strategy and will therefore stabilize at the dishonest strategy. If $S_{2}+{\alpha L_{S}T}_{2}<L_{S}C_{3}+G_{2},$ ${S_{1}-C}_{2}<G_{1}-{\alpha T}_{1}$ and$\;R_{1}+W-C_{1}>{(\alpha -\beta )(H}_{1}+H_{2}-S_{1}-S_{2}-T_{1}-T_{2}$are satisfied, then the system can achieve local stability. In this case, the stable strategy for the system is denoted as P_2_ (1,0,0). For the NFP, $R_{1}+W-C_{1}>{(\alpha -\beta )(H}_{1}+H_{2}-S_{1}-S_{2}-T_{1}-T_{2})$ means that the net revenue increase after implementing DDM exceeds the difference between the remediation cost and the subsidy. In this case, the NFP will choose DDM strategy and therefore stabilize at that choice. For the carrier, ${S_{1}-C}_{2}<G_{1}-{\alpha T}_{1}$ indicates that the carrier earns less revenue under an honest strategy than under a dishonest strategy when the NFP uses DDM. Thus, the carrier will again choose the dishonest strategy. For the shipper, $S_{2}+{\alpha L_{S}T}_{2}<L_{S}C_{3}+G_{2}$ is equivalent to $S_{2}-L_{S}C_{3}<G_{2}-{\alpha L_{S}T}_{2}$, indicating that the shipper earns less revenue under honest strategy than under a dishonest one when the NFP uses DDM. Consequently, the shipper has no incentive to adopt an honest strategy and will stabilize at the dishonest strategy.

If ${\alpha S}_{2}-L_{S}C_{3}<G_{2}-\beta L_{S}T_{2},$ ${{\beta S}_{1}-C}_{2}>G_{1}-{\beta T}_{1}$ and $R_{1}+W-L_{P}C_{1}<{(\alpha -\beta )(L_{P}H}_{1}-{L_{P}S}_{1}-T_{1}$are met, the optimal evolutionary strategy is identified as P_3_ (0,1,0). For the NFP, the condition $R_{1}+W-L_{P}C_{1}<{(\alpha -\beta )(L_{P}H}_{1}-{L_{P}S}_{1}-T_{1})$ means that the net increase in revenue from implementing DDM is less than the difference between the remediation cost and the subsidy to the carrier. Therefore, the NFP has no incentive to choose the DDM and will stabilize at the TMM strategy. For the carrier, the condition ${{\beta S}_{1}-C}_{2}>G_{1}-{\beta T}_{1}$ indicates that the carrier earns more revenue under an honest strategy than under a dishonest one when the NFP uses TMM. As a result, the carrier will opt for the honest strategy. For the shipper, the condition ${\beta S}_{2}-L_{S}C_{3}<G_{2}-\beta L_{S}T_{2}$ indicates that the shipper earns less revenue under an honest strategy than under a dishonest one when the NFP uses TMM. In this situation, the shipper has no incentive to adopt the honest strategy and will stabilize at the dishonest strategy.

If $S_{2}-L_{S}C_{3}>G_{2}-{\beta L_{S}T}_{2},$ ${{\beta S}_{1}-C}_{2}<G_{1}-{\beta T}_{1}$ and$\;R_{1}+W-L_{P}C_{1}<{(\alpha -\beta )(L_{P}H}_{2}-L_{P}S_{2}-T_{2}$are fulfilled by the evolution system, the system can attain local stability. In this case, the stable strategy for the system is denoted as P_4_ (0,0,1). For the NFP, $R_{1}+W-L_{P}C_{1}<{(\alpha -\beta )(L_{P}H}_{2}-L_{P}S_{2}-T_{2}$indicates that the net increase in revenue after the implementation of DDM by the NFP is less than the difference between the remediation cost and the subsidy to the shipper. In this case, the NFP will don’t choose DDM strategy and therefore stabilize at the TMM strategy. For the carrier, ${{\beta S}_{1}-C}_{2}<G_{1}-{\beta T}_{1}$ indicates that the carrier gets less revenue under honest strategy than under dishonest strategy when the NFP uses TMM, in which case the carrier will choose dishonest strategy. For the shipper, $S_{2}-L_{S}C_{3}>G_{2}-{\beta L_{S}T}_{2}\;$indicates that the shipper gets more revenue under honest strategy than under dishonest strategy when the NFP uses TMM. In this case, the shipper will choose honest strategy and therefore stabilize at the honest strategy.

If ${\alpha S}_{2}-{L_{S}C}_{3}<G_{2}-\alpha L_{S}T_{2},$ ${S_{1}-C}_{2}>G_{1}-{\alpha T}_{1}$ and $R_{1}+W-L_{P}C_{1}>{(\alpha -\beta )(L_{P}H}_{2}-L_{P}S_{2}-T_{2}$are fulfilled, in such a scenario, the optimal evolutionary strategy for the system can be identified as P_5_ (1,1,0). For the NFP, $R_{1}+W-L_{P}C_{1}>{(\alpha -\beta )(L_{P}H}_{2}-L_{P}S_{2}-T_{2})$ indicates that the net increase in revenue after the implementation of DDM by the NFP is more than the difference between the remediation cost and the subsidy to the carrier. In this case, the NFP tend to choose the DDM and will therefore stabilize at the DDM strategy. For the carrier, ${S_{1}-C}_{2}>G_{1}-{\alpha T}_{1}\;$indicates that the carrier gets more revenue under honest strategy than under dishonest strategy when the NFP uses DDM, in which case the carrier will choose honest strategy. For the shipper, ${\alpha S}_{2}-L_{S}C_{3}<G_{2}-\alpha {L_{S}T}_{2}$ indicates that the shipper gets less revenue under honest strategy than under dishonest strategy when the NFP uses DDM. In this case, the shipper has no incentive to choose honest strategy and will therefore stabilize at the dishonest strategy.

If $S_{2}-{L_{S}C}_{3}>G_{2}-{\alpha L_{S}T}_{2},$ ${{\alpha S}_{1}-C}_{2}<G_{1}-{\alpha T}_{1}$ and$\;R_{1}+W-L_{P}C_{1}>{(\alpha -\beta )(L_{P}H}_{1}-L_{P}S_{1}-T_{1}$are fulfilled, the system can attain local stability. In this case, the stable strategy for the system is denoted as P_6_ (1,0,1). For the NFP, $R_{1}+R_{1}+W-L_{P}C_{1}>{(\alpha -\beta )(L_{P}H}_{1}-L_{P}S_{1}-T_{1}$indicates that the net increase in revenue after the implementation of DDM by the NFP is more than the difference between the remediation cost and the subsidy to the carrier. In this case, the NFP will tend to choose DDM strategy and therefore stabilize at the DDM strategy. For the carrier, ${{\alpha S}_{1}-C}_{2}<G_{1}-{\alpha T}_{1}$ indicates that the carrier gets less revenue under honest strategy than under dishonest strategy when the NFP uses DDM, in which case the carrier will choose dishonest strategy. For the shipper, $S_{2}-L_{S}C_{3}>G_{2}-{\alpha L_{S}T}_{2}\;$indicates that the shipper gets more revenue under honest strategy than under dishonest strategy when the NFP uses DDM. In this case, the shipper will choose honest strategy and therefore stabilize at the honest strategy.

If ${\beta S}_{2}-L_{S}C_{3}>G_{2}-\beta L_{S}T_{2},$ ${{\beta S}_{1}-C}_{2}>G_{1}-{\beta T}_{1}$ and $R_{1}+W-C_{1}<0\;$are fulfilled by the evolution system, in such a scenario, the optimal evolutionary strategy for the system can be identified as P_7_ (0,1,1). For the NFP, $R_{1}+W-C_{1}<0$ indicates that the net revenue after the implementation of DDM by the NFP is negative. In this case, the NFP definitely has no incentive to choose the DDM and will therefore stabilize at the TMM strategy. For the carrier, ${{\beta S}_{1}-C}_{2}>G_{1}-{\beta T}_{1}$ indicates that the carrier gets more revenue under honest strategy than under dishonest strategy when the NFP uses TMM, in which case the carrier will choose honest strategy. For the shipper, ${\beta S}_{2}-L_{S}C_{3}>G_{2}-\beta {L_{S}T}_{2}$ indicates that the shipper gets more revenue under honest strategy than under dishonest strategy when the NFP uses TMM. In this case, the shipper tends to choose honest strategy and will therefore stabilize at the honest strategy.

If ${\alpha S}_{2}-L_{S}C_{3}>G_{2}-\alpha L_{S}T_{2},$ ${{\alpha S}_{1}-C}_{2}>G_{1}-{\alpha T}_{1}\;$and $R_{1}+W-L_{P}C_{1}>0\;$are fulfilled, the optimal evolutionary strategy for the system is identified as P_8_ (1,1,1). This equilibrium point reflects a beneficial strategy for all three participants in the game and bolsters the system’s ongoing development. For the NFP, the condition $R_{1}+W-L_{P}C_{1}>0$ indicates that the net revenue after implementing DDM is positive. In this situation, it is profitable for the NFP to choose DDM, and thus it will stabilize at the DDM strategy. For the carrier, ${{\alpha S}_{1}-C}_{2}>G_{1}-{\alpha T}_{1}$ indicates that the carrier receives more revenue under an honest strategy than under a dishonest one when the NFP uses DDM. As a result, the carrier will choose the honest strategy. For the shipper, ${\alpha S}_{2}-L_{S}C_{3}>G_{2}-\alpha {L_{S}T}_{2}\;$indicates that the shipper also earns more revenue under an honest strategy than under a dishonest one when the NFP uses DDM. Consequently, the shipper is likely to adopt the honest strategy and will stabilize at that choice.

The previous analysis suggests that EGM in NFP supply chain management offers a framework for the NFP, carriers and shippers to make informed strategic decisions. The findings show that the NFP can use data-driven mechanisms to encourage stable and cooperative behavior among carriers and shippers. By setting appropriate levels of subsidies ($S_{1}$, $S_{2}$) and penalties ($T_{1}$, $T_{2}$), the NFP can create incentives that encourage these stable outcomes and promote long-term cooperation. However, if the NFP does not implement DDM, the model indicates that dishonest strategies may become stable, threatening the integrity of the system. The NFP as it provides a pathway to reduce the prevalence of opportunistic behavior in the supply chain, which in turn enhances operational efficiency. By setting appropriate subsidy and penalty levels, the NFP can create incentives that shift the system towards these stable outcomes, ensuring long-term cooperation. However, if the NFP does not implement DDM, the model indicates that dishonest strategies may become stable, threatening the integrity of the system. The NFP must consider the initial costs of implementing DDM against the long-term benefits of establishing a stable and cooperative supply chain. The results suggest that investing in data-driven mechanisms not only promotes stability but also helps the NFP mitigate risks associated with dishonest behavior among stakeholders. Moreover, the benefits for shippers and carriers from choosing honest strategies should outweigh any additional gains they might receive from dishonest behavior. The NFP can achieve this by establishing reasonable subsidies and penalties, guiding them away from dishonest practices towards honest trading, thus steering the system toward a stable strategy.

### Dynamics trend of NFP

According to equation F(x)=0, we can obtain the expression of the steady state dividing line. If $R_{1}+W-L_{P}C_{1}+{(\alpha -\beta )({L_{P}S}_{1}+L_{P}S_{2}-L_{P}H}_{1}-L_{P}H_{2}+T_{1}+T_{2}+{L_{P}H}_{1}y+L_{P}H_{2}z-L_{P}S_{1}y-L_{P}S_{2}z-T_{1}y-T_{2}z)=0$, i.e., $y=y^{*}=-\frac{R_{1}+W-L_{P}C_{1}+{(\alpha -\beta )({L_{P}S}_{1}+L_{P}S_{2}-L_{P}H}_{1}-L_{P}H_{2}+T_{1}+T_{2}+{L_{P}H}_{2}z-L_{P}S_{2}z-T_{2}z)}{\left(\alpha -\beta )({L_{P}H}_{1}-L_{P}S_{1}-T_{1}\right)}$, then $F(x)={=F}^{\prime }(x)==0$, it indicates that the system is stable. In other words, if $y=y^{*}=-\frac{R_{1}+W-L_{P}C_{1}+{(\alpha -\beta )({L_{P}S}_{1}+L_{P}S_{2}-L_{P}H}_{1}-L_{P}H_{2}+T_{1}+T_{2}+{L_{P}H}_{2}z-L_{P}S_{2}z-T_{2}z)}{\left(\alpha -\beta )({L_{P}H}_{1}-L_{P}S_{1}-T_{1}\right)}$, then the NFP’ strategies tend to stabilize. The policy implemented by the NFP has no direct influence on the direction of the system.

When $y>\;y^{*}=-\frac{R_{1}+W-L_{P}C_{1}+{(\alpha -\beta )({L_{P}S}_{1}+L_{P}S_{2}-L_{P}H}_{1}-L_{P}H_{2}+T_{1}+T_{2}+{L_{P}H}_{2}z-L_{P}S_{2}z-T_{2}z)}{\left(\alpha -\beta )({L_{P}H}_{1}-L_{P}S_{1}-T_{1}\right)}$, combining equations (4) and (14), the system can be stabilized when $x^{*}=1$. In other words, if the NFP opts to choose DDM, the EGM can achieve a steady state.

When $y<y^{*}=-\frac{R_{1}+W-L_{P}C_{1}+{(\alpha -\beta )({L_{P}S}_{1}+L_{P}S_{2}-L_{P}H}_{1}-L_{P}H_{2}+T_{1}+T_{2}+{L_{P}H}_{2}z-L_{P}S_{2}z-T_{2}z)}{\left(\alpha -\beta )({L_{P}H}_{1}-L_{P}S_{1}-T_{1}\right)}$, combining equations (4) and (14), the system can be stabilized when $x^{*}=0$. In other words, if the NFP don’t choose DDM, the EGM can achieve a steady state.

**Fig 2:**
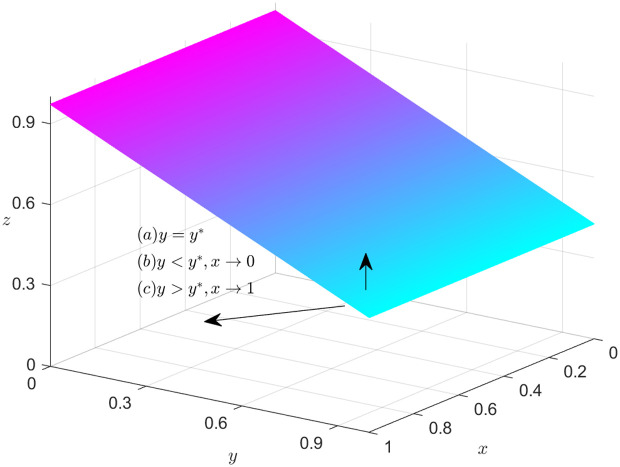
Dynamics trend of the NFP.

Fig 2 depicts the decision-making dynamics evolution of the NFP. Given that the joint space, defined by variables y and z, divides the feasible domain space into two adjacent parts, it is important to highlight the significance of this joint space by marking it in a distinguished color, such as purple. Meanwhile, the NFP ‘s mixed strategy space can be represented as the set of values for x, where x belongs to the interval [0,1]. To emphasize the trend of x within this range, an arrow can be utilized to indicate its direction. When it is in the lower part of the plane, x approaches 0 and is stable at 0. In this case, no-DDM is optimal for the NFP. When the system runs to the upper part of the plane, x approaches 1 and stabilizes at 1 point, thus, the best course of action for the NFP is to opt for DDM.

### Dynamics trend of the carrier

According to equation F(y)=0, we can obtain the expression of the steady state dividing line. If ${L_{S}C}_{3}-\psi_{2}z-S_{1}x-Rz+\beta \psi_{2}z+\alpha Rz=0$*,* i.e*.,* $z=z^{*}=-\frac{{C_{2}+G}_{1}-{\beta (S}_{1}+T_{1})-(1-\beta )S_{1}x-\alpha xT_{1}+\beta xT_{1}}{S_{1}x(1-\alpha )}$*,* then $F(y)={=F}^{\prime }(y)==0$, it indicates that the system is stable. In other words, if $z=z^{*}=-\frac{{C_{2}+G}_{1}-{\beta (S}_{1}+T_{1})-(1-\beta )S_{1}x-\alpha xT_{1}+\beta xT_{1}}{S_{1}x(1-\alpha )}$, then the carrier’ strategies tend to stabilize. The choice implemented by the carrier has no direct influence on the direction of the system.

When ${C_{2}+G}_{1}-{\beta (S}_{1}+T_{1})-(1-\beta )S_{1}x-\alpha xT_{1}+\beta xT_{1}+S_{1}xz-\alpha S_{1}xz\neq 0$, if F(y)=0, then $y^{*}=$0, $y^{*}=$1. These are two stable points for recyclers.

When $z>z^{*}=-\frac{{C_{2}+G}_{1}-{\beta (S}_{1}+T_{1})-(1-\beta )S_{1}x-\alpha xT_{1}+\beta xT_{1}}{S_{1}x(1-\alpha )}$, combining equations (8) and (18), the system can be stabilized when $y^{*}=1$. In other words, if the carrier chooses honest strategy, the EGM can achieve a steady state.

When $z<z^{*}=-\frac{{C_{2}+G}_{1}-{\beta (S}_{1}+T_{1})-(1-\beta )S_{1}x-\alpha xT_{1}+\beta xT_{1}}{S_{1}x(1-\alpha )}$, combining equations (8) and (18), the system can be stabilized when $y^{*}=0$. In other words, if the carrier doesn’t choose honest strategy, the EGM can achieve a steady state.

**Fig 3:**
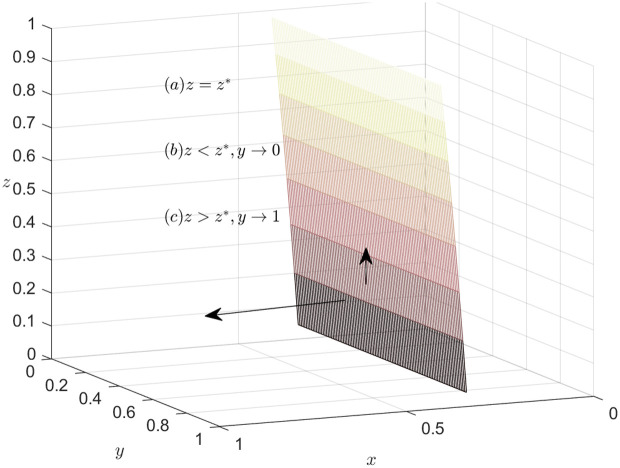
Dynamics trend of the carrier.

Fig 3 depicts the decision-making dynamics evolution of the carrier. Given that the joint space, defined by variables x and z, divides the feasible domain space into two adjacent parts, it is important to highlight the significance of this joint space by marking it in a distinguished color, such as pink. Meanwhile, the carrier’s mixed strategy space can be represented as the set of values for y, where y belongs to the interval [0,1]. To emphasize the trend of y within this range, an arrow can be utilized to indicate its direction. When it is in the lower part of the plane, y approaches 0 and is stable at 0. In this case, choosing dishonest strategy is optimal for the carrier. When the system runs to the upper part of the plane, y approaches 1 and stabilizes at 1 point, thus, the best course of action for the carrier is to opt for honest strategy.

### Dynamics trend of the shipper

According to Formula (12), if $\;L_{S}C_{3}+G_{2}-S_{2}-{\beta L_{S}T}_{2}+S_{2}y-\alpha x{L_{S}T}_{2}-\beta yS_{2}+\beta xL_{S}T_{2}-\alpha x{yS}_{2}+\beta x{yS}_{2}=0$, i.e., $\begin{array}{c} x=x^{*}=\frac{{L_{S}C}_{3}+G_{2}-S_{2}-{\beta L_{S}T}_{2}+S_{2}y-\beta yS_{2}}{(\alpha -\beta )\left(L_{S}T_{2}+{yS}_{2}\right)} \end{array}$, then $F(z)={=F}^{\prime }(z)==0$, it indicates that the system is stable. In other words, if $\begin{array}{c} x=x^{*}=\frac{{L_{S}C}_{3}+G_{2}-S_{2}-{\beta L_{S}T}_{2}+S_{2}y-\beta yS_{2}}{(\alpha -\beta )\left(L_{S}T_{2}+{yS}_{2}\right)} \end{array}$, then the shipper’s strategies tend to stabilize. The choice implemented by the shipper has no direct influence on the direction of the system. When $\;L_{S}C_{3}+G_{2}-S_{2}-{\beta L_{S}T}_{2}+S_{2}y-\alpha x{L_{S}T}_{2}-\beta yS_{2}+\beta xL_{S}T_{2}-\alpha x{yS}_{2}+\beta x{yS}_{2}\neq 0$, if F(z)=0, then $z^{*}=$ 0, $z^{*}=$1. When $\begin{array}{c} \;x>x^{*}=\frac{{L_{S}C}_{3}+G_{2}-S_{2}-{\beta L_{S}T}_{2}+S_{2}y-\beta yS_{2}}{(\alpha -\beta )\left(L_{S}T_{2}+{yS}_{2}\right)} \end{array}$*,* combining equations (12) and (22), the system can be stabilized when $z^{*}=1$. In other words, if the shipper opts to choose honest strategy, the EGM can achieve a steady state. When $\begin{array}{c} x<x^{*}=\frac{{L_{S}C}_{3}+G_{2}-S_{2}-{\beta L_{S}T}_{2}+S_{2}y-\beta yS_{2}}{(\alpha -\beta )\left(L_{S}T_{2}+{yS}_{2}\right)} \end{array}$*,* combining equations (12) and (22), the system can be stabilized when $z^{*}=0$. In other words, if the shipper opts to choose dishonest strategy, the EGM can achieve a steady state.

**Fig 4:**
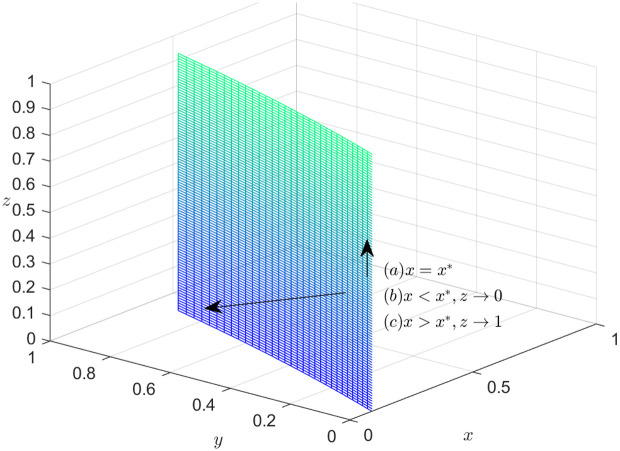
Dynamics trend of the shipper.

Fig 4 depicts the decision-making dynamics evolution of the shipper. Given that the joint space, defined by variables x and y, divides the feasible domain space into two adjacent parts, it is important to highlight the significance of this joint space by marking it in a distinguished color, such as pink. Meanwhile, the carrier’s mixed strategy space can be represented as the set of values for z, where z belongs to the interval [0,1]. To emphasize the trend of z within this range, an arrow can be utilized to indicate its direction. When it is in the lower part of the plane, z approaches 0 and is stable at 0. In this case, choosing dishonest strategy is optimal for the shipper. When the system runs to the upper part of the plane, z approaches 1 and stabilizes at 1 point, thus, the best course of action for the shipper is to opt for honest strategy.

## Simulation

In this study, we employ an agent-based simulation model to examine the dynamic interactions among the NFP, carrier, and shipper. This approach is particularly suited to capturing the complex and adaptive behaviors of stakeholders, as it allows for individual decision-making based on the evolutionary game framework. The model represents each stakeholder as an autonomous agent with specific strategy sets, payoffs, and adaptation rules derived from the replication dynamic equations.

The simulation was implemented in MATLAB, with key parameters calibrated to reflect realistic market conditions. It is assumed that array 1: $\alpha =0.85,\;\beta =0.68,\;W=80,\;R_{1}=800,\;P=20,{\;S}_{1}=100,\;{\;S}_{2}=70,\;{\;H}_{1}=240,\;{\;H}_{2}=300,{\;T}_{1}=300,\;{\;T}_{2}=350,{\;C}_{1}=180,\;{\;C}_{2}=120,\;{\;C}_{3}=100,\;{\;G}_{1}=180,\;{\;G}_{2}=140,\;L_{P}=1.2,\;L_{S}=1.6.\;$ The parameters used in the simulation were carefully selected based on a combination of literature review, industry reports, and realistic case studies within the logistics sector. To ensure robustness, 1,000 independent simulation runs were performed for each scenario, with each run consisting of 50 iterations. This number of iterations was chosen to allow the system to reach a stable equilibrium or reveal significant trends in strategy evolution. The agents’ initial probabilities for selecting specific strategies were randomly assigned within predefined ranges, ensuring diversity in starting conditions.

Each simulation run generated outputs such as the evolution of strategy probabilities, the stability of equilibrium points, and the impact of parameters like subsidies, penalties, and detection probabilities on cooperative behavior. Sensitivity analyses were also conducted to assess the effects of varying initial conditions and parameter values on the overall system dynamics.

### The impact of the initial value on EGM

**Fig 5:**
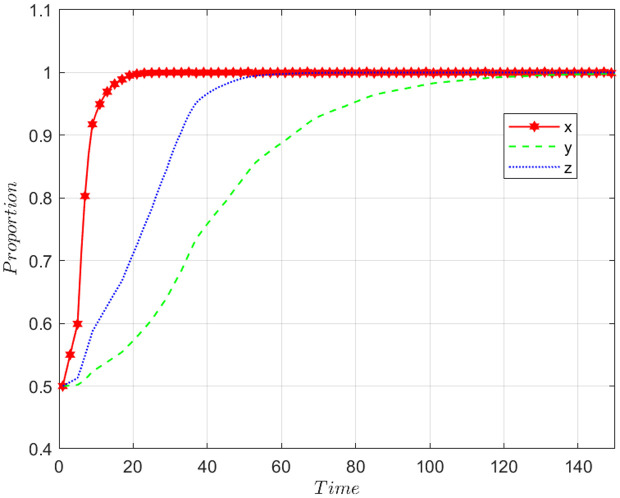
Effect of initial setting.

Let $x_{0},\;y_{0}$ and $z_{0}\;$are the initial points of x, y and z, respectively. Here, setting $x_{0}=y_{0}=\;z_{0}=0.5$. The following section will explore the influence of various starting points on the choice patterns of the three entities. Fig 5 shows how, from the initial point, the NFP, carrier and shipper dynamically evolve toward the stable point (DDM, honest, honest). The NFP reaches equilibrium more quickly, largely due to the strong data collection, real-time feedback, and optimized decision-making provided by the DDM. These features improve user experience, reduce costs, and enhance technological innovation, helping the NFP establish itself in a competitive market and achieve sustainable growth. Shippers benefit from improved service and reduced costs under DDM strategy. The powerful data forecasting, monitoring, and process management capabilities of the DDM create transparency and accountability, which discourage opportunistic behavior. As a result, shippers are more inclined to choose honest transactions, trusting that the system will promote fair practices. Carriers, observing the NFP’s and shippers’ choices, are gradually influenced to adopt honest trading strategies as well.

### The impact of the initial probability on EGM

**Fig 6:**
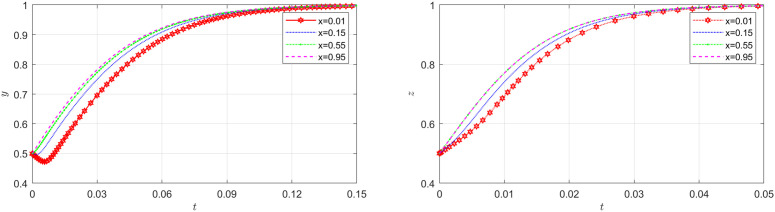
The impact of initial probability of the NFP on the carrier and shipper. (a) Effect of initial probability of the NFP on the carrier (b) Effect of initial probability of the NFP on the shipper.

**Fig 7:**
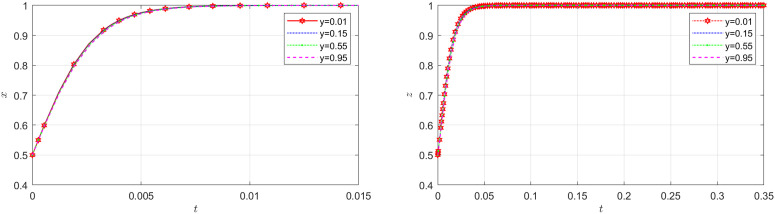
The impact of initial probability of the carrier on the NFP and the shipper.

**Fig 8:**
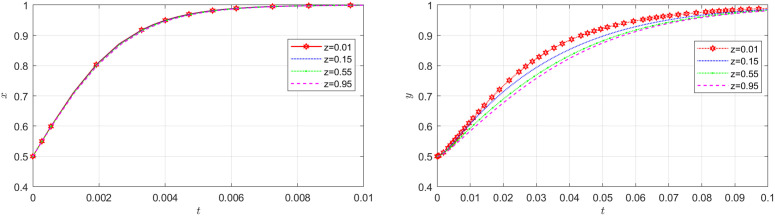
The impact of initial probability of the shipper on the NFP and the carrier.

This section discusses the influence of the initial value selected by each player on the equilibrium under other factors unchanged. The outcomes are illustrated in Figs 6–8.

Fig 6 illustrates how the initial probability of the NFP choosing DDM influences the evolution of carrier and shipper strategies. The diagram located to the left and right in Fig 6 show that as the probability of the NFP choosing DDM increases, carriers and shippers are quicker to adopt honest strategies. However, once this probability reaches a certain threshold, further increases have little effect on their behavior. Therefore, as long as the initial probability of the NFP choosing DDM exceeds a certain value, both shippers and carriers will place greater trust in the NFP. Expecting the NFP to continue choosing DDM, they are less likely to take risks or act dishonestly and are more inclined to pursue honest strategies.

Fig 7 shows how the initial probability of the carrier choosing honest behavior affects the evolution of the NFP and shipper’s strategies. According to Fig 7, the initial probability of the carrier does not bring about significant changes on the system. Similarly, according to the diagram located to the left in Fig 8a, the initial probability of the shipper does not play a substantial role in the NFP supply chain. This finding is readily comprehensible when examined within the context of a real supply chain system. In an NFP supply chain, the NFP entity holds dominance, while other entities are more passively influenced. This is primarily due to the fact that the decision of whether or not to employ DDM lies in the hands of the NFP entity, subsequently leading to changes in decisions made by other entities.

Fig 8 illustrates how the initial probability of the shipper choosing honest behavior affects the evolution of the NFP and carrier’s strategies. The diagram located to the right in Fig 8 demonstrates that as the shipper’s probability of adopting an honest strategy increase, it becomes increasingly challenging for the carrier to achieve stability in adopting an honest strategy. However, beyond a certain threshold, further changes in this probability do not significantly impact the carrier’s behavior. This can be attributed to the fact that when carriers observe shippers exhibiting a tendency towards honest behavior, they are more likely to exploit any loopholes in order to engage in dishonest transactions and obtain substantial profits.

### The impact of parameters on EGM

**Fig 9:**
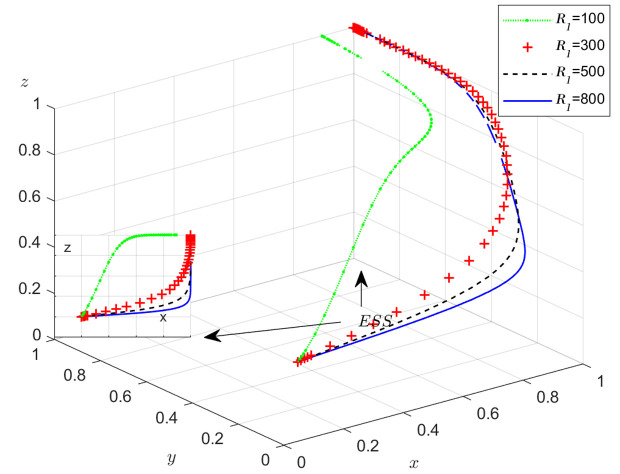
The influence of the benefit ${\;R}_{1}\;$ **from DDM.**

**Fig 10:**
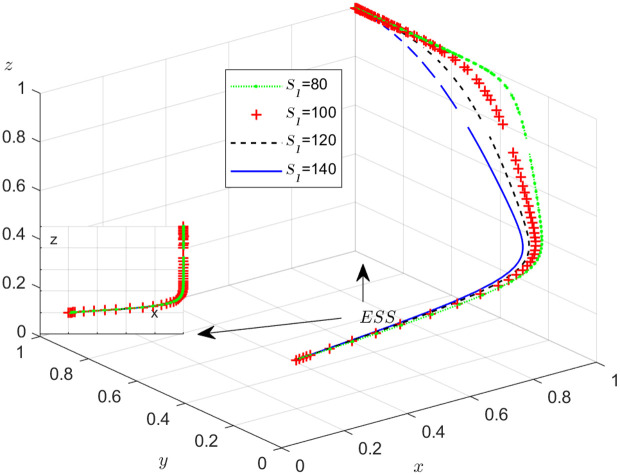
The influence of the subsidy ${\;S}_{1}$.

**Fig 11:**
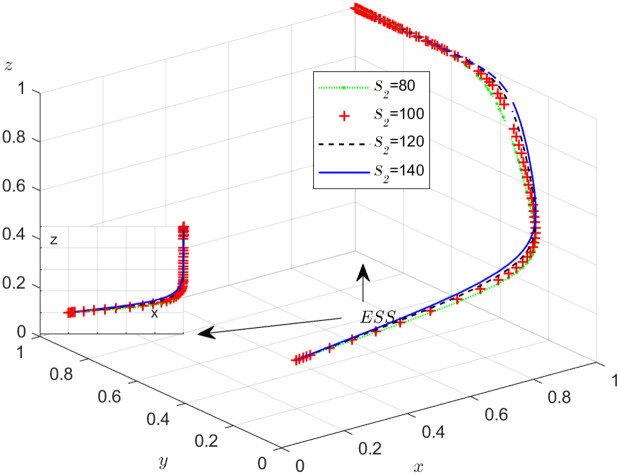
The influence of the subsidy ${\;S}_{2}\;$.

**Fig 12:**
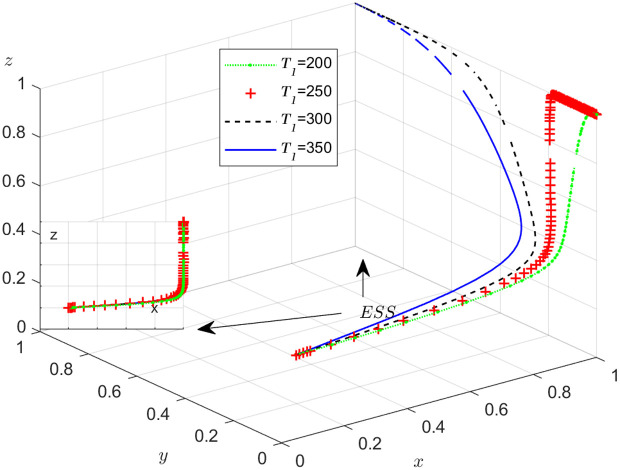
The influence of penalty $T_{1}$.

**Fig 13:**
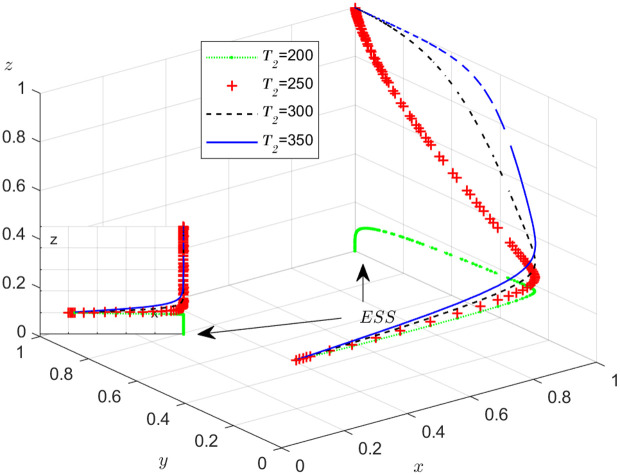
The influence of penalty $T_{2}$.

**Fig 14:**
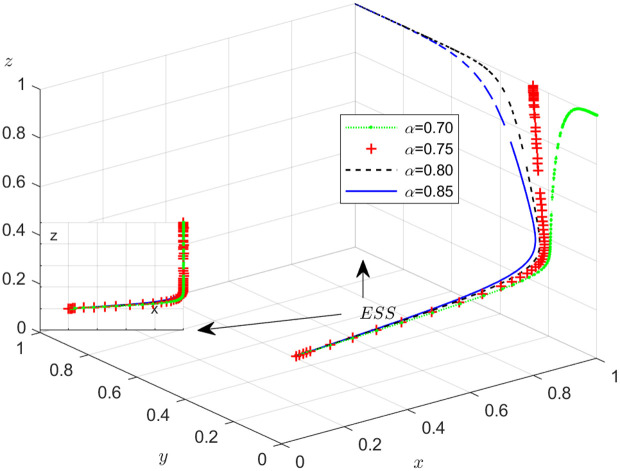
The influence of the probability α **of the carrier’s or the shipper’s dishonest strategy discovered by the NFP’s choosing DDM.**

**Fig 15:**
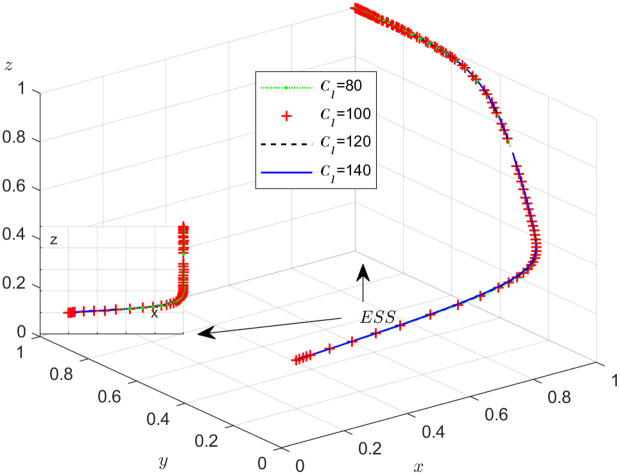
The influence of extra cost $C_{1}$ **for the NFP choosing DDM.**

**Fig 16:**
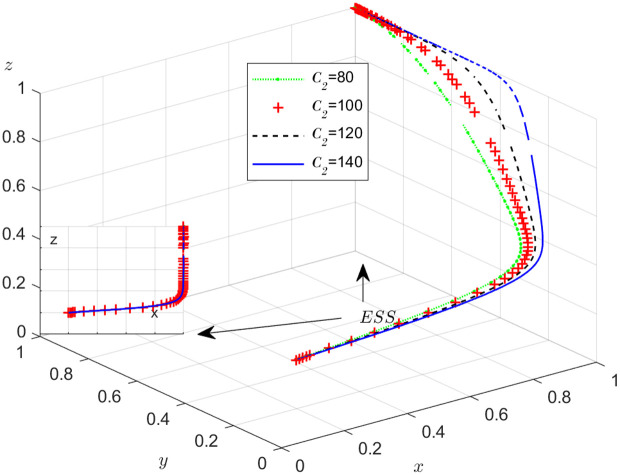
Effect of extra cost $C_{2}$ **on the carrier’s adoption of the honest strategy.**

**Fig 17:**
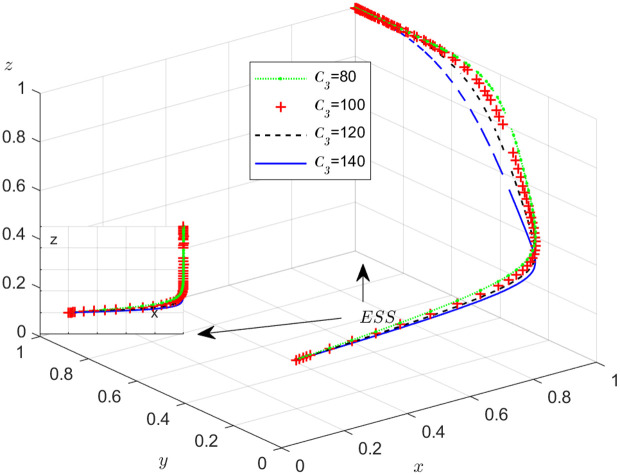
The influence of extra cost $C_{3}$ **for the shipper’s honest strategy.**

**Fig 18:**
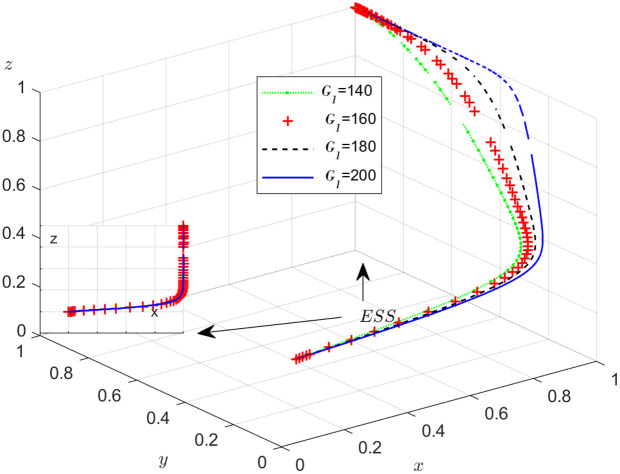
The influence of the bonus from dishonest of the carrier $G_{1}$.

**Fig 19:**
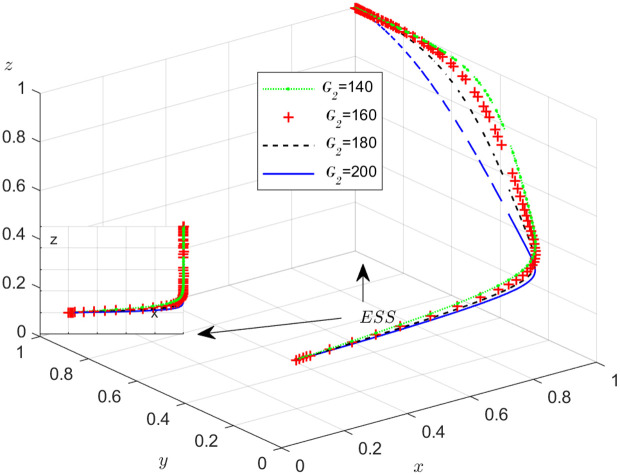
The influence of the bonus from dishonest of the shipper $G_{2}$.

**Fig 20:**
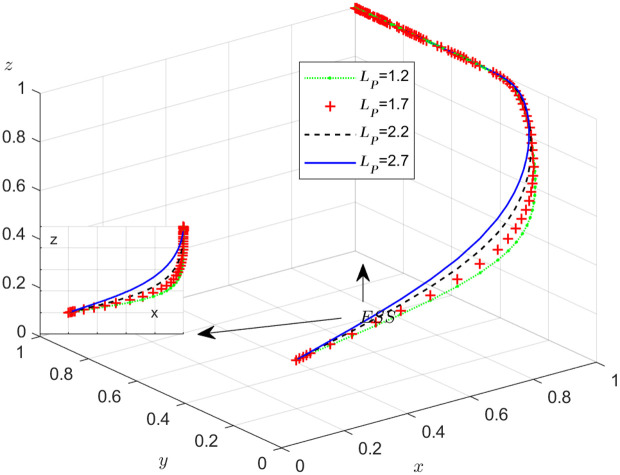
The influence of loss preference coefficient $L_{P}$.

**Fig 21:**
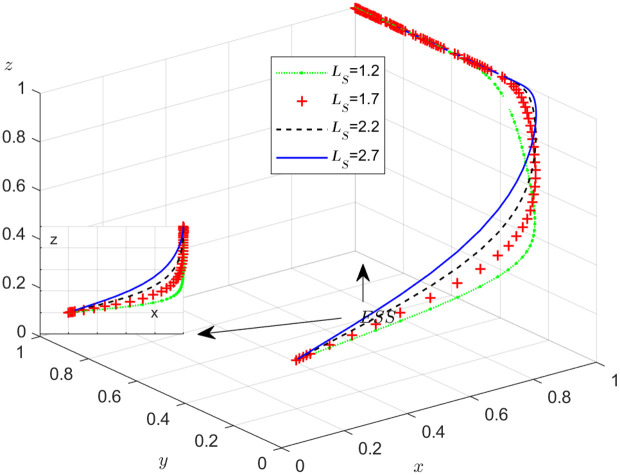
The influence of loss preference coefficient $L_{S}$.

The objective of this research is to gain a more profound insight into the ways in which different parameters affect the individuals involved in the extensive EGM. These parameters are grouped into six distinct scenarios, allowing for a detailed analysis of their respective effects. The simulation results of 50 times evolution of dynamic equations over time were replicated. First, the impact of the benefits $R_{1}$ gained from DDM is examined. The values of $R_{1}$ were set at 100, 300, 500, and 800, as shown in Fig 9. Next, to analyze the influence of subsidies $S_{1}$ and ${\;S}_{2}$, these were assigned values of 80, 100, 120, and 140, respectively. The simulation trends for these scenarios are shown in Figs 10 and 11. To assess the impact of penalties$\;T_{1}\;$and$\;T_{2}$ on dishonest strategy, the values of $T_{1}/{\;T}_{2}$ were set at 200, 250, 300, and 350. The resulting simulation trends are illustrated in Figs 12 and 13. The influence of the probability α of detecting dishonesty by the NFP under DDM was also examined. The values of α were set at 0.70, 0.75, 0.80, and 0.85, as shown in Fig 14. Additionally, the effect of extra costs $C_{i}(where\;i=1,2,3)$ was analyzed, with${\;C}_{i}$ assigned values of 80, 100, 120, and 140. The simulation results can be seen in Figs 15–17. To investigate the influence of bonuses $G_{1}$and $G_{2}\;$from dishonest strategies, the values of $G_{1}/G_{2}$ were set at 80, 100,120, and 140. The simulation outcomes are displayed in Figs 18 and 19. Finally, to investigate the influence of loss preference coefficient $L_{P}\;$and $L_{S}$, the values were set at 1.2, 1.7,2.2 and 2.7. The simulation outcomes are displayed in Figs 20 and 21.

#### Scenario 1. The influence of the benefit ${\;R}_{1}\;$ from DDM.

Fig 9 shows how the NFP’s benefit ${\;R}_{1}$ from implementing DDM influences the strategies of the NFP, carrier, and shipper. As revealed by Fig 9, the probability of the NFP choosing DDM (represented by x) increases with the benefits${\;R}_{1}$ from DDM increase. This demonstrates that the greater $R_{1}$, the more likely the NFP is to choose and maintain the DDM strategy. Before x is stable at 1, when${\;R}_{1}$ increases, y and z is decrease and finally stabilizes at 1. However, after x is stable at 1, an increase in ${\;R}_{1}$ does not directly lead to the other two members in the system choosing a more honest strategy more prominently. In reality, only when the NFP shares the revenue will it have a greater impact on their choices.

#### Scenario 2. The influence of subsidies $S_{1}\;$ and$\begin{array}{c} {\;S}_{2} \end{array}$.

Fig 10 shows how the subsidy provided by the NFP to carrier affects the strategies of the NFP, carriers, and shippers. As depicted in Fig 10, once the value of x stables at 1, an increase in${\;S}_{1}$ causes y to increase and eventually stabilize at 1, while z decreases and takes longer to stabilize. The purpose of the subsidy${\;S}_{1}$ is to incentivize carriers to operate honestly and lawfully. By increasing $S_{1},$ carriers are further motivated to avoid dishonest practices, invest more in their operations, and improve service quality and transportation safety. Additionally,${\;S}_{1}\;$benefits shippers by attracting more carriers to the NFP, making the platform more competitive. This provides shippers with greater flexibility in selecting the best transport service provider, potentially reducing their transportation costs.

Fig 11 shows how the subsidy provided by the NFP to shippers affects the strategies of the NFP, carriers, and shippers. According to Fig 11, once x stabilizes at 1, an increase in $S_{2}$ leads to a decrease in y, though it eventually stabilizes at 1. Meanwhile, z increases and stabilizes at 1 more quickly. The results suggest that subsidies encourage shippers to engage in honest and lawful transactions, increasing trust between them and the carriers. This trust makes carriers more willing to cooperate, as they can be more confident that shippers will act ethically and avoid fraud or misconduct.

#### Scenario 3. The influence of penalty $T_{1}$ and $T_{2}$ to dishonest strategy.

Figs 12 and 13 illustrate how the penalty imposed on dishonest behavior by the carrier and shipper affects the evolution of the NFP, carrier, and shipper’s strategies, respectively. According to Fig 12, after x is stable at 1, the influence of $T_{1}$ on y is divided into two parts: when $T_{1}$ is less than a certain value, y is stable at 0, and the smaller $T_{1}$ is, the faster y stabilizes at 0; when $T_{1}$ is greater than a certain value, y is stable at 1, and the larger $T_{1}$, the faster y stabilizes with 1. This shows that if the penalty for the carrier’s dishonest conduct is too small, it cannot play the purpose of guiding the carrier to conduct honest transactions, and only when the penalty $T_{1}$ is large enough to a certain value, it can play the effect of punishment. The same effect of $T_{2}$ on the shipper can been seen. After x is stable at 1, the influence of $T_{2}$ on z is divided into two parts: when $T_{2}$ is less than a certain value, z is stable at 0, and the smaller $T_{2}$ is, the faster z stabilizes at 0; when $T_{2}$ is greater than a certain value, z is stable at 1, and the larger $T_{2}$, the faster z stabilizes with 1. It is evident that higher penalties lead to a reduction in dishonest behavior, as expected. While this relationship may seem intuitive, its theoretical implications and broader applications are worth exploring in more depth.

The negative relationship between penalties and dishonesty can be understood within the framework of EGT, where the evolution of strategies is influenced by the payoffs associated with different actions. Specifically, replicator dynamics in EGT suggest that strategies with higher payoffs, such as honesty, will become more prevalent over time if the associated costs of dishonesty are sufficiently high. In the NFPs, the introduction of higher penalties serves as a regulatory mechanism that alters the payoff structure, tipping the balance in favor of honest strategies. Furthermore, this result is consistent with findings in behavioral economics, which highlight how penalties and deterrence mechanisms influence individual and collective behaviors in real-world settings [41,42].

The broader application of these findings extends to the design of incentive structures in logistics and supply chain platforms. For instance, NFPs can benefit from incorporating tiered penalty systems that are aligned with performance metrics, where higher penalties are imposed for repeated dishonest behavior. This would incentivize transparency and encourage cooperation, thus improving platform performance and efficiency. Additionally, policymakers and platform operators can use these insights to design regulations and incentive schemes that enhance the reliability and sustainability of digital platforms.

#### Scenario 4. The influence of the probability α of the carrier’s or the shipper’s dishonest strategy discovered by the NFP’s choosing DDM.

Fig 14 illustrates how the probability of detecting dishonest behavior by the NFP affects the evolution of the NFP, carrier, and shipper’s strategies. As depicted in Fig 14, once x is stable at 1, the influence of α on y is divided into two parts: when α is less than a certain value, y is stable at 0, and the smaller $T_{1}$ is, the faster y stabilizes at 0; when α is greater than a certain value, y is stable at 1, and the larger α, the faster y stabilizes with 1. This shows that if the probability of the carrier’s dishonest strategy discovered by the NFP’s choosing DDM is too small, the impact of DDM on carriers is limited, and only when the probability α is large enough to a certain value, it can play the effect of DDM. So, the NFP should use DDM to set α within a reasonable range. After the evolution probability of the NFP choosing DDM is stable at 1, when α increases, z increases and finally stabilizes at 1. This is because DDM can better identify and manage the potential risk of dishonesty of the shipper.

#### Scenario 5. The influence of extra costs$C_{i},\;i=1,2,3$.

The effects of three extra costs {$C_{1}$, $C_{2}$, $C_{3}$} corresponding to the strategy {DDM, honesty, honesty} on the evolutionary game of the system are examined by Figs 15–17. According to Fig 15, the influence of different $C_{1}$ on the evolution direction of the three subjects is not significant. According to Fig 16, once the value of x stables at 1, an increase in $C_{2}$ causes y to decrease and take longer to stabilize at 1, while z increases and stabilizes at 1 faster. This is consistent with the reality that if the cost of honest transaction is greater, the carrier is less willing to choose honest strategy. If extra cost for carriers rise, they may be more careful to manage their operations and strictly comply with regulations. This will reduce fraud, and therefore, the carrier’s strategy is stable in choosing honest. The increase in extra costs for the carrier will allow him to make improvements in service quality and safety, which will directly benefit shippers as they will receive more reliable and comprehensive transportation services. So as ${\;C}_{2}$ increases, the shipper stabilizes more quickly in the honest strategy. According to Fig 17, once the value of x stables at 1, an increase in ${\;C}_{3}$ increases, causes y to increase and stabilize at 1 faster, while z decreases and takes longer to stabilize at 1.

#### Scenario 6. The influence of bonuses from dishonest strategies by the carrier/ shipper ($G_{1}/G_{2})$.

Figs 18 and 19 illustrate how the bonuses gained from dishonest behavior by carriers and shippers, respectively, affect the evolution of the NFP, carrier, and shipper strategies. According to Fig 18, once x is stable at 1, an increase in${\;G}_{1}$ leads to a decrease in y, which then gradually stabilizes at 1, while z increases and stabilizes at 1 more quickly. This pattern occurs because the speculative income ${\;G}_{1}$ serves as a key motivator, tempting carriers to engage in dishonest practices despite the risk. As $G_{1}$ rises, the allure of dishonest behavior grows, making carriers more inclined to take risks with fraudulent transactions. In response, shippers anticipate this increase in carrier dishonesty and adjust their behavior. When $G_{1}$ is low, shippers perceive carriers as less likely to act dishonestly, fostering trust and accelerating the adoption of honest strategies by shippers. Similarly, once x is stable at 1, an escalation in${\;G}_{2}$ is associated with an escalation in y, which then stabilizes at 1, while z decreases and takes longer to stabilize at 1. The effect is similar to that of ${\;G}_{1}$. A larger${\;G}_{2}$ increases the temptation for shippers to choose dishonest strategies. In turn, carriers, expecting dishonest responses from shippers due to the high${\;G}_{2},$ may adjust their own choices. When ${\;G}_{2}$ is low, carriers believe shippers are less likely to act dishonestly, making honest transactions more appealing to both parties.

#### Scenario 7. The influence of loss preference coefficient $L_{P}\;$ and$L_{S}$.

Figs 20 and 21 illustrate how loss preference coefficient of the NFP and carrier, respectively, affect the evolution of the NFP, carrier, and shipper strategies. According to Fig 20, the larger the $L_{P}$, the faster x stabilizes at 1. Before x reaches 1, the larger the $L_{P}$, the closer y and z are to 1. The NFP’s loss preference coefficient indicates how sensitive the NFP is to losses incurred from dishonest behavior, inefficient collaboration, or penalties imposed by regulatory frameworks. A higher loss preference coefficient implies that the NFP is more averse to losses, pushing it to adopt data-driven mechanisms (DDMs) more aggressively to mitigate risks. The NFP prioritizes transparency, real-time tracking, and strict penalties to discourage dishonest behaviors by shippers and carriers. A higher loss preference coefficient implies that the NFP is more averse to losses, pushing it to adopt DDM more aggressively to mitigate risks. The NFP prioritizes transparency, real-time tracking, and strict penalties to discourage dishonest behaviors by shippers and carriers. According to Fig 21, the larger the $L_{S}$, the faster z stabilizes at 1. Before z reaches 1, the larger the $L_{s}$, the speed at which x and y approach 1 first increases, and then slowers when z reaches a certain value. A higher loss preference coefficient drives shippers to prioritize reliable carriers and trustworthy NFPs. Shippers will carefully evaluate carriers based on their historical performance and the platform’s ability to enforce fairness through incentives and penalties. Shippers are more likely to adopt honest strategies, such as providing accurate shipment details and paying fair rates, to foster better relationships with carriers and the NFP. This reduces the risk of disputes and operational inefficiencies. Carriers are incentivized to adopt honest behaviors to attract and retain risk-averse shippers. These carriers focus on ensuring timely deliveries, providing accurate tracking information, and avoiding actions (e.g., overbooking or route deviations) that might result in penalties or loss of shipper trust.

**Fig 22:**
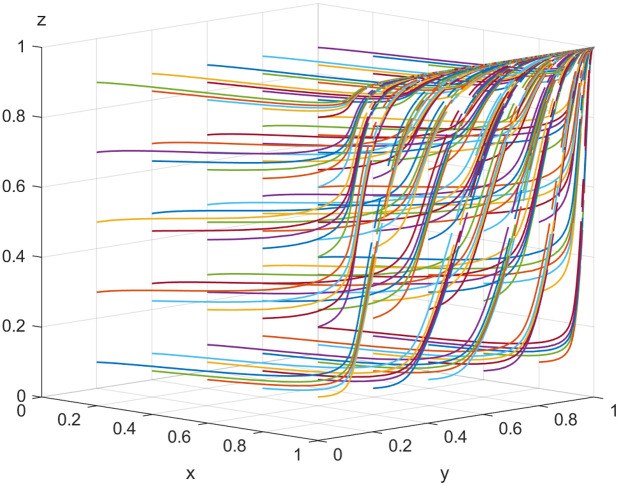
The result of 50 evolutions of array 1.

Next, we will consider that array 1 evolves 50 times at different times with different initial policy combinations. Fig 22 shows the results of evolution.

According to Fig 22, the simulated findings reveal that P_8_ (1,1,1) represents the lone evolutionarily stable strategy triplet, where the NFP adopts DDM, and both the carrier and shipper choose honest strategies. This finding directly supports the research question regarding how DDM promote cooperative behavior. The alignment with the asymptotic stability analysis reinforces the robustness of this outcome, demonstrating that adopting DDM leads to greater overall stability in the supply chain.

The simulation analysis demonstrates a high level of consistency, aligning with scholarly standards. It is effective with the conclusion of the strategic stability analysis of all parties, and has practical guiding significance for digital transformation of the NFP.

## Conclusions

This research was motivated by the growing complexity of stakeholder interactions within network freight platforms (NFPs) and the critical need to foster cooperation between shippers, carriers, and platform operators. This study set out to delve into how data-driven mechanisms, including real-time feedback, might be utilized to strengthen cooperation, reduce opportunistic behaviors, and improve overall supply chain efficiency. Using evolutionary game theory (EGT), this study modeled the dynamic interactions between shippers, carriers, and platform operators, highlighting the influence of strategies such as subsidies and penalties on promoting cooperative behavior and stable engagements across the supply chain. While the results suggest that these strategies play a significant role in fostering cooperation, it is important to note that the model primarily focuses on subsidies and penalties. A more comprehensive investigation, incorporating a wider range of strategies, is necessary to definitively confirm their status as key drivers of supply chain cooperation. Future studies could explore additional strategies, such as pricing mechanisms, contract design, or information sharing, to provide a more holistic understanding of the factors influencing stakeholder behavior in network freight platforms.

The study’s findings offer actionable insights for network freight platforms. For example, implementing penalties proportional to the extent of dishonesty, similar to industry practices, discourages opportunistic behavior. Real-world detection rates achieved through technologies like blockchain and IoT suggest that increasing the probability of identifying dishonest actions can effectively deter such behaviors. Our model provides a strategic framework for designing incentive systems that reflect these practical realities, fostering long-term cooperation among shippers, carriers, and platforms.

The findings of the study confirm that data-driven mechanisms play a key role in fostering cooperation. Specifically, the study answers the research question regarding how incentives (such as subsidies and penalties) drive more stable and honest engagements across the supply chain. By promoting transparency and accountability, these mechanisms enhance trust among stakeholders, which leads to more efficient logistics operations. For practitioners, this means developing structured incentive programs that reward collaborative actions and impose costs on opportunistic behaviors. Tailoring these financial mechanisms to specific operational contexts can lead to enhanced cooperation. The confirmation of this hypothesis advances the theoretical understanding of incentive design within NFPs, offering new insights into their practical implementation.

One of the core contributions of this research lies in its novel integration of data analytics with evolutionary game theory, an area that has been less explored in the context of digital logistics platforms. While prior studies have applied evolutionary game theory to model stakeholder behavior, they typically neglect the impact of real-time data feedback and adaptive strategies on cooperation. In contrast, this study introduces data-driven mechanisms that provide continuous updates on stakeholder performance and adjust incentives accordingly, creating a dynamic framework for decision-making. This approach not only extends the theoretical understanding of cooperation in digital logistics platforms but also empirically confirms the hypothesis that real-time, data-driven feedback can stabilize long-term cooperation among stakeholders. The findings emphasize the significance of key initial conditions, such as the detection probability of dishonest behaviors and the structuring of financial incentives, in shaping the evolution of cooperative strategies. This highlights the importance of designing balanced incentive systems to foster sustainable collaboration. In doing so, the research makes a direct contribution to the literature on cooperative dynamics by addressing gaps in previous models that overlook adaptive, data-driven elements.

### Limitations and future research

Despite the contributions of this study, several limitations should be considered when interpreting the results. Firstly, the model assumes rational decision-making by all stakeholders, meaning that shippers, carriers, and the platform are assumed to act in a way that maximizes their individual payoffs based on available information. While this assumption is common in game-theoretic models, it may not fully capture the complexity of decision-making in real-world logistics platforms, where bounded rationality and imperfect information are often prevalent. Future research could explore more realistic behavioral models that incorporate cognitive biases or incomplete information, which could alter the dynamics of cooperation and competition.

Second, the model assumes a static structure for the market and financial incentives, without accounting for potential market-specific conditions or external factors such as economic shocks or regulatory changes. In practice, logistics markets are dynamic, and stakeholder decisions may be influenced by a variety of evolving factors, including changes in supply and demand, regulatory shifts, and technological advancements. The impact of these market-specific conditions could significantly affect the robustness of the findings, as the model does not capture such complexities. Future work could expand the model to incorporate these dynamic factors and test the model’s robustness under varying market conditions.

Finally, the study’s focus on a simplified version of cooperative behavior may limit its applicability to more complex scenarios where additional factors, such as long-term strategic planning or relationships among stakeholders, play a role. Expanding the scope of the model to include such factors could enhance its generalizability and provide a more comprehensive understanding of cooperation in digital logistics platforms.

This research offers a comprehensive framework for understanding cooperation dynamics within NFPs and provides actionable insights for leveraging data-driven mechanisms to optimize stakeholder interactions. This scholarly work strengthens the theoretical foundation and practical application in transportation management and supply chain logistics by diligently relating its findings to the initial research inquiries. The real-world implications of our findings are significant and can be translated into several strategic recommendations for industry practitioners and policymakers.

For industry practitioners, the integration of real-time feedback and adaptive strategies into their operations can be transformative. The following recommendations can be made based on our findings: (1) Implement structured incentive programs that clearly define the rewards for cooperative behavior and the penalties for opportunistic actions. These programs should be designed to align with the specific operational contexts and should be regularly reviewed and adjusted to ensure their effectiveness. (2) Invest in advanced data analytics capabilities to monitor and provide real-time feedback on stakeholder behaviors. This will enable NFPs to quickly identify and address instances of non-cooperation, thereby maintaining a culture of transparency and trust. (3) Develop detection systems that accurately identify dishonest strategies, which is crucial for the success of incentive mechanisms. Operators could explore the use of blockchain technology to create secure and transparent records of transactions.

For policymakers, the study suggests the following actions: (1) Create regulatory frameworks that support the adoption of data-driven mechanisms within NFPs. This includes ensuring that subsidies and penalties are used in a manner that promotes fair competition and honest behavior without imposing unnecessary burdens on the industry. (2) Foster the advancement and adoption of technologies that enhance transparency and accountability in logistics operations. (3) Support research and development in behavioral economics as it relates to supply chain management, to better understand and predict stakeholder behavior in the face of various incentives and sanctions.

## Supporting information

S1 File Programs. (ZIP)
