# Supplementary material for: Data-driven mechanisms for network freight platforms: An evolutionary game perspective
Source: PLoS One. 2025 Jun 27;20(6):e0319842. doi: 10.1371/journal.pone.0319842 (PMC12204633; doi:10.1371/journal.pone.0319842)
Supplement: S1 File — (ZIP) [file pone.0319842.s001.zip › Programs/Calculation program.docx]

1. **Find the dynamic equation of replication**

clc; clear;

syms x y z a b R1 R2 R3 W F1 F2 F3 S1 S2 C1 C12 C2 T1 T2 H1 H2 LP

% **Calculate F(x)**

E11=y*z*(F1+R1+W-LP*C1-LP*S1-LP*S2)+y*(1-z)*(F1+R1+W-LP*C1-LP*S1-(1-a)*LP*S2+a*T2-a*LP*H2)+(1-y)*z*(F1+R1+W-LP*C1-(1-a)*LP*S1-LP*S2+a*T1-a*LP*H1)+(1-y)*(1-z)*(F1+R1+W-LP*C1+a*T1+a*T2-(1-a)*LP*S1-(1-a)*LP*S2-a*LP*H1-a*LP*H2);%¼Ó´Ö±äÁ¿×ÔÐÐ¸ü¸Ä

x1=simplify(E11)

E12=y*z*(F1-LP*S1-LP*S2)+y*(1-z)*(F1-LP*S1-(1-b)*LP*S2+b*T2-b*LP*H2)+(1-y)*z*(F1-(1-b)*LP*S1-LP*S2+b*T1-b*LP*H1)+(1-y)*(1-z)*(F1+b*T1+b*T2-(1-b)*LP*S1-(1-b)*LP*S2-b*LP*H1-b*LP*H2);

x2=simplify(E12)

E1=simplify(x*E11+(1-x)*E12)

F(x)=simplify(x*(E11-E1))

%%%%%%%%%%%%%%%%%%%%%%%%%%%%%%%%%%

% **Calculate F(y)**

clc; clear;

syms x y z a b R1 R2 R3 W F1 F2 F3 S1 S2 C1 C2 T1 T2 K1 K2 G1 G2 H1 H2

E21=x*z*(F2+R2+S1-C2)+x*(1-z)*(F2+R2+S1-C2-K2+a*H2)+(1-x)*z*(F2+S1-C2)+(1-x)*(1-z)*(F2+S1-C2-K2+b*H2);%¼Ó´Ö±äÁ¿×ÔÐÐ¸ü¸Ä

y1=simplify(E21)

E22=x*z*(F2+R2+(1-a)*S1+G1-a*T1)+x*(1-z)*(F2+R2+G1-K2-a*T1+a*H2)+(1-x)*z*(F2+G1-b*T1+(1-b)*S1)+(1-x)*(1-z)*(F2+G1+(1-b)*S1-b*T1-K2+b*H2);%¼Ó´Ö±äÁ¿×ÔÐÐ¸ü¸Ä

y1=simplify(E22)

E2=simplify(y*E21+(1-y)*E22)%ÇóÆ½¾ùÆÚÍû

F(y)=simplify(y*(E21-E2))

%%%%%%%%%%%%%%%%%%%%%%%%%%%%%%%%%%

% **Calculate F(z)**

clc;clear;

syms x y z a b R1 R2 R3 W F1 F2 F3 S1 S2 C1 C2 C3 T1 T2 K1 K2 G1 G2 H1 H2 LC

E31=x*y*(F3+R3+S2-LC*C3)+x*(1-y)*(F3+R3+S2-LC*C3-LC*K1+a*H1)+(1-x)*y*(F3+S2-LC*C3)+(1-x)*(1-y)*(F3+S2-LC*C3-LC*K1+b*H1);%¼Ó´Ö±äÁ¿×ÔÐÐ¸ü¸Ä

E32=x*y*(F3+R3+G2+(1-a)*S2-a*LC*T2)+x*(1-y)*(F3+R3+G2-a*LC*T2-LC*K1+a*H1)+(1-x)*y*(F3+G2+(1-b)*S2-b*LC*T2)+(1-x)*(1-y)*(F3+G2-LC*K1-b*LC*T2+b*H1);%¼Ó´Ö±äÁ¿×ÔÐÐ¸ü¸Ä

E3=simplify(z*E31+(1-z)*E32)%ÇóÆ½¾ùÆÚÍû

F(z)=simplify(z*(E31-E3))

%%%%%%%%%%%%%%%%%%%%%%%%%%%%%%%%%%%%%%%

%%%%%%%%%%%%%%%%%%%%%%%%%%%%%%%%%%%%%%%%

%**- Solve the Jacobian matrix eigenvalues**

% **Equilibrium point E1**

clc;clear;

syms x y z a b R1 R2 R3 W F1 F2 F3 S1 S2 C1 C2 C3 T1 T2 K1 K2 G1 G2 H1 H2 LP LS;

x=0,y=0,z=0;

A = [-(2*x-1)*(R1-LP*C1+W-LP*H1*a-LP*H2*a+LP*H1*b+LP*H2*b+LP*S1*a+LP*S2*a-LP*S1*b+T1*a-LP*S2*b+T2*a-T1*b-T2*b+LP*H1*a*y-LP*H1*b*y+LP*H2*a*z-LP*H2*b*z-LP*S1*a*y+LP*S1*b*y-T1*a*y-LP*S2*a*z+T1*b*y+LP*S2*b*z-T2*a*z+T2*b*z) x*(a-b)*(x-1)*(LP*S1-LP*H1+T1) x*(a-b)*(x-1)*(LP*S2-LP*H2+T2);

-y*(y-1)*(S1-S1*b+T1*a-T1*b-S1*z+S1*a*z) (2*y-1)*(C2+G1-S1*b-T1*b-S1*x+S1*b*x-T1*a*x+T1*b*x+S1*x*z-S1*a*x*z) -S1*x*y*(a-1)*(y-1);

-z*(a-b)*(z-1)*(LS*T2+S2*y) -S2*z*(z-1)*(b+a*x-b*x-1) (2*z-1)*(LS*C3+G2 - S2 - LS*T2*b+S2*y-LS*T2*a*x-S2*b*y+LS*T2*b*x-S2*a*x*y+S2*b*x*y)];

[V,R] = eig(A),DA=det(A),IA=inv(A),

% **Equilibrium point E2**

clc;clear;

syms x y z a b R1 R2 R3 W F1 F2 F3 S1 S2 C1 C2 C3 T1 T2 K1 K2 G1 G2 H1 H2 LP LS;

x=1,y=0,z=0;

A = [-(2*x-1)*(R1-LP*C1+W-LP*H1*a-LP*H2*a+LP*H1*b+LP*H2*b+LP*S1*a+LP*S2*a-LP*S1*b+T1*a-LP*S2*b+T2*a-T1*b-T2*b+LP*H1*a*y-LP*H1*b*y+LP*H2*a*z-LP*H2*b*z-LP*S1*a*y+LP*S1*b*y-T1*a*y-LP*S2*a*z+T1*b*y+LP*S2*b*z-T2*a*z+T2*b*z) x*(a-b)*(x-1)*(LP*S1-LP*H1+T1) x*(a-b)*(x-1)*(LP*S2-LP*H2+T2);

-y*(y-1)*(S1-S1*b+T1*a-T1*b-S1*z+S1*a*z) (2*y-1)*(C2+G1-S1*b-T1*b-S1*x+S1*b*x-T1*a*x+T1*b*x+S1*x*z-S1*a*x*z) -S1*x*y*(a-1)*(y-1);

-z*(a-b)*(z-1)*(LS*T2+S2*y) -S2*z*(z-1)*(b+a*x-b*x-1) (2*z-1)*(LS*C3+G2 - S2 - LS*T2*b+S2*y-LS*T2*a*x-S2*b*y+LS*T2*b*x-S2*a*x*y+S2*b*x*y)];

[V,R] = eig(A),DA=det(A),IA=inv(A),

% **Equilibrium point E3**

clc;clear;

syms x y z a b R1 R2 R3 W F1 F2 F3 S1 S2 C1 C2 C3 T1 T2 K1 K2 G1 G2 H1 H2 LP LS;

x=0,y=1,z=0; %¾ùºâµã

A = [-(2*x-1)*(R1-LP*C1+W-LP*H1*a-LP*H2*a+LP*H1*b+LP*H2*b+LP*S1*a+LP*S2*a-LP*S1*b+T1*a-LP*S2*b+T2*a-T1*b-T2*b+LP*H1*a*y-LP*H1*b*y+LP*H2*a*z-LP*H2*b*z-LP*S1*a*y+LP*S1*b*y-T1*a*y-LP*S2*a*z+T1*b*y+LP*S2*b*z-T2*a*z+T2*b*z) x*(a-b)*(x-1)*(LP*S1-LP*H1+T1) x*(a-b)*(x-1)*(LP*S2-LP*H2+T2);

-y*(y-1)*(S1-S1*b+T1*a-T1*b-S1*z+S1*a*z) (2*y-1)*(C2+G1-S1*b-T1*b-S1*x+S1*b*x-T1*a*x+T1*b*x+S1*x*z-S1*a*x*z) -S1*x*y*(a-1)*(y-1);

-z*(a-b)*(z-1)*(LS*T2+S2*y) -S2*z*(z-1)*(b+a*x-b*x-1) (2*z-1)*(LS*C3+G2 - S2 - LS*T2*b+S2*y-LS*T2*a*x-S2*b*y+LS*T2*b*x-S2*a*x*y+S2*b*x*y)];

[V,R] = eig(A),DA=det(A),IA=inv(A),

% **Equilibrium point E4**

clc;clear;

syms x y z a b R1 R2 R3 W F1 F2 F3 S1 S2 C1 C2 C3 T1 T2 K1 K2 G1 G2 H1 H2 LP LS;

x=0,y=0,z=1; %¾ùºâµã

A = [-(2*x-1)*(R1-LP*C1+W-LP*H1*a-LP*H2*a+LP*H1*b+LP*H2*b+LP*S1*a+LP*S2*a-LP*S1*b+T1*a-LP*S2*b+T2*a-T1*b-T2*b+LP*H1*a*y-LP*H1*b*y+LP*H2*a*z-LP*H2*b*z-LP*S1*a*y+LP*S1*b*y-T1*a*y-LP*S2*a*z+T1*b*y+LP*S2*b*z-T2*a*z+T2*b*z) x*(a-b)*(x-1)*(LP*S1-LP*H1+T1) x*(a-b)*(x-1)*(LP*S2-LP*H2+T2);

-y*(y-1)*(S1-S1*b+T1*a-T1*b-S1*z+S1*a*z) (2*y-1)*(C2+G1-S1*b-T1*b-S1*x+S1*b*x-T1*a*x+T1*b*x+S1*x*z-S1*a*x*z) -S1*x*y*(a-1)*(y-1);

-z*(a-b)*(z-1)*(LS*T2+S2*y) -S2*z*(z-1)*(b+a*x-b*x-1) (2*z-1)*(LS*C3+G2 - S2 - LS*T2*b+S2*y-LS*T2*a*x-S2*b*y+LS*T2*b*x-S2*a*x*y+S2*b*x*y)];

[V,R] = eig(A),DA=det(A),IA=inv(A),

% **Equilibrium point E5**

clc;clear;

syms x y z a b R1 R2 R3 W F1 F2 F3 S1 S2 C1 C2 C3 T1 T2 K1 K2 G1 G2 H1 H2 LP LS;

x=1,y=1,z=0; %¾ùºâµã

A = [-(2*x-1)*(R1-LP*C1+W-LP*H1*a-LP*H2*a+LP*H1*b+LP*H2*b+LP*S1*a+LP*S2*a-LP*S1*b+T1*a-LP*S2*b+T2*a-T1*b-T2*b+LP*H1*a*y-LP*H1*b*y+LP*H2*a*z-LP*H2*b*z-LP*S1*a*y+LP*S1*b*y-T1*a*y-LP*S2*a*z+T1*b*y+LP*S2*b*z-T2*a*z+T2*b*z) x*(a-b)*(x-1)*(LP*S1-LP*H1+T1) x*(a-b)*(x-1)*(LP*S2-LP*H2+T2);

-y*(y-1)*(S1-S1*b+T1*a-T1*b-S1*z+S1*a*z) (2*y-1)*(C2+G1-S1*b-T1*b-S1*x+S1*b*x-T1*a*x+T1*b*x+S1*x*z-S1*a*x*z) -S1*x*y*(a-1)*(y-1);

-z*(a-b)*(z-1)*(LS*T2+S2*y) -S2*z*(z-1)*(b+a*x-b*x-1) (2*z-1)*(LS*C3+G2 - S2 - LS*T2*b+S2*y-LS*T2*a*x-S2*b*y+LS*T2*b*x-S2*a*x*y+S2*b*x*y)];

[V,R] = eig(A),DA=det(A),IA=inv(A),

% **Equilibrium point E6**

clc;clear;

syms x y z a b R1 R2 R3 W F1 F2 F3 S1 S2 C1 C2 C3 T1 T2 K1 K2 G1 G2 H1 H2 LP LS;

x=1,y=0,z=1; %¾ùºâµã

A = [-(2*x-1)*(R1-LP*C1+W-LP*H1*a-LP*H2*a+LP*H1*b+LP*H2*b+LP*S1*a+LP*S2*a-LP*S1*b+T1*a-LP*S2*b+T2*a-T1*b-T2*b+LP*H1*a*y-LP*H1*b*y+LP*H2*a*z-LP*H2*b*z-LP*S1*a*y+LP*S1*b*y-T1*a*y-LP*S2*a*z+T1*b*y+LP*S2*b*z-T2*a*z+T2*b*z) x*(a-b)*(x-1)*(LP*S1-LP*H1+T1) x*(a-b)*(x-1)*(LP*S2-LP*H2+T2);

-y*(y-1)*(S1-S1*b+T1*a-T1*b-S1*z+S1*a*z) (2*y-1)*(C2+G1-S1*b-T1*b-S1*x+S1*b*x-T1*a*x+T1*b*x+S1*x*z-S1*a*x*z) -S1*x*y*(a-1)*(y-1);

-z*(a-b)*(z-1)*(LS*T2+S2*y) -S2*z*(z-1)*(b+a*x-b*x-1) (2*z-1)*(LS*C3+G2 - S2 - LS*T2*b+S2*y-LS*T2*a*x-S2*b*y+LS*T2*b*x-S2*a*x*y+S2*b*x*y)];

[V,R] = eig(A),DA=det(A),IA=inv(A),

% **Equilibrium point E7**

clc;clear;

syms x y z a b R1 R2 R3 W F1 F2 F3 S1 S2 C1 C2 C3 T1 T2 K1 K2 G1 G2 H1 H2 LP LS;

x=0,y=1,z=1; %¾ùºâµã

A = [-(2*x-1)*(R1-LP*C1+W-LP*H1*a-LP*H2*a+LP*H1*b+LP*H2*b+LP*S1*a+LP*S2*a-LP*S1*b+T1*a-LP*S2*b+T2*a-T1*b-T2*b+LP*H1*a*y-LP*H1*b*y+LP*H2*a*z-LP*H2*b*z-LP*S1*a*y+LP*S1*b*y-T1*a*y-LP*S2*a*z+T1*b*y+LP*S2*b*z-T2*a*z+T2*b*z) x*(a-b)*(x-1)*(LP*S1-LP*H1+T1) x*(a-b)*(x-1)*(LP*S2-LP*H2+T2);

-y*(y-1)*(S1-S1*b+T1*a-T1*b-S1*z+S1*a*z) (2*y-1)*(C2+G1-S1*b-T1*b-S1*x+S1*b*x-T1*a*x+T1*b*x+S1*x*z-S1*a*x*z) -S1*x*y*(a-1)*(y-1);

-z*(a-b)*(z-1)*(LS*T2+S2*y) -S2*z*(z-1)*(b+a*x-b*x-1) (2*z-1)*(LS*C3+G2 - S2 - LS*T2*b+S2*y-LS*T2*a*x-S2*b*y+LS*T2*b*x-S2*a*x*y+S2*b*x*y)];

[V,R] = eig(A),DA=det(A),IA=inv(A),

% **Equilibrium point E8**

clc;clear;

syms x y z a b R1 R2 R3 W F1 F2 F3 S1 S2 C1 C2 C3 T1 T2 K1 K2 G1 G2 H1 H2 LP LS;

x=1,y=1,z=1; %¾ùºâµã

A = [-(2*x-1)*(R1-LP*C1+W-LP*H1*a-LP*H2*a+LP*H1*b+LP*H2*b+LP*S1*a+LP*S2*a-LP*S1*b+T1*a-LP*S2*b+T2*a-T1*b-T2*b+LP*H1*a*y-LP*H1*b*y+LP*H2*a*z-LP*H2*b*z-LP*S1*a*y+LP*S1*b*y-T1*a*y-LP*S2*a*z+T1*b*y+LP*S2*b*z-T2*a*z+T2*b*z) x*(a-b)*(x-1)*(LP*S1-LP*H1+T1) x*(a-b)*(x-1)*(LP*S2-LP*H2+T2);

-y*(y-1)*(S1-S1*b+T1*a-T1*b-S1*z+S1*a*z) (2*y-1)*(C2+G1-S1*b-T1*b-S1*x+S1*b*x-T1*a*x+T1*b*x+S1*x*z-S1*a*x*z) -S1*x*y*(a-1)*(y-1);

-z*(a-b)*(z-1)*(LS*T2+S2*y) -S2*z*(z-1)*(b+a*x-b*x-1) (2*z-1)*(LS*C3+G2 - S2 - LS*T2*b+S2*y-LS*T2*a*x-S2*b*y+LS*T2*b*x-S2*a*x*y+S2*b*x*y)];

[V,R] = eig(A),DA=det(A),IA=inv(A),
