# Supplementary material for: Data-driven mechanisms for network freight platforms: An evolutionary game perspective
Source: PLoS One. 2025 Jun 27;20(6):e0319842. doi: 10.1371/journal.pone.0319842 (PMC12204633; doi:10.1371/journal.pone.0319842)
Supplement: S1 File — (ZIP) [file pone.0319842.s001.zip › Programs/Fig5-Fig8.docx]

1. **Fig 5. Effect of Initial Setting.**

clc,clear;

figure(2);

a=0.85, b=0.68, R1=800, W=80,C1=180,S1=100,S2=70,H1=240,H2=300,T1=300,T2=350, C2=120, C3=100,G1=180, G2=140, LP=1.2,LS=1.6;

% V1=a*S2-C3-G2+a*T2

% V2=a*S1-C2-G1+a*T1

% V3=R1+W-C1

[t,y]=ode45(@(t,y) Datefunction(t,y,a,b,R1,W,S1,S2,C1,C2,C3,T1,T2,G1,G2,H1,H2,LP,LS),[0 1],[0.5 0.5 0.5]);

points=1:2:length(t);

plot(y(:,1),'rh-','linewidth',1,'markersize',5,'markerfacecolor','r','markerindices',points);

hold on;

[t,y]=ode45(@(t,y) Datefunction(t,y,a,b,R1,W,S1,S2,C1,C2,C3,T1,T2,G1,G2,H1,H2,LP,LS),[0 1],[0.5 0.5 0.5]);

points=1:2:length(t);

plot(y(:,2),'g--','linewidth',1,'markersize',5,'markerindices',points);

hold on;

[t,y]=ode45(@(t,y) Datefunction(t,y,a,b,R1,W,S1,S2,C1,C2,C3,T1,T2,G1,G2,H1,H2,LP,LS),[0 1],[0.5 0.5 0.5]);

points=1:2:length(t);

plot(y(:,3),'b:','linewidth',1,'markersize',5,'markerindices',points);

hold on;

set(0,'defaultfigurecolor','w')

grid on

hold on

xlabel('$Time$','interpreter','latex','Rotation',0.2);

ylabel('$Proportion$','interpreter','latex');

set(gca,'XTick',[0:20:150],'YTick',[0.4:0.1:1.1])

axis([0 150 0.4 1.1]);

**%%%%%%%%%%%%%%%%%%%%%%%%%%%%%%%%%%%%%%%%%%%%%%%%%%%%%%**

1. **Fig 6. The impact of initial probability of the NFP on the carrier and shipper**

clc,clear;

figure(3);

subplot(2,2,1);

a=0.85, b=0.68, R1=800, W=80,C1=180,S1=100,S2=70,H1=240,H2=300,T1=300,T2=350, C2=120, C3=100,G1=180, G2=140, LP=1.2,LS=1.6;

[t,y]=ode45(@(t,y) Datefunction(t,y,a,b,R1,W,S1,S2,C1,C2,C3,T1,T2,G1,G2,H1,H2,LP,LS),[0 1],[0.01 0.5 0.5]);

points=1:2:length(t);

plot(t,y(:,2),'rh-','linewidth',1,'markersize',5,'markerindices',points);

hold on;

[t,y]=ode45(@(t,y) Datefunction(t,y,a,b,R1,W,S1,S2,C1,C2,C3,T1,T2,G1,G2,H1,H2,LP,LS),[0 1],[0.15 0.5 0.5]);

points=1:2:length(t);

plot(t,y(:,2),'b:','linewidth',1,'markersize',5,'markerindices',points);

hold on;

[t,y]=ode45(@(t,y) Datefunction(t,y,a,b,R1,W,S1,S2,C1,C2,C3,T1,T2,G1,G2,H1,H2,LP,LS),[0 1],[0.55 0.5 0.5]);

points=1:1:length(t);

plot(t,y(:,2),'g:.','linewidth',1,'markersize',5,'markerindices',points);

hold on;

[t,y]=ode45(@(t,y) Datefunction(t,y,a,b,R1,W,S1,S2,C1,C2,C3,T1,T2,G1,G2,H1,H2,LP,LS),[0 1],[0.95 0.5 0.5]);

points=1:2:length(t);

plot(t,y(:,2),'m--','linewidth',1,'markersize',5,'markerindices',points);

hold on;

%白底，网格

set(0,'defaultfigurecolor','w')

grid on

hold on

xlabel('$t$','interpreter','latex','Rotation',0);

ylabel('$y$','interpreter','latex');

set(gca,'XTick',[0:0.03:0.15],'YTick',[0:0.1:1])

axis([0 0.15 0.4 1]);

legend('x=0.01','x=0.15','x=0.55','x=0.95');

subplot(2,2,2);

a=0.85, b=0.68, R1=800, W=80,C1=180,S1=100,S2=70,H1=240,H2=300,T1=300,T2=350, C2=120, C3=100,G1=180, G2=140;

[t,y]=ode45(@(t,y) Datefunction(t,y,a,b,R1,W,S1,S2,C1,C2,C3,T1,T2,G1,G2,H1,H2,LP,LS),[0 1],[0.01 0.5 0.5]);

points=1:2:length(t);

plot(t,y(:,3),'rh:','linewidth',1,'markersize',5,'markerindices',points);

hold on;

[t,y]=ode45(@(t,y) Datefunction(t,y,a,b,R1,W,S1,S2,C1,C2,C3,T1,T2,G1,G2,H1,H2,LP,LS),[0 1],[0.15 0.5 0.5]);

points=1:2:length(t);

plot(t,y(:,3),'b:','linewidth',1,'markersize',5,'markerindices',points);

hold on;

[t,y]=ode45(@(t,y) Datefunction(t,y,a,b,R1,W,S1,S2,C1,C2,C3,T1,T2,G1,G2,H1,H2,LP,LS),[0 1],[0.95 0.5 0.5]);

points=1:2:length(t);

plot(t,y(:,3),'g:.','linewidth',1,'markersize',5,'markerindices',points);

hold on;

[t,y]=ode45(@(t,y) Datefunction(t,y,a,b,R1,W,S1,S2,C1,C2,C3,T1,T2,G1,G2,H1,H2,LP,LS),[0 1],[0.95 0.5 0.5]);

points=1:1:length(t);

plot(t,y(:,3),'m--','linewidth',1,'markersize',5,'markerindices',points);

hold on;

set(0,'defaultfigurecolor','w')

grid on

hold on

xlabel('$t$','interpreter','latex','Rotation',0);

ylabel('$z$','interpreter','latex');

set(gca,'XTick',[0:0.01:0.05],'YTick',[0.4:0.2:1])

axis([0 0.05 0.4 1]);

legend('x=0.01','x=0.15','x=0.55','x=0.95');

% title('Fig.6.Effect of initial probability of manufacturers on recyclers','position',[-0.1 -0.3]','FontWeight','bold');

%%%%%%%%%%%%%%%%%%%%%%%%%%%%%%%%%%%%%%%%%%%%%%%%%%%%%%

**3 Fig 7. The impact of initial probability of the carrier on the NFP and the shipper**

clc,clear;

figure(4);

subplot(2,2,1);

%ÏßÌõ

a=0.85, b=0.68, R1=800, W=80,C1=180,S1=100,S2=70,H1=240,H2=300,T1=300,T2=350, C2=120, C3=100,G1=180, G2=140, LP=1.2,LS=1.6;

[t,y]=ode45(@(t,y) Datefunction(t,y,a,b,R1,W,S1,S2,C1,C2,C3,T1,T2,G1,G2,H1,H2,LP,LS),[0 1],[0.5 0.01 0.5]);

points=1:2:length(t); %ÖÐ¼äµÄ1¸ÄÎª´óÒ»µãµÄÕýÕûÊý£¬ÄÇÃ´Í¼ÏóÉÏµÄµã¾Í»á±äµÃÏ¡Êè

plot(t,y(:,1),'rh-','linewidth',1,'markersize',5,'markerindices',points);

hold on;

[t,y]=ode45(@(t,y) Datefunction(t,y,a,b,R1,W,S1,S2,C1,C2,C3,T1,T2,G1,G2,H1,H2,LP,LS),[0 1],[0.5 0.15 0.5]);

points=1:2:length(t);

plot(t,y(:,1),'b:','linewidth',1,'markersize',5,'markerindices',points);

hold on;

[t,y]=ode45(@(t,y) Datefunction(t,y,a,b,R1,W,S1,S2,C1,C2,C3,T1,T2,G1,G2,H1,H2,LP,LS),[0 1],[0.5 0.55 0.5]);

points=1:1:length(t);

plot(t,y(:,1),'g:.','linewidth',1,'markersize',5,'markerindices',points);

hold on;

[t,y]=ode45(@(t,y) Datefunction(t,y,a,b,R1,W,S1,S2,C1,C2,C3,T1,T2,G1,G2,H1,H2,LP,LS),[0 1],[0.5 0.95 0.5]);

points=1:2:length(t);

plot(t,y(:,1),'m--','linewidth',1,'markersize',5,'markerindices',points);

hold on;

%°×µ×£¬Íø¸ñ

set(0,'defaultfigurecolor','w')

grid on

hold on

%×ø±ê±ê×¢£¬¿Ì¶È¼ä¸ô¼°Çø¼ä

xlabel('$t$','interpreter','latex','Rotation',0);

ylabel('$x$','interpreter','latex');

set(gca,'XTick',[0:0.005:0.015],'YTick',[0.4:0.1:1])

axis([0 0.015 0.4 1]);

legend('y=0.01','y=0.15','y=0.55','y=0.95');

subplot(2,2,2);

%ÏßÌõ

a=0.85, b=0.68, R1=800, W=80,C1=180,S1=100,S2=70,H1=240,H2=300,T1=300,T2=350, C2=120, C3=100,G1=180, G2=140, LP=1.2,LS=1.6;

[t,y]=ode45(@(t,y) Datefunction(t,y,a,b,R1,W,S1,S2,C1,C2,C3,T1,T2,G1,G2,H1,H2,LP,LS),[0 1],[0.5 0.01 0.5]);

points=1:2:length(t); %ÖÐ¼äµÄ1¸ÄÎª´óÒ»µãµÄÕýÕûÊý£¬ÄÇÃ´Í¼ÏóÉÏµÄµã¾Í»á±äµÃÏ¡Êè

plot(t,y(:,3),'rh:','linewidth',1,'markersize',5,'markerindices',points);

hold on;

[t,y]=ode45(@(t,y) Datefunction(t,y,a,b,R1,W,S1,S2,C1,C2,C3,T1,T2,G1,G2,H1,H2,LP,LS),[0 1],[0.5 0.15 0.5]);

points=1:2:length(t);

plot(t,y(:,3),'b:','linewidth',1,'markersize',5,'markerindices',points);

hold on;

[t,y]=ode45(@(t,y) Datefunction(t,y,a,b,R1,W,S1,S2,C1,C2,C3,T1,T2,G1,G2,H1,H2,LP,LS),[0 1],[0.5 0.55 0.5]);

points=1:2:length(t);

plot(t,y(:,3),'g:.','linewidth',1,'markersize',5,'markerindices',points);

hold on;

[t,y]=ode45(@(t,y) Datefunction(t,y,a,b,R1,W,S1,S2,C1,C2,C3,T1,T2,G1,G2,H1,H2,LP,LS),[0 1],[0.5 0.95 0.5]);

points=1:1:length(t);

plot(t,y(:,3),'m--','linewidth',1,'markersize',5,'markerindices',points);

hold on;

%°×µ×£¬Íø¸ñ

set(0,'defaultfigurecolor','w')

grid on

hold on

%×ø±ê±ê×¢£¬¿Ì¶È¼ä¸ô¼°Çø¼ä

xlabel('$t$','interpreter','latex','Rotation',0);

ylabel('$z$','interpreter','latex');

set(gca,'XTick',[0:0.05:0.35],'YTick',[0.4:0.1:1])

axis([0 0.35 0.4 1]);

%%%%%%%%%%%%%%%%%%%%%%%%%%%%%%%%%%%%%%%%%%%%%%%%%%%%%%

1. **Fig 8. The impact of initial probability of the shipper on the NFP and the carrier**

clc,clear;

figure(5);

subplot(2,2,1);

a=0.85, b=0.68, R1=800, W=80,C1=180,S1=100,S2=70,H1=240,H2=300,T1=300,T2=350, C2=120, C3=100,G1=180, G2=140, LP=1.2,LS=1.6;

[t,y]=ode45(@(t,y) Datefunction(t,y,a,b,R1,W,S1,S2,C1,C2,C3,T1,T2,G1,G2,H1,H2,LP,LS),[0 1],[0.5 0.5 0.01]);

points=1:2:length(t);

plot(t,y(:,1),'rh-','linewidth',1,'markersize',5,'markerindices',points);

hold on;

[t,y]=ode45(@(t,y) Datefunction(t,y,a,b,R1,W,S1,S2,C1,C2,C3,T1,T2,G1,G2,H1,H2,LP,LS),[0 1],[0.5 0.5 0.15]);

points=1:2:length(t);

plot(t,y(:,1),'b:','linewidth',1,'markersize',5,'markerindices',points);

hold on;

[t,y]=ode45(@(t,y) Datefunction(t,y,a,b,R1,W,S1,S2,C1,C2,C3,T1,T2,G1,G2,H1,H2,LP,LS),[0 1],[0.5 0.5 0.55]);

points=1:1:length(t);

plot(t,y(:,1),'g:.','linewidth',1,'markersize',5,'markerindices',points);

hold on;

[t,y]=ode45(@(t,y) Datefunction(t,y,a,b,R1,W,S1,S2,C1,C2,C3,T1,T2,G1,G2,H1,H2,LP,LS),[0 1],[0.5 0.5 0.95]);

points=1:2:length(t);

plot(t,y(:,1),'m--','linewidth',1,'markersize',5,'markerindices',points);

hold on;

set(0,'defaultfigurecolor','w')

grid on

hold on

xlabel('$t$','interpreter','latex','Rotation',0);

ylabel('$x$','interpreter','latex');

set(gca,'XTick',[0:0.002:0.01],'YTick',[0.4:0.1:1])

axis([0 0.01 0.4 1]);

legend('z=0.01','z=0.15','z=0.55','z=0.95');

subplot(2,2,2);

a=0.85, b=0.68, R1=800, W=80,C1=180,S1=100,S2=70,H1=240,H2=300,T1=300,T2=350, C2=120, C3=100,G1=180, G2=140, LP=1.2,LS=1.6;

[t,y]=ode45(@(t,y) Datefunction(t,y,a,b,R1,W,S1,S2,C1,C2,C3,T1,T2,G1,G2,H1,H2,LP,LS),[0 1],[0.5 0.5 0.01]);

points=1:2:length(t); %ÖÐ¼äµÄ1¸ÄÎª´óÒ»µãµÄÕýÕûÊý£¬ÄÇÃ´Í¼ÏóÉÏµÄµã¾Í»á±äµÃÏ¡Êè

plot(t,y(:,2),'rh:','linewidth',1,'markersize',5,'markerindices',points);

hold on;

[t,y]=ode45(@(t,y) Datefunction(t,y,a,b,R1,W,S1,S2,C1,C2,C3,T1,T2,G1,G2,H1,H2,LP,LS),[0 1],[0.5 0.5 0.15]);

points=1:2:length(t);

plot(t,y(:,2),'b:','linewidth',1,'markersize',5,'markerindices',points);

hold on;

[t,y]=ode45(@(t,y) Datefunction(t,y,a,b,R1,W,S1,S2,C1,C2,C3,T1,T2,G1,G2,H1,H2,LP,LS),[0 1],[0.5 0.5 0.55]);

points=1:2:length(t);

plot(t,y(:,2),'g:.','linewidth',1,'markersize',5,'markerindices',points);

hold on;

[t,y]=ode45(@(t,y) Datefunction(t,y,a,b,R1,W,S1,S2,C1,C2,C3,T1,T2,G1,G2,H1,H2,LP,LS),[0 1],[0.5 0.5 0.95]);

points=1:1:length(t);

plot(t,y(:,2),'m--','linewidth',1,'markersize',5,'markerindices',points);

hold on;

set(0,'defaultfigurecolor','w')

grid on

hold on

xlabel('$t$','interpreter','latex','Rotation',0);

ylabel('$y$','interpreter','latex');

set(gca,'XTick',[0:0.01:0.1],'YTick',[0.4:0.1:1])

axis([0 0.1 0.4 1]);

legend('z=0.01','z=0.15','z=0.55','z=0.95');
