# Supplementary material for: Data-driven mechanisms for network freight platforms: An evolutionary game perspective
Source: PLoS One. 2025 Jun 27;20(6):e0319842. doi: 10.1371/journal.pone.0319842 (PMC12204633; doi:10.1371/journal.pone.0319842)
Supplement: S1 File — (ZIP) [file pone.0319842.s001.zip › Programs/Fig9-Fig21.docx]

1. **Fig 9 The influence of the benefit** $\boldsymbol{R}_{\boldsymbol{1}}$**from DDM**

clc,clear;

figure(6);

%R1=100

a=0.85, b=0.68, R1=100, W=80,C1=180,S1=100,S2=70,H1=240,H2=300,T1=300,T2=350, C2=120, C3=100,G1=180, G2=140, LP=1.2,LS=1.6;

[t,y]=ode45(@(t,y) Datefunction(t,y,a,b,R1,W,S1,S2,C1,C2,C3,T1,T2,G1,G2,H1,H2,LP,LS),[0 100],[0.2 0.2 0.2]);

plot3(y(:,1),y(:,2),y(:,3),'g:.','linewidth',1);

hold on ;

%R1=300

a=0.85, b=0.68, R1=300, W=80,C1=180,S1=100,S2=70,H1=240,H2=300,T1=300,T2=350, C2=120, C3=100,G1=180, G2=140, LP=1.2,LS=1.6;

[t,y]=ode45(@(t,y) Datefunction(t,y,a,b,R1,W,S1,S2,C1,C2,C3,T1,T2,G1,G2,H1,H2,LP,LS),[0 100],[0.2 0.2 0.2]);

plot3(y(:,1),y(:,2),y(:,3),'r+','linewidth',1);

hold on ;

%R1=500

a=0.85, b=0.68, R1=500, W=80,C1=180,S1=100,S2=70,H1=240,H2=300,T1=300,T2=350, C2=120, C3=100,G1=180, G2=140, LP=1.2,LS=1.6;

[t,y]=ode45(@(t,y) Datefunction(t,y,a,b,R1,W,S1,S2,C1,C2,C3,T1,T2,G1,G2,H1,H2,LP,LS),[0 100],[0.2 0.2 0.2]);

plot3(y(:,1),y(:,2),y(:,3),'k--','linewidth',1);

hold on ;

%R1=800

a=0.85, b=0.68, R1=800, W=80,C1=180,S1=100,S2=70,H1=240,H2=300,T1=300,T2=350, C2=120, C3=100,G1=180, G2=140, LP=1.2,LS=1.6;

[t,y]=ode45(@(t,y) Datefunction(t,y,a,b,R1,W,S1,S2,C1,C2,C3,T1,T2,G1,G2,H1,H2,LP,LS),[0 100],[0.2 0.2 0.2]);

plot3(y(:,1),y(:,2),y(:,3),'b-','linewidth',1);

hold on ;

set(gca,'XTick',[0:0.2:1],'YTick',[0:0.2:1],'ZTick',[0:0.2:1])

axis([0 1 0 1 0 1])

xlabel('$x$','interpreter','latex');ylabel('$y$','interpreter','latex');zlabel('$z$','interpreter','latex','Rotation',360,'position',[-0.1 1 1.1]);

grid on

hold on

set(0,'defaultfigurecolor','w')

legend({'{\it\fontname{Bodoni MT}R_{1}}=100','{\it\fontname{Bodoni MT}R_{1}}=300','{\it\fontname{Bodoni MT}R_{1}}=500','{\it\fontname{Bodoni MT}R_{1}}=800'},'location','northeast');

text(0.4 ,0.2 ,0.3,'$ESS$','interpreter','latex');

annotation('arrow',[0.55 0.35],[0.35 0.32]);

annotation('arrow',[0.58 0.58],[0.38 0.45]);

axes('position',[0.13 0.32 0.2 0.2]);

a=0.85, b=0.68, R1=100, W=80,C1=180,S1=100,S2=70,H1=240,H2=300,T1=300,T2=350, C2=120, C3=100,G1=180, G2=140, LP=1.2,LS=1.6;

[t,y]=ode45(@(t,y) Datefunction(t,y,a,b,R1,W,S1,S2,C1,C2,C3,T1,T2,G1,G2,H1,H2,LP,LS),[0 100],[0.2 0.2 0.2]);

plot3(y(:,1),y(:,2),y(:,3),'g:.','linewidth',1);

% stem3(y(:,1),y(:,2),y(:,3),'r+','linewidth',1);

hold on

a=0.85, b=0.68, R1=300, W=80,C1=180,S1=100,S2=70,H1=240,H2=300,T1=300,T2=350, C2=120, C3=100,G1=180, G2=140, LP=1.2,LS=1.6;

[t,y]=ode45(@(t,y) Datefunction(t,y,a,b,R1,W,S1,S2,C1,C2,C3,T1,T2,G1,G2,H1,H2,LP,LS),[0 100],[0.2 0.2 0.2]);

plot3(y(:,1),y(:,2),y(:,3),'r+','linewidth',1);

% stem3(y(:,1),y(:,2),y(:,3),'g--','linewidth',1)

a=0.85, b=0.68, R1=500, W=80,C1=180,S1=100,S2=70,H1=240,H2=300,T1=300,T2=350, C2=120, C3=100,G1=180, G2=140, LP=1.2,LS=1.6;

[t,y]=ode45(@(t,y) Datefunction(t,y,a,b,R1,W,S1,S2,C1,C2,C3,T1,T2,G1,G2,H1,H2,LP,LS),[0 100],[0.2 0.2 0.2]);

plot3(y(:,1),y(:,2),y(:,3),'k--','linewidth',1);

hold on

a=0.85, b=0.68, R1=800, W=80,C1=180,S1=100,S2=70,H1=240,H2=300,T1=300,T2=350, C2=120, C3=100,G1=180, G2=140, LP=1.2,LS=1.6;

[t,y]=ode45(@(t,y) Datefunction(t,y,a,b,R1,W,S1,S2,C1,C2,C3,T1,T2,G1,G2,H1,H2,LP,LS),[0 100],[0.2 0.2 0.2]);

plot3(y(:,1),y(:,2),y(:,3),'b-','linewidth',1);

% stem3(y(:,1),y(:,2),y(:,3),'g--','linewidth',1)

hold on

set(gca,'XTick',[0:0.2:1],'YTick',[0:0.2:1],'ZTick',[0:0.2:1])

axis([0 1 0 1 0 1])

set(gca,'XTickLabel','','YTickLabel','','ZTickLabel','')

grid on

hold on

set(0,'defaultfigurecolor','w')

view([0 0]); %z-x小图

xlabel('x','position',[0.8 1 0.3])

zlabel('z','position',[0.1 1 0.8],'Rotation',360)

1. **Fig 10. The influence of the subsidy** $\boldsymbol{S}_{\mathbf{1}}$

clc,clear;

figure(10);

%S1=80

a=0.85, b=0.68, R1=800, W=80,C1=180,S1=80,S2=70,H1=240,H2=300,T1=300,T2=350, C2=120, C3=100,G1=180, G2=140;, LP=1.2,LS=1.6

[t,y]=ode45(@(t,y) Datefunction(t,y,a,b,R1,W,S1,S2,C1,C2,C3,T1,T2,G1,G2,H1,H2,LP,LS),[0 100],[0.2 0.2 0.2]);

plot3(y(:,1),y(:,2),y(:,3),'g:.','linewidth',1);

hold on ;

%S1=100

a=0.85, b=0.68, R1=800, W=80,C1=180,S1=100,S2=70,H1=240,H2=300,T1=300,T2=350, C2=120, C3=100,G1=180, G2=140, LP=1.2,LS=1.6;

[t,y]=ode45(@(t,y) Datefunction(t,y,a,b,R1,W,S1,S2,C1,C2,C3,T1,T2,G1,G2,H1,H2,LP,LS),[0 100],[0.2 0.2 0.2]);

plot3(y(:,1),y(:,2),y(:,3),'r+','linewidth',1);

hold on ;

%S1=120

a=0.85, b=0.68, R1=800, W=80,C1=180,S1=120,S2=70,H1=240,H2=300,T1=300,T2=350, C2=120, C3=100,G1=180, G2=140, LP=1.2,LS=1.6;

[t,y]=ode45(@(t,y) Datefunction(t,y,a,b,R1,W,S1,S2,C1,C2,C3,T1,T2,G1,G2,H1,H2,LP,LS),[0 100],[0.2 0.2 0.2]);

plot3(y(:,1),y(:,2),y(:,3),'k--','linewidth',1);

hold on ;

%S1=140

a=0.85, b=0.68, R1=800, W=80,C1=180,S1=140,S2=70,H1=240,H2=300,T1=300,T2=350, C2=120, C3=100,G1=180, G2=140, LP=1.2,LS=1.6;

[t,y]=ode45(@(t,y) Datefunction(t,y,a,b,R1,W,S1,S2,C1,C2,C3,T1,T2,G1,G2,H1,H2,LP,LS),[0 100],[0.2 0.2 0.2]);

plot3(y(:,1),y(:,2),y(:,3),'b-','linewidth',1);

hold on ;

set(gca,'XTick',[0:0.2:1],'YTick',[0:0.2:1],'ZTick',[0:0.2:1])

axis([0 1 0 1 0 1])

xlabel('$x$','interpreter','latex');ylabel('$y$','interpreter','latex');zlabel('$z$','interpreter','latex','Rotation',360,'position',[-0.1 1 1.1]);

grid on

hold on

set(0,'defaultfigurecolor','w')

legend({'{\it\fontname{Bodoni MT}S_{1}}=80','{\it\fontname{Bodoni MT}S_{1}}=100','{\it\fontname{Bodoni MT}S_{1}}=120','{\it\fontname{Bodoni MT}S_{1}}=140'},'location','northeast');

text(0.4 ,0.2 ,0.3,'$ESS$','interpreter','latex');

annotation('arrow',[0.55 0.35],[0.35 0.32]);

annotation('arrow',[0.58 0.58],[0.38 0.45]);

a=0.85, b=0.68, R1=800, W=80,C1=180,S1=80,S2=70,H1=240,H2=300,T1=300,T2=350, C2=120, C3=100,G1=180, G2=140, LP=1.2,LS=1.6;

[t,y]=ode45(@(t,y) Datefunction(t,y,a,b,R1,W,S1,S2,C1,C2,C3,T1,T2,G1,G2,H1,H2,LP,LS),[0 100],[0.2 0.2 0.2]);

plot3(y(:,1),y(:,2),y(:,3),'g:.','linewidth',1);

hold on

a=0.85, b=0.68, R1=800, W=80,C1=180,S1=100,S2=70,H1=240,H2=300,T1=300,T2=350, C2=120, C3=100,G1=180, G2=140, LP=1.2,LS=1.6;

[t,y]=ode45(@(t,y) Datefunction(t,y,a,b,R1,W,S1,S2,C1,C2,C3,T1,T2,G1,G2,H1,H2,LP,LS),[0 100],[0.2 0.2 0.2]);

plot3(y(:,1),y(:,2),y(:,3),'r+','linewidth',1);

a=0.85, b=0.68, R1=800, W=80,C1=180,S1=120,S2=70,H1=240,H2=300,T1=300,T2=350, C2=120, C3=100,G1=180, G2=140, LP=1.2,LS=1.6;

[t,y]=ode45(@(t,y) Datefunction(t,y,a,b,R1,W,S1,S2,C1,C2,C3,T1,T2,G1,G2,H1,H2,LP,LS),[0 100],[0.2 0.2 0.2]);

plot3(y(:,1),y(:,2),y(:,3),'k--','linewidth',1);

hold on

a=0.85, b=0.68, R1=800, W=80,C1=180,S1=140,S2=70,H1=240,H2=300,T1=300,T2=350, C2=120, C3=100,G1=180, G2=140, LP=1.2,LS=1.6;

[t,y]=ode45(@(t,y) Datefunction(t,y,a,b,R1,W,S1,S2,C1,C2,C3,T1,T2,G1,G2,H1,H2,LP,LS),[0 100],[0.2 0.2 0.2]);

plot3(y(:,1),y(:,2),y(:,3),'b-','linewidth',1);

hold on

set(gca,'XTick',[0:0.2:1],'YTick',[0:0.2:1],'ZTick',[0:0.2:1])

axis([0 1 0 1 0 1])

set(gca,'XTickLabel','','YTickLabel','','ZTickLabel','')

grid on

hold on

set(0,'defaultfigurecolor','w')

view([0 0]); %z-xÐ¡Í¼

xlabel('x','position',[0.8 1 0.3])

zlabel('z','position',[0.1 1 0.8],'Rotation',360)

1. **Fig 11. The influence of the subsidy** $\boldsymbol{S}_{\mathbf{2}}$

clc,clear;

figure(11);

%S2=80

a=0.85, b=0.68, R1=800, W=80,C1=180,S1=100,S2=80,H1=240,H2=300,T1=300,T2=350, C2=120, C3=100,G1=180, G2=140, LP=1.2,LS=1.6;

[t,y]=ode45(@(t,y) Datefunction(t,y,a,b,R1,W,S1,S2,C1,C2,C3,T1,T2,G1,G2,H1,H2,LP,LS),[0 100],[0.2 0.2 0.2]);

plot3(y(:,1),y(:,2),y(:,3),'g:.','linewidth',1);

hold on ;

%S2=100

a=0.85, b=0.68, R1=800, W=80,C1=180,S1=100,S2=100,H1=240,H2=300,T1=300,T2=350, C2=120, C3=100,G1=180, G2=140, LP=1.2,LS=1.6;

[t,y]=ode45(@(t,y) Datefunction(t,y,a,b,R1,W,S1,S2,C1,C2,C3,T1,T2,G1,G2,H1,H2,LP,LS),[0 100],[0.2 0.2 0.2]);

plot3(y(:,1),y(:,2),y(:,3),'r+','linewidth',1);

hold on ;

%S2=120

a=0.85, b=0.68, R1=800, W=80,C1=180,S1=100,S2=120,H1=240,H2=300,T1=300,T2=350, C2=120, C3=100,G1=180, G2=140, LP=1.2,LS=1.6;

[t,y]=ode45(@(t,y) Datefunction(t,y,a,b,R1,W,S1,S2,C1,C2,C3,T1,T2,G1,G2,H1,H2,LP,LS),[0 100],[0.2 0.2 0.2]);

plot3(y(:,1),y(:,2),y(:,3),'k--','linewidth',1);

hold on ;

%S2=140

a=0.85, b=0.68, R1=800, W=80,C1=180,S1=100,S2=140,H1=240,H2=300,T1=300,T2=350, C2=120, C3=100,G1=180, G2=140, LP=1.2,LS=1.6;

[t,y]=ode45(@(t,y) Datefunction(t,y,a,b,R1,W,S1,S2,C1,C2,C3,T1,T2,G1,G2,H1,H2,LP,LS),[0 100],[0.2 0.2 0.2]);

plot3(y(:,1),y(:,2),y(:,3),'b-','linewidth',1);

hold on ;

set(gca,'XTick',[0:0.2:1],'YTick',[0:0.2:1],'ZTick',[0:0.2:1])

axis([0 1 0 1 0 1])

xlabel('$x$','interpreter','latex');ylabel('$y$','interpreter','latex');zlabel('$z$','interpreter','latex','Rotation',360,'position',[-0.1 1 1.1]);

grid on

hold on

set(0,'defaultfigurecolor','w')

legend({'{\it\fontname{Bodoni MT}S_{2}}=80','{\it\fontname{Bodoni MT}S_{2}}=100','{\it\fontname{Bodoni MT}S_{2}}=120','{\it\fontname{Bodoni MT}S_{2}}=140'},'location','northeast');

text(0.4 ,0.2 ,0.3,'$ESS$','interpreter','latex');

annotation('arrow',[0.55 0.35],[0.35 0.32]);

annotation('arrow',[0.58 0.58],[0.38 0.45]);

axes('position',[0.13 0.32 0.2 0.2]); %Ð¡Í¼µÄ¹Ø¼üÓï¾äÈ·¶¨Ð¡Í¼µÄ´óÐ¡Î»ÖÃ

a=0.85, b=0.68, R1=800, W=80,C1=180,S1=100,S2=80,H1=240,H2=300,T1=300,T2=350, C2=120, C3=100,G1=180, G2=140, LP=1.2,LS=1.6;

[t,y]=ode45(@(t,y) Datefunction(t,y,a,b,R1,W,S1,S2,C1,C2,C3,T1,T2,G1,G2,H1,H2,LP,LS),[0 100],[0.2 0.2 0.2]);

plot3(y(:,1),y(:,2),y(:,3),'g:.','linewidth',1);

hold on

a=0.85, b=0.68, R1=800, W=80,C1=180,S1=100,S2=100,H1=240,H2=300,T1=300,T2=350, C2=120, C3=100,G1=180, G2=140, LP=1.2,LS=1.6;

[t,y]=ode45(@(t,y) Datefunction(t,y,a,b,R1,W,S1,S2,C1,C2,C3,T1,T2,G1,G2,H1,H2,LP,LS),[0 100],[0.2 0.2 0.2]);

plot3(y(:,1),y(:,2),y(:,3),'r+','linewidth',1);

a=0.85, b=0.68, R1=800, W=80,C1=180,S1=100,S2=120,H1=240,H2=300,T1=300,T2=350, C2=120, C3=100,G1=180, G2=140, LP=1.2,LS=1.6;

[t,y]=ode45(@(t,y) Datefunction(t,y,a,b,R1,W,S1,S2,C1,C2,C3,T1,T2,G1,G2,H1,H2,LP,LS),[0 100],[0.2 0.2 0.2]);

plot3(y(:,1),y(:,2),y(:,3),'k--','linewidth',1);

hold on

a=0.85, b=0.68, R1=800, W=80,C1=180,S1=100,S2=140,H1=240,H2=300,T1=300,T2=350, C2=120, C3=100,G1=180, G2=140, LP=1.2,LS=1.6;

[t,y]=ode45(@(t,y) Datefunction(t,y,a,b,R1,W,S1,S2,C1,C2,C3,T1,T2,G1,G2,H1,H2,LP,LS),[0 100],[0.2 0.2 0.2]);

plot3(y(:,1),y(:,2),y(:,3),'b-','linewidth',1);

hold on

set(gca,'XTick',[0:0.2:1],'YTick',[0:0.2:1],'ZTick',[0:0.2:1])

axis([0 1 0 1 0 1])

set(gca,'XTickLabel','','YTickLabel','','ZTickLabel','')

grid on

hold on

set(0,'defaultfigurecolor','w')

view([0 0]); %z-xÐ¡Í¼

xlabel('x','position',[0.8 1 0.3])

zlabel('z','position',[0.1 1 0.8],'Rotation',360)

1. **Fig 12. The influence of penalty** $\boldsymbol{T}_{\mathbf{1}}$

clc,clear;

figure(12);

%T1=200

a=0.85, b=0.68, R1=800, W=80,C1=180,S1=100,S2=70,H1=240,H2=300,T1=200,T2=350, C2=120, C3=100,G1=180, G2=140;

[t,y]=ode45(@(t,y) Datefunction(t,y,a,b,R1,W,S1,S2,C1,C2,C3,T1,T2,G1,G2,H1,H2),[0 100],[0.2 0.2 0.2]);

plot3(y(:,1),y(:,2),y(:,3),'g:.','linewidth',1);

hold on ;

%T1=250

a=0.85, b=0.68, R1=800, W=80,C1=180,S1=100,S2=70,H1=240,H2=300,T1=250,T2=350, C2=120, C3=100,G1=180, G2=140;

[t,y]=ode45(@(t,y) Datefunction(t,y,a,b,R1,W,S1,S2,C1,C2,C3,T1,T2,G1,G2,H1,H2),[0 100],[0.2 0.2 0.2]);

plot3(y(:,1),y(:,2),y(:,3),'r+','linewidth',1);

hold on ;

%T1=300

a=0.85, b=0.68, R1=800, W=80,C1=180,S1=100,S2=70,H1=240,H2=300,T1=300,T2=350, C2=120, C3=100,G1=180, G2=140;

[t,y]=ode45(@(t,y) Datefunction(t,y,a,b,R1,W,S1,S2,C1,C2,C3,T1,T2,G1,G2,H1,H2),[0 100],[0.2 0.2 0.2]);

plot3(y(:,1),y(:,2),y(:,3),'k--','linewidth',1);

hold on ;

%T1=350

a=0.85, b=0.68, R1=800, W=80,C1=180,S1=100,S2=70,H1=240,H2=300,T1=350,T2=350, C2=120, C3=100,G1=180, G2=140;

[t,y]=ode45(@(t,y) Datefunction(t,y,a,b,R1,W,S1,S2,C1,C2,C3,T1,T2,G1,G2,H1,H2),[0 100],[0.2 0.2 0.2]);

plot3(y(:,1),y(:,2),y(:,3),'b-','linewidth',1);

hold on ;

set(gca,'XTick',[0:0.2:1],'YTick',[0:0.2:1],'ZTick',[0:0.2:1])

axis([0 1 0 1 0 1])

xlabel('$x$','interpreter','latex');ylabel('$y$','interpreter','latex');zlabel('$z$','interpreter','latex','Rotation',360,'position',[-0.1 1 1.1]);

grid on

hold on

set(0,'defaultfigurecolor','w')

legend({'{\it\fontname{Bodoni MT}T_{1}}=200','{\it\fontname{Bodoni MT}T_{1}}=250','{\it\fontname{Bodoni MT}T_{1}}=300','{\it\fontname{Bodoni MT}T_{1}}=350'},'location','northeast');

text(0.4 ,0.2 ,0.3,'$ESS$','interpreter','latex');

annotation('arrow',[0.55 0.35],[0.35 0.32]);

annotation('arrow',[0.58 0.58],[0.38 0.45]);

axes('position',[0.13 0.32 0.2 0.2]); %Ð¡Í¼µÄ¹Ø¼üÓï¾äÈ·¶¨Ð¡Í¼µÄ´óÐ¡Î»ÖÃ

a=0.85, b=0.68, R1=800, W=80,C1=180,S1=100,S2=70,H1=240,H2=300,T1=200,T2=350, C2=120, C3=100,G1=180, G2=140;

[t,y]=ode45(@(t,y) Datefunction(t,y,a,b,R1,W,S1,S2,C1,C2,C3,T1,T2,G1,G2,H1,H2),[0 100],[0.2 0.2 0.2]);

plot3(y(:,1),y(:,2),y(:,3),'g:.','linewidth',1);

hold on

a=0.85, b=0.68, R1=800, W=80,C1=180,S1=100,S2=70,H1=240,H2=300,T1=250,T2=350, C2=120, C3=100,G1=180, G2=140;

[t,y]=ode45(@(t,y) Datefunction(t,y,a,b,R1,W,S1,S2,C1,C2,C3,T1,T2,G1,G2,H1,H2),[0 100],[0.2 0.2 0.2]);

plot3(y(:,1),y(:,2),y(:,3),'r+','linewidth',1);

a=0.85, b=0.68, R1=800, W=80,C1=180,S1=100,S2=70,H1=240,H2=300,T1=300,T2=350, C2=120, C3=100,G1=180, G2=140;

[t,y]=ode45(@(t,y) Datefunction(t,y,a,b,R1,W,S1,S2,C1,C2,C3,T1,T2,G1,G2,H1,H2),[0 100],[0.2 0.2 0.2]);

plot3(y(:,1),y(:,2),y(:,3),'k--','linewidth',1);

hold on

a=0.85, b=0.68, R1=800, W=80,C1=180,S1=100,S2=70,H1=240,H2=300,T1=350,T2=350, C2=120, C3=100,G1=180, G2=140;

[t,y]=ode45(@(t,y) Datefunction(t,y,a,b,R1,W,S1,S2,C1,C2,C3,T1,T2,G1,G2,H1,H2),[0 100],[0.2 0.2 0.2]);

plot3(y(:,1),y(:,2),y(:,3),'b-','linewidth',1);

hold on

set(gca,'XTick',[0:0.2:1],'YTick',[0:0.2:1],'ZTick',[0:0.2:1])

axis([0 1 0 1 0 1])

set(gca,'XTickLabel','','YTickLabel','','ZTickLabel','')

grid on

hold on

set(0,'defaultfigurecolor','w')

view([0 0]); %z-xÐ¡Í¼

xlabel('x','position',[0.8 1 0.3])

zlabel('z','position',[0.1 1 0.8],'Rotation',360)

1. **Fig 13. The influence of penalty** $\boldsymbol{T}_{\mathbf{2}}$

clc,clear;

figure(13);

%T2=200

a=0.85, b=0.68, R1=800, W=80,C1=180,S1=100,S2=70,H1=240,H2=300,T1=300,T2=200, C2=120, C3=100,G1=180, G2=140;

[t,y]=ode45(@(t,y) Datefunction(t,y,a,b,R1,W,S1,S2,C1,C2,C3,T1,T2,G1,G2,H1,H2),[0 100],[0.2 0.2 0.2]);

plot3(y(:,1),y(:,2),y(:,3),'g:.','linewidth',1);

hold on ;

%T2=250

a=0.85, b=0.68, R1=800, W=80,C1=180,S1=100,S2=70,H1=240,H2=300,T1=300,T2=250, C2=120, C3=100,G1=180, G2=140;

[t,y]=ode45(@(t,y) Datefunction(t,y,a,b,R1,W,S1,S2,C1,C2,C3,T1,T2,G1,G2,H1,H2),[0 100],[0.2 0.2 0.2]);

plot3(y(:,1),y(:,2),y(:,3),'r+','linewidth',1);

%stem3(y(:,1),y(:,2),y(:,3),'r+','linewidth',1); %ÈýÎ¬»ð²ñ¹÷Í¼

hold on ;

%T2=300

a=0.85, b=0.68, R1=800, W=80,C1=180,S1=100,S2=70,H1=240,H2=300,T1=300,T2=350, C2=120, C3=100,G1=180, G2=140;

[t,y]=ode45(@(t,y) Datefunction(t,y,a,b,R1,W,S1,S2,C1,C2,C3,T1,T2,G1,G2,H1,H2),[0 100],[0.2 0.2 0.2]);

plot3(y(:,1),y(:,2),y(:,3),'k--','linewidth',1);

hold on ;

%T2=350

a=0.85, b=0.68, R1=800, W=80,C1=180,S1=100,S2=70,H1=240,H2=300,T1=300,T2=350, C2=120, C3=100,G1=180, G2=140;

[t,y]=ode45(@(t,y) Datefunction(t,y,a,b,R1,W,S1,S2,C1,C2,C3,T1,T2,G1,G2,H1,H2),[0 100],[0.2 0.2 0.2]);

plot3(y(:,1),y(:,2),y(:,3),'b-','linewidth',1);

hold on ;

%×ø±ê¿Ì¶È¼ä¸ô¼°ÆäÇø¼ä£¬×ø±ê±ê×¢

set(gca,'XTick',[0:0.2:1],'YTick',[0:0.2:1],'ZTick',[0:0.2:1])

axis([0 1 0 1 0 1])

xlabel('$x$','interpreter','latex');ylabel('$y$','interpreter','latex');zlabel('$z$','interpreter','latex','Rotation',360,'position',[-0.1 1 1.1]);

%Í¼ÏóÍø¸ñ£¬µ×Í¼¼Ó°×

grid on

hold on

set(0,'defaultfigurecolor','w')

%Í¼Àý¼°±êÌâ

legend({'{\it\fontname{Bodoni MT}T_{2}}=200','{\it\fontname{Bodoni MT}T_{2}}=250','{\it\fontname{Bodoni MT}T_{2}}=300','{\it\fontname{Bodoni MT}T_{2}}=350'},'location','northeast');

%title('Fig. 8. The influence of the incentives cost C_{1}. ','FontWeight','bold','position',[0 0 -0.2]);

%¼ÓÉÏ±ê×¢ºÍ¼ýÍ·

text(0.4 ,0.2 ,0.3,'$ESS$','interpreter','latex');

annotation('arrow',[0.55 0.35],[0.35 0.32]);

annotation('arrow',[0.58 0.58],[0.38 0.45]);

% the small figure

axes('position',[0.13 0.32 0.2 0.2]); %Ð¡Í¼µÄ¹Ø¼üÓï¾äÈ·¶¨Ð¡Í¼µÄ´óÐ¡Î»ÖÃ

% z-xÐ¡Í¼ÖÐµÄÏßÌõ

%Ð¡Í¼ÖÐµÄÏßÌõ

a=0.85, b=0.68, R1=800, W=80,C1=180,S1=100,S2=70,H1=240,H2=300,T1=200,T2=350, C2=120, C3=100,G1=180, G2=140;

[t,y]=ode45(@(t,y) Datefunction(t,y,a,b,R1,W,S1,S2,C1,C2,C3,T1,T2,G1,G2,H1,H2),[0 100],[0.2 0.2 0.2]);

plot3(y(:,1),y(:,2),y(:,3),'g:.','linewidth',1);

% stem3(y(:,1),y(:,2),y(:,3),'r+','linewidth',1);%»ð²ñ¹÷Í¼

hold on

a=0.85, b=0.68, R1=800, W=80,C1=180,S1=100,S2=70,H1=240,H2=300,T1=250,T2=350, C2=120, C3=100,G1=180, G2=140;

[t,y]=ode45(@(t,y) Datefunction(t,y,a,b,R1,W,S1,S2,C1,C2,C3,T1,T2,G1,G2,H1,H2),[0 100],[0.2 0.2 0.2]);

plot3(y(:,1),y(:,2),y(:,3),'r+','linewidth',1);

% stem3(y(:,1),y(:,2),y(:,3),'g--','linewidth',1);%»ð²ñ¹÷Í¼

a=0.85, b=0.68, R1=800, W=80,C1=180,S1=100,S2=70,H1=240,H2=300,T1=300,T2=350, C2=120, C3=100,G1=180, G2=140;

[t,y]=ode45(@(t,y) Datefunction(t,y,a,b,R1,W,S1,S2,C1,C2,C3,T1,T2,G1,G2,H1,H2),[0 100],[0.2 0.2 0.2]);

plot3(y(:,1),y(:,2),y(:,3),'k--','linewidth',1);

hold on

a=0.85, b=0.68, R1=800, W=80,C1=180,S1=100,S2=70,H1=240,H2=300,T1=350,T2=350, C2=120, C3=100,G1=180, G2=140;

[t,y]=ode45(@(t,y) Datefunction(t,y,a,b,R1,W,S1,S2,C1,C2,C3,T1,T2,G1,G2,H1,H2),[0 100],[0.2 0.2 0.2]);

plot3(y(:,1),y(:,2),y(:,3),'b-','linewidth',1);

% stem3(y(:,1),y(:,2),y(:,3),'g--','linewidth',1);%»ð²ñ¹÷Í¼

hold on

%×ø±ê¿Ì¶È¼ä¸ô¼°Çø¼ä£¬µ«²»ÏÔÊ¾

set(gca,'XTick',[0:0.2:1],'YTick',[0:0.2:1],'ZTick',[0:0.2:1])

axis([0 1 0 1 0 1])

set(gca,'XTickLabel','','YTickLabel','','ZTickLabel','')

%±³¾°Íø¸ñ²¢ÉèÖÃ°×µ×

grid on

hold on

set(0,'defaultfigurecolor','w')

%Í¼ÏóÏÔÊ¾ÊÓ½Ç£¬ÒÔ¼°ÏàÓ¦µÄ×ø±ê±ê×¢

view([0 0]); %z-xÐ¡Í¼

xlabel('x','position',[0.8 1 0.3])

zlabel('z','position',[0.1 1 0.8],'Rotation',360)

**6 Fig 14. The influence of the probability** $\boldsymbol{\alpha}$ **of the carrier’s or the shipper’s dishonest strategy discovered by the NFP’s choosing DDM** a=0.7, b=0.68, R1=800, W=80,C1=180,S1=100,S2=70,H1=240,H2=300,T1=300,T2=350, C2=120, C3=100,G1=180, G2=140;

[t,y]=ode45(@(t,y) Datefunction(t,y,a,b,R1,W,S1,S2,C1,C2,C3,T1,T2,G1,G2,H1,H2),[0 100],[0.2 0.2 0.2]);

plot3(y(:,1),y(:,2),y(:,3),'g:.','linewidth',1);

hold on ;

%a=0.75

a=0.75, b=0.68, R1=800, W=80,C1=180,S1=100,S2=70,H1=240,H2=300,T1=300,T2=350, C2=120, C3=100,G1=180, G2=140;

[t,y]=ode45(@(t,y) Datefunction(t,y,a,b,R1,W,S1,S2,C1,C2,C3,T1,T2,G1,G2,H1,H2),[0 100],[0.2 0.2 0.2]);

plot3(y(:,1),y(:,2),y(:,3),'r+','linewidth',1);

%stem3(y(:,1),y(:,2),y(:,3),'r+','linewidth',1); %ÈýÎ¬»ð²ñ¹÷Í¼

hold on ;

%a=0.80

a=0.80, b=0.68, R1=800, W=80,C1=180,S1=100,S2=70,H1=240,H2=300,T1=300,T2=350, C2=120, C3=100,G1=180, G2=140;

[t,y]=ode45(@(t,y) Datefunction(t,y,a,b,R1,W,S1,S2,C1,C2,C3,T1,T2,G1,G2,H1,H2),[0 100],[0.2 0.2 0.2]);

plot3(y(:,1),y(:,2),y(:,3),'k--','linewidth',1);

hold on ;

%a=0.85

a=0.85, b=0.68, R1=800, W=80,C1=180,S1=100,S2=70,H1=240,H2=300,T1=300,T2=350, C2=120, C3=100,G1=180, G2=140;

[t,y]=ode45(@(t,y) Datefunction(t,y,a,b,R1,W,S1,S2,C1,C2,C3,T1,T2,G1,G2,H1,H2),[0 100],[0.2 0.2 0.2]);

plot3(y(:,1),y(:,2),y(:,3),'b-','linewidth',1);

hold on ;

%×ø±ê¿Ì¶È¼ä¸ô¼°ÆäÇø¼ä£¬×ø±ê±ê×¢

set(gca,'XTick',[0:0.2:1],'YTick',[0:0.2:1],'ZTick',[0:0.2:1])

axis([0 1 0 1 0 1])

xlabel('$x$','interpreter','latex');ylabel('$y$','interpreter','latex');zlabel('$z$','interpreter','latex','Rotation',360,'position',[-0.1 1 1.1]);

%Í¼ÏóÍø¸ñ£¬µ×Í¼¼Ó°×

grid on

hold on

set(0,'defaultfigurecolor','w')

%Í¼Àý¼°±êÌâ

legend({'{\it\fontname{Bodoni MT} \alpha}=0.70','{\it\fontname{Bodoni MT} \alpha}=0.75','{\it\fontname{Bodoni MT} \alpha}=0.80','{\it\fontname{Bodoni MT} \alpha}=0.85'},'location','northeast');

%title('Fig. 8. The influence of the incentives cost C_{1}. ','FontWeight','bold','position',[0 0 -0.2]);

%¼ÓÉÏ±ê×¢ºÍ¼ýÍ·

text(0.4 ,0.2 ,0.3,'$ESS$','interpreter','latex');

annotation('arrow',[0.55 0.35],[0.35 0.32]);

annotation('arrow',[0.58 0.58],[0.38 0.45]);

% the small figure

axes('position',[0.13 0.32 0.2 0.2]); %Ð¡Í¼µÄ¹Ø¼üÓï¾äÈ·¶¨Ð¡Í¼µÄ´óÐ¡Î»ÖÃ

% z-xÐ¡Í¼ÖÐµÄÏßÌõ

%Ð¡Í¼ÖÐµÄÏßÌõ

a=0.70, b=0.68, R1=800, W=80,C1=180,S1=100,S2=70,H1=240,H2=300,T1=300,T2=350, C2=120, C3=100,G1=180, G2=140;

[t,y]=ode45(@(t,y) Datefunction(t,y,a,b,R1,W,S1,S2,C1,C2,C3,T1,T2,G1,G2,H1,H2),[0 100],[0.2 0.2 0.2]);

plot3(y(:,1),y(:,2),y(:,3),'g:.','linewidth',1);

% stem3(y(:,1),y(:,2),y(:,3),'r+','linewidth',1);%»ð²ñ¹÷Í¼

hold on

a=0.75, b=0.68, R1=800, W=80,C1=180,S1=100,S2=70,H1=240,H2=300,T1=300,T2=350, C2=120, C3=100,G1=180, G2=140;

[t,y]=ode45(@(t,y) Datefunction(t,y,a,b,R1,W,S1,S2,C1,C2,C3,T1,T2,G1,G2,H1,H2),[0 100],[0.2 0.2 0.2]);

plot3(y(:,1),y(:,2),y(:,3),'r+','linewidth',1);

% stem3(y(:,1),y(:,2),y(:,3),'g--','linewidth',1);%»ð²ñ¹÷Í¼

a=0.80, b=0.68, R1=800, W=80,C1=180,S1=100,S2=70,H1=240,H2=300,T1=300,T2=350, C2=120, C3=100,G1=180, G2=140;

[t,y]=ode45(@(t,y) Datefunction(t,y,a,b,R1,W,S1,S2,C1,C2,C3,T1,T2,G1,G2,H1,H2),[0 100],[0.2 0.2 0.2]);

plot3(y(:,1),y(:,2),y(:,3),'k--','linewidth',1);

hold on

a=0.85, b=0.68, R1=800, W=80,C1=180,S1=100,S2=70,H1=240,H2=300,T1=300,T2=350, C2=120, C3=100,G1=180, G2=140;

[t,y]=ode45(@(t,y) Datefunction(t,y,a,b,R1,W,S1,S2,C1,C2,C3,T1,T2,G1,G2,H1,H2),[0 100],[0.2 0.2 0.2]);

plot3(y(:,1),y(:,2),y(:,3),'b-','linewidth',1);

% stem3(y(:,1),y(:,2),y(:,3),'g--','linewidth',1);%»ð²ñ¹÷Í¼

hold on

%×ø±ê¿Ì¶È¼ä¸ô¼°Çø¼ä£¬µ«²»ÏÔÊ¾

set(gca,'XTick',[0:0.2:1],'YTick',[0:0.2:1],'ZTick',[0:0.2:1])

axis([0 1 0 1 0 1])

set(gca,'XTickLabel','','YTickLabel','','ZTickLabel','')

%±³¾°Íø¸ñ²¢ÉèÖÃ°×µ×

grid on

hold on

set(0,'defaultfigurecolor','w')

%Í¼ÏóÏÔÊ¾ÊÓ½Ç£¬ÒÔ¼°ÏàÓ¦µÄ×ø±ê±ê×¢

view([0 0]); %z-xÐ¡Í¼

xlabel('x','position',[0.8 1 0.3])

zlabel('z','position',[0.1 1 0.8],'Rotation',360)

1. **Fig 15. The influence of extra cost** $\boldsymbol{C}_{\mathbf{1}}$

clc,clear;

figure(15);

%C1=80

a=0.85, b=0.68, R1=800, W=80,C1=80,S1=100,S2=70,H1=240,H2=300,T1=300,T2=350, C2=120, C3=100,G1=180, G2=140;

[t,y]=ode45(@(t,y) Datefunction(t,y,a,b,R1,W,S1,S2,C1,C2,C3,T1,T2,G1,G2,H1,H2),[0 100],[0.2 0.2 0.2]);

plot3(y(:,1),y(:,2),y(:,3),'g:.','linewidth',1);

hold on ;

%C1=100

a=0.85, b=0.68, R1=800, W=80,C1=100,S1=100,S2=70,H1=240,H2=300,T1=300,T2=350, C2=120, C3=100,G1=180, G2=140;

[t,y]=ode45(@(t,y) Datefunction(t,y,a,b,R1,W,S1,S2,C1,C2,C3,T1,T2,G1,G2,H1,H2),[0 100],[0.2 0.2 0.2]);

plot3(y(:,1),y(:,2),y(:,3),'r+','linewidth',1);

%stem3(y(:,1),y(:,2),y(:,3),'r+','linewidth',1); %ÈýÎ¬»ð²ñ¹÷Í¼

hold on ;

%C1=120

a=0.85, b=0.68, R1=800, W=80,C1=120,S1=100,S2=70,H1=240,H2=300,T1=300,T2=350, C2=120, C3=100,G1=180, G2=140;

[t,y]=ode45(@(t,y) Datefunction(t,y,a,b,R1,W,S1,S2,C1,C2,C3,T1,T2,G1,G2,H1,H2),[0 100],[0.2 0.2 0.2]);

plot3(y(:,1),y(:,2),y(:,3),'k--','linewidth',1);

hold on ;

%C1=140

a=0.85, b=0.68, R1=800, W=80,C1=140,S1=100,S2=70,H1=240,H2=300,T1=300,T2=350, C2=120, C3=100,G1=180, G2=140;

[t,y]=ode45(@(t,y) Datefunction(t,y,a,b,R1,W,S1,S2,C1,C2,C3,T1,T2,G1,G2,H1,H2),[0 100],[0.2 0.2 0.2]);

plot3(y(:,1),y(:,2),y(:,3),'b-','linewidth',1);

hold on ;

%×ø±ê¿Ì¶È¼ä¸ô¼°ÆäÇø¼ä£¬×ø±ê±ê×¢

set(gca,'XTick',[0:0.2:1],'YTick',[0:0.2:1],'ZTick',[0:0.2:1])

axis([0 1 0 1 0 1])

xlabel('$x$','interpreter','latex');ylabel('$y$','interpreter','latex');zlabel('$z$','interpreter','latex','Rotation',360,'position',[-0.1 1 1.1]);

%Í¼ÏóÍø¸ñ£¬µ×Í¼¼Ó°×

grid on

hold on

set(0,'defaultfigurecolor','w')

%Í¼Àý¼°±êÌâ

legend({'{\it\fontname{Bodoni MT} C_{1}}=80','{\it\fontname{Bodoni MT} C_{1}}=100','{\it\fontname{Bodoni MT} C_{1}}=120','{\it\fontname{Bodoni MT} C_{1}}=140'},'location','northeast');

%title('Fig. 8. The influence of the incentives cost C_{1}. ','FontWeight','bold','position',[0 0 -0.2]);

%¼ÓÉÏ±ê×¢ºÍ¼ýÍ·

text(0.4 ,0.2 ,0.3,'$ESS$','interpreter','latex');

annotation('arrow',[0.55 0.35],[0.35 0.32]);

annotation('arrow',[0.58 0.58],[0.38 0.45]);

% the small figure

axes('position',[0.13 0.32 0.2 0.2]); %Ð¡Í¼µÄ¹Ø¼üÓï¾äÈ·¶¨Ð¡Í¼µÄ´óÐ¡Î»ÖÃ

% z-xÐ¡Í¼ÖÐµÄÏßÌõ

%Ð¡Í¼ÖÐµÄÏßÌõ

a=0.85, b=0.68, R1=800, W=80,C1=80,S1=100,S2=70,H1=240,H2=300,T1=300,T2=350, C2=120, C3=100,G1=180, G2=140;

[t,y]=ode45(@(t,y) Datefunction(t,y,a,b,R1,W,S1,S2,C1,C2,C3,T1,T2,G1,G2,H1,H2),[0 100],[0.2 0.2 0.2]);

plot3(y(:,1),y(:,2),y(:,3),'g:.','linewidth',1);

% stem3(y(:,1),y(:,2),y(:,3),'r+','linewidth',1);%»ð²ñ¹÷Í¼

hold on

a=0.85, b=0.68, R1=800, W=80,C1=100,S1=100,S2=70,H1=240,H2=300,T1=300,T2=350, C2=120, C3=100,G1=180, G2=140;

[t,y]=ode45(@(t,y) Datefunction(t,y,a,b,R1,W,S1,S2,C1,C2,C3,T1,T2,G1,G2,H1,H2),[0 100],[0.2 0.2 0.2]);

plot3(y(:,1),y(:,2),y(:,3),'r+','linewidth',1);

% stem3(y(:,1),y(:,2),y(:,3),'g--','linewidth',1);%»ð²ñ¹÷Í¼

a=0.85, b=0.68, R1=800, W=80,C1=120,S1=100,S2=70,H1=240,H2=300,T1=300,T2=350, C2=120, C3=100,G1=180, G2=140;

[t,y]=ode45(@(t,y) Datefunction(t,y,a,b,R1,W,S1,S2,C1,C2,C3,T1,T2,G1,G2,H1,H2),[0 100],[0.2 0.2 0.2]);

plot3(y(:,1),y(:,2),y(:,3),'k--','linewidth',1);

hold on

a=0.85, b=0.68, R1=800, W=80,C1=140,S1=100,S2=70,H1=240,H2=300,T1=300,T2=350, C2=120, C3=100,G1=180, G2=140;

[t,y]=ode45(@(t,y) Datefunction(t,y,a,b,R1,W,S1,S2,C1,C2,C3,T1,T2,G1,G2,H1,H2),[0 100],[0.2 0.2 0.2]);

plot3(y(:,1),y(:,2),y(:,3),'b-','linewidth',1);

% stem3(y(:,1),y(:,2),y(:,3),'g--','linewidth',1);%»ð²ñ¹÷Í¼

hold on

%×ø±ê¿Ì¶È¼ä¸ô¼°Çø¼ä£¬µ«²»ÏÔÊ¾

set(gca,'XTick',[0:0.2:1],'YTick',[0:0.2:1],'ZTick',[0:0.2:1])

axis([0 1 0 1 0 1])

set(gca,'XTickLabel','','YTickLabel','','ZTickLabel','')

%±³¾°Íø¸ñ²¢ÉèÖÃ°×µ×

grid on

hold on

set(0,'defaultfigurecolor','w')

%Í¼ÏóÏÔÊ¾ÊÓ½Ç£¬ÒÔ¼°ÏàÓ¦µÄ×ø±ê±ê×¢

view([0 0]); %z-xÐ¡Í¼

xlabel('x','position',[0.8 1 0.3])

zlabel('z','position',[0.1 1 0.8],'Rotation',360)

1. **Fig 16. Effect of extra cost** $\boldsymbol{C}_{\mathbf{2}}$

clc,clear;

figure(16);

%C2=80

a=0.85, b=0.68, R1=800, W=80,C1=180,S1=100,S2=70,H1=240,H2=300,T1=300,T2=350, C2=80, C3=100,G1=180, G2=140;

[t,y]=ode45(@(t,y) Datefunction(t,y,a,b,R1,W,S1,S2,C1,C2,C3,T1,T2,G1,G2,H1,H2),[0 100],[0.2 0.2 0.2]);

plot3(y(:,1),y(:,2),y(:,3),'g:.','linewidth',1);

hold on ;

%C2=100

a=0.85, b=0.68, R1=800, W=80,C1=180,S1=100,S2=70,H1=240,H2=300,T1=300,T2=350, C2=100, C3=100,G1=180, G2=140;

[t,y]=ode45(@(t,y) Datefunction(t,y,a,b,R1,W,S1,S2,C1,C2,C3,T1,T2,G1,G2,H1,H2),[0 100],[0.2 0.2 0.2]);

plot3(y(:,1),y(:,2),y(:,3),'r+','linewidth',1);

%stem3(y(:,1),y(:,2),y(:,3),'r+','linewidth',1); %ÈýÎ¬»ð²ñ¹÷Í¼

hold on ;

%C2=120

a=0.85, b=0.68, R1=800, W=80,C1=180,S1=100,S2=70,H1=240,H2=300,T1=300,T2=350, C2=120, C3=100,G1=180, G2=140;

[t,y]=ode45(@(t,y) Datefunction(t,y,a,b,R1,W,S1,S2,C1,C2,C3,T1,T2,G1,G2,H1,H2),[0 100],[0.2 0.2 0.2]);

plot3(y(:,1),y(:,2),y(:,3),'k--','linewidth',1);

hold on ;

%C2=140

a=0.85, b=0.68, R1=800, W=80,C1=180,S1=100,S2=70,H1=240,H2=300,T1=300,T2=350, C2=140, C3=100,G1=180, G2=140;

[t,y]=ode45(@(t,y) Datefunction(t,y,a,b,R1,W,S1,S2,C1,C2,C3,T1,T2,G1,G2,H1,H2),[0 100],[0.2 0.2 0.2]);

plot3(y(:,1),y(:,2),y(:,3),'b-','linewidth',1);

hold on ;

%×ø±ê¿Ì¶È¼ä¸ô¼°ÆäÇø¼ä£¬×ø±ê±ê×¢

set(gca,'XTick',[0:0.2:1],'YTick',[0:0.2:1],'ZTick',[0:0.2:1])

axis([0 1 0 1 0 1])

xlabel('$x$','interpreter','latex');ylabel('$y$','interpreter','latex');zlabel('$z$','interpreter','latex','Rotation',360,'position',[-0.1 1 1.1]);

%Í¼ÏóÍø¸ñ£¬µ×Í¼¼Ó°×

grid on

hold on

set(0,'defaultfigurecolor','w')

%Í¼Àý¼°±êÌâ

legend({'{\it\fontname{Bodoni MT} C_{2}}=80','{\it\fontname{Bodoni MT} C_{2}}=100','{\it\fontname{Bodoni MT} C_{2}}=120','{\it\fontname{Bodoni MT} C_{2}}=140'},'location','northeast');

%title('Fig. 8. The influence of the incentives cost C_{1}. ','FontWeight','bold','position',[0 0 -0.2]);

%¼ÓÉÏ±ê×¢ºÍ¼ýÍ·

text(0.4 ,0.2 ,0.3,'$ESS$','interpreter','latex');

annotation('arrow',[0.55 0.35],[0.35 0.32]);

annotation('arrow',[0.58 0.58],[0.38 0.45]);

% the small figure

axes('position',[0.13 0.32 0.2 0.2]); %Ð¡Í¼µÄ¹Ø¼üÓï¾äÈ·¶¨Ð¡Í¼µÄ´óÐ¡Î»ÖÃ

% z-xÐ¡Í¼ÖÐµÄÏßÌõ

%Ð¡Í¼ÖÐµÄÏßÌõ

a=0.85, b=0.68, R1=800, W=80,C1=180,S1=100,S2=70,H1=240,H2=300,T1=300,T2=350, C2=80, C3=100,G1=180, G2=140;

[t,y]=ode45(@(t,y) Datefunction(t,y,a,b,R1,W,S1,S2,C1,C2,C3,T1,T2,G1,G2,H1,H2),[0 100],[0.2 0.2 0.2]);

plot3(y(:,1),y(:,2),y(:,3),'g:.','linewidth',1);

% stem3(y(:,1),y(:,2),y(:,3),'r+','linewidth',1);%»ð²ñ¹÷Í¼

hold on

a=0.85, b=0.68, R1=800, W=80,C1=180,S1=100,S2=70,H1=240,H2=300,T1=300,T2=350, C2=100, C3=100,G1=180, G2=140;

[t,y]=ode45(@(t,y) Datefunction(t,y,a,b,R1,W,S1,S2,C1,C2,C3,T1,T2,G1,G2,H1,H2),[0 100],[0.2 0.2 0.2]);

plot3(y(:,1),y(:,2),y(:,3),'r+','linewidth',1);

% stem3(y(:,1),y(:,2),y(:,3),'g--','linewidth',1);%»ð²ñ¹÷Í¼

a=0.85, b=0.68, R1=800, W=80,C1=180,S1=100,S2=70,H1=240,H2=300,T1=300,T2=350, C2=120, C3=100,G1=180, G2=140;

[t,y]=ode45(@(t,y) Datefunction(t,y,a,b,R1,W,S1,S2,C1,C2,C3,T1,T2,G1,G2,H1,H2),[0 100],[0.2 0.2 0.2]);

plot3(y(:,1),y(:,2),y(:,3),'k--','linewidth',1);

hold on

a=0.85, b=0.68, R1=800, W=80,C1=180,S1=100,S2=70,H1=240,H2=300,T1=300,T2=350, C2=140, C3=100,G1=180, G2=140;

[t,y]=ode45(@(t,y) Datefunction(t,y,a,b,R1,W,S1,S2,C1,C2,C3,T1,T2,G1,G2,H1,H2),[0 100],[0.2 0.2 0.2]);

plot3(y(:,1),y(:,2),y(:,3),'b-','linewidth',1);

% stem3(y(:,1),y(:,2),y(:,3),'g--','linewidth',1);%»ð²ñ¹÷Í¼

hold on

%×ø±ê¿Ì¶È¼ä¸ô¼°Çø¼ä£¬µ«²»ÏÔÊ¾

set(gca,'XTick',[0:0.2:1],'YTick',[0:0.2:1],'ZTick',[0:0.2:1])

axis([0 1 0 1 0 1])

set(gca,'XTickLabel','','YTickLabel','','ZTickLabel','')

%±³¾°Íø¸ñ²¢ÉèÖÃ°×µ×

grid on

hold on

set(0,'defaultfigurecolor','w')

%Í¼ÏóÏÔÊ¾ÊÓ½Ç£¬ÒÔ¼°ÏàÓ¦µÄ×ø±ê±ê×¢

view([0 0]); %z-xÐ¡Í¼

xlabel('x','position',[0.8 1 0.3])

zlabel('z','position',[0.1 1 0.8],'Rotation',360)

1. **Fig 17. The influence of extra cost** $\boldsymbol{C}_{\mathbf{3}}$

clc,clear;

figure(17);

%C3=80

a=0.85, b=0.68, R1=800, W=80,C1=180,S1=100,S2=70,H1=240,H2=300,T1=300,T2=350, C2=120, C3=80,G1=180, G2=140;

[t,y]=ode45(@(t,y) Datefunction(t,y,a,b,R1,W,S1,S2,C1,C2,C3,T1,T2,G1,G2,H1,H2),[0 100],[0.2 0.2 0.2]);

plot3(y(:,1),y(:,2),y(:,3),'g:.','linewidth',1);

hold on ;

%C3=100

a=0.85, b=0.68, R1=800, W=80,C1=180,S1=100,S2=70,H1=240,H2=300,T1=300,T2=350, C2=120, C3=100,G1=180, G2=140;

[t,y]=ode45(@(t,y) Datefunction(t,y,a,b,R1,W,S1,S2,C1,C2,C3,T1,T2,G1,G2,H1,H2),[0 100],[0.2 0.2 0.2]);

plot3(y(:,1),y(:,2),y(:,3),'r+','linewidth',1);

%stem3(y(:,1),y(:,2),y(:,3),'r+','linewidth',1); %ÈýÎ¬»ð²ñ¹÷Í¼

hold on ;

%C3=120

a=0.85, b=0.68, R1=800, W=80,C1=180,S1=100,S2=70,H1=240,H2=300,T1=300,T2=350, C2=120, C3=120,G1=180, G2=140;

[t,y]=ode45(@(t,y) Datefunction(t,y,a,b,R1,W,S1,S2,C1,C2,C3,T1,T2,G1,G2,H1,H2),[0 100],[0.2 0.2 0.2]);

plot3(y(:,1),y(:,2),y(:,3),'k--','linewidth',1);

hold on ;

%C3=140

a=0.85, b=0.68, R1=800, W=80,C1=180,S1=100,S2=70,H1=240,H2=300,T1=300,T2=350, C2=120, C3=140,G1=180, G2=140;

[t,y]=ode45(@(t,y) Datefunction(t,y,a,b,R1,W,S1,S2,C1,C2,C3,T1,T2,G1,G2,H1,H2),[0 100],[0.2 0.2 0.2]);

plot3(y(:,1),y(:,2),y(:,3),'b-','linewidth',1);

hold on ;

%×ø±ê¿Ì¶È¼ä¸ô¼°ÆäÇø¼ä£¬×ø±ê±ê×¢

set(gca,'XTick',[0:0.2:1],'YTick',[0:0.2:1],'ZTick',[0:0.2:1])

axis([0 1 0 1 0 1])

xlabel('$x$','interpreter','latex');ylabel('$y$','interpreter','latex');zlabel('$z$','interpreter','latex','Rotation',360,'position',[-0.1 1 1.1]);

%Í¼ÏóÍø¸ñ£¬µ×Í¼¼Ó°×

grid on

hold on

set(0,'defaultfigurecolor','w')

%Í¼Àý¼°±êÌâ

legend({'{\it\fontname{Bodoni MT} C_{3}}=80','{\it\fontname{Bodoni MT} C_{3}}=100','{\it\fontname{Bodoni MT} C_{3}}=120','{\it\fontname{Bodoni MT} C_{3}}=140'},'location','northeast');

%title('Fig. 8. The influence of the incentives cost C_{1}. ','FontWeight','bold','position',[0 0 -0.2]);

%¼ÓÉÏ±ê×¢ºÍ¼ýÍ·

text(0.4 ,0.2 ,0.3,'$ESS$','interpreter','latex');

annotation('arrow',[0.55 0.35],[0.35 0.32]);

annotation('arrow',[0.58 0.58],[0.38 0.45]);

% the small figure

axes('position',[0.13 0.32 0.2 0.2]); %Ð¡Í¼µÄ¹Ø¼üÓï¾äÈ·¶¨Ð¡Í¼µÄ´óÐ¡Î»ÖÃ

% z-xÐ¡Í¼ÖÐµÄÏßÌõ

%Ð¡Í¼ÖÐµÄÏßÌõ

a=0.85, b=0.68, R1=800, W=80,C1=180,S1=100,S2=70,H1=240,H2=300,T1=300,T2=350, C2=120, C3=80,G1=180, G2=140;

[t,y]=ode45(@(t,y) Datefunction(t,y,a,b,R1,W,S1,S2,C1,C2,C3,T1,T2,G1,G2,H1,H2),[0 100],[0.2 0.2 0.2]);

plot3(y(:,1),y(:,2),y(:,3),'g:.','linewidth',1);

% stem3(y(:,1),y(:,2),y(:,3),'r+','linewidth',1);%»ð²ñ¹÷Í¼

hold on

a=0.85, b=0.68, R1=800, W=80,C1=180,S1=100,S2=70,H1=240,H2=300,T1=300,T2=350, C2=120, C3=100,G1=180, G2=140;

[t,y]=ode45(@(t,y) Datefunction(t,y,a,b,R1,W,S1,S2,C1,C2,C3,T1,T2,G1,G2,H1,H2),[0 100],[0.2 0.2 0.2]);

plot3(y(:,1),y(:,2),y(:,3),'r+','linewidth',1);

% stem3(y(:,1),y(:,2),y(:,3),'g--','linewidth',1);%»ð²ñ¹÷Í¼

a=0.85, b=0.68, R1=800, W=80,C1=180,S1=100,S2=70,H1=240,H2=300,T1=300,T2=350, C2=120, C3=120,G1=180, G2=140;

[t,y]=ode45(@(t,y) Datefunction(t,y,a,b,R1,W,S1,S2,C1,C2,C3,T1,T2,G1,G2,H1,H2),[0 100],[0.2 0.2 0.2]);

plot3(y(:,1),y(:,2),y(:,3),'k--','linewidth',1);

hold on

a=0.85, b=0.68, R1=800, W=80,C1=180,S1=100,S2=70,H1=240,H2=300,T1=300,T2=350, C2=120, C3=140,G1=180, G2=140;

[t,y]=ode45(@(t,y) Datefunction(t,y,a,b,R1,W,S1,S2,C1,C2,C3,T1,T2,G1,G2,H1,H2),[0 100],[0.2 0.2 0.2]);

plot3(y(:,1),y(:,2),y(:,3),'b-','linewidth',1);

% stem3(y(:,1),y(:,2),y(:,3),'g--','linewidth',1);%»ð²ñ¹÷Í¼

hold on

%×ø±ê¿Ì¶È¼ä¸ô¼°Çø¼ä£¬µ«²»ÏÔÊ¾

set(gca,'XTick',[0:0.2:1],'YTick',[0:0.2:1],'ZTick',[0:0.2:1])

axis([0 1 0 1 0 1])

set(gca,'XTickLabel','','YTickLabel','','ZTickLabel','')

%±³¾°Íø¸ñ²¢ÉèÖÃ°×µ×

grid on

hold on

set(0,'defaultfigurecolor','w')

%Í¼ÏóÏÔÊ¾ÊÓ½Ç£¬ÒÔ¼°ÏàÓ¦µÄ×ø±ê±ê×¢

view([0 0]); %z-xÐ¡Í¼

xlabel('x','position',[0.8 1 0.3])

zlabel('z','position',[0.1 1 0.8],'Rotation',360)

1. **Fig 18. The influence of the bonus from dishonest of the carrier** $\boldsymbol{G}_{\mathbf{1}}$

clc,clear;

figure(18);

%G1=140

a=0.85, b=0.68, R1=800, W=80,C1=180,S1=100,S2=70,H1=240,H2=300,T1=300,T2=350, C2=120, C3=100,G1=140, G2=140;

[t,y]=ode45(@(t,y) Datefunction(t,y,a,b,R1,W,S1,S2,C1,C2,C3,T1,T2,G1,G2,H1,H2),[0 100],[0.2 0.2 0.2]);

plot3(y(:,1),y(:,2),y(:,3),'g:.','linewidth',1);

hold on ;

%G1=160

a=0.85, b=0.68, R1=800, W=80,C1=180,S1=100,S2=70,H1=240,H2=300,T1=300,T2=350, C2=120, C3=100,G1=160, G2=140;

[t,y]=ode45(@(t,y) Datefunction(t,y,a,b,R1,W,S1,S2,C1,C2,C3,T1,T2,G1,G2,H1,H2),[0 100],[0.2 0.2 0.2]);

plot3(y(:,1),y(:,2),y(:,3),'r+','linewidth',1);

%stem3(y(:,1),y(:,2),y(:,3),'r+','linewidth',1); %ÈýÎ¬»ð²ñ¹÷Í¼

hold on ;

%G1=180

a=0.85, b=0.68, R1=800, W=80,C1=180,S1=100,S2=70,H1=240,H2=300,T1=300,T2=350, C2=120, C3=100,G1=180, G2=140;

[t,y]=ode45(@(t,y) Datefunction(t,y,a,b,R1,W,S1,S2,C1,C2,C3,T1,T2,G1,G2,H1,H2),[0 100],[0.2 0.2 0.2]);

plot3(y(:,1),y(:,2),y(:,3),'k--','linewidth',1);

hold on ;

%G1=200

a=0.85, b=0.68, R1=800, W=80,C1=180,S1=100,S2=70,H1=240,H2=300,T1=300,T2=350, C2=120, C3=100,G1=200, G2=140;

[t,y]=ode45(@(t,y) Datefunction(t,y,a,b,R1,W,S1,S2,C1,C2,C3,T1,T2,G1,G2,H1,H2),[0 100],[0.2 0.2 0.2]);

plot3(y(:,1),y(:,2),y(:,3),'b-','linewidth',1);

hold on ;

%×ø±ê¿Ì¶È¼ä¸ô¼°ÆäÇø¼ä£¬×ø±ê±ê×¢

set(gca,'XTick',[0:0.2:1],'YTick',[0:0.2:1],'ZTick',[0:0.2:1])

axis([0 1 0 1 0 1])

xlabel('$x$','interpreter','latex');ylabel('$y$','interpreter','latex');zlabel('$z$','interpreter','latex','Rotation',360,'position',[-0.1 1 1.1]);

%Í¼ÏóÍø¸ñ£¬µ×Í¼¼Ó°×

grid on

hold on

set(0,'defaultfigurecolor','w')

%Í¼Àý¼°±êÌâ

legend({'{\it\fontname{Bodoni MT} G_{1}}=140','{\it\fontname{Bodoni MT} G_{1}}=160','{\it\fontname{Bodoni MT} G_{1}}=180','{\it\fontname{Bodoni MT} G_{1}}=200'},'location','northeast');

%title('Fig. 8. The influence of the incentives cost C_{1}. ','FontWeight','bold','position',[0 0 -0.2]);

%¼ÓÉÏ±ê×¢ºÍ¼ýÍ·

text(0.4 ,0.2 ,0.3,'$ESS$','interpreter','latex');

annotation('arrow',[0.55 0.35],[0.35 0.32]);

annotation('arrow',[0.58 0.58],[0.38 0.45]);

% the small figure

axes('position',[0.13 0.32 0.2 0.2]); %Ð¡Í¼µÄ¹Ø¼üÓï¾äÈ·¶¨Ð¡Í¼µÄ´óÐ¡Î»ÖÃ

% z-xÐ¡Í¼ÖÐµÄÏßÌõ

%Ð¡Í¼ÖÐµÄÏßÌõ

a=0.85, b=0.68, R1=800, W=80,C1=180,S1=100,S2=70,H1=240,H2=300,T1=300,T2=350, C2=120, C3=100,G1=140, G2=140;

[t,y]=ode45(@(t,y) Datefunction(t,y,a,b,R1,W,S1,S2,C1,C2,C3,T1,T2,G1,G2,H1,H2),[0 100],[0.2 0.2 0.2]);

plot3(y(:,1),y(:,2),y(:,3),'g:.','linewidth',1);

% stem3(y(:,1),y(:,2),y(:,3),'r+','linewidth',1);%»ð²ñ¹÷Í¼

hold on

a=0.85, b=0.68, R1=800, W=80,C1=180,S1=100,S2=70,H1=240,H2=300,T1=300,T2=350, C2=120, C3=100,G1=160, G2=140;

[t,y]=ode45(@(t,y) Datefunction(t,y,a,b,R1,W,S1,S2,C1,C2,C3,T1,T2,G1,G2,H1,H2),[0 100],[0.2 0.2 0.2]);

plot3(y(:,1),y(:,2),y(:,3),'r+','linewidth',1);

% stem3(y(:,1),y(:,2),y(:,3),'g--','linewidth',1);%»ð²ñ¹÷Í¼

a=0.85, b=0.68, R1=800, W=80,C1=180,S1=100,S2=70,H1=240,H2=300,T1=300,T2=350, C2=120, C3=100,G1=180, G2=140;

[t,y]=ode45(@(t,y) Datefunction(t,y,a,b,R1,W,S1,S2,C1,C2,C3,T1,T2,G1,G2,H1,H2),[0 100],[0.2 0.2 0.2]);

plot3(y(:,1),y(:,2),y(:,3),'k--','linewidth',1);

hold on

a=0.85, b=0.68, R1=800, W=80,C1=180,S1=100,S2=70,H1=240,H2=300,T1=300,T2=350, C2=120, C3=100,G1=200, G2=140;

[t,y]=ode45(@(t,y) Datefunction(t,y,a,b,R1,W,S1,S2,C1,C2,C3,T1,T2,G1,G2,H1,H2),[0 100],[0.2 0.2 0.2]);

plot3(y(:,1),y(:,2),y(:,3),'b-','linewidth',1);

% stem3(y(:,1),y(:,2),y(:,3),'g--','linewidth',1);%»ð²ñ¹÷Í¼

hold on

%×ø±ê¿Ì¶È¼ä¸ô¼°Çø¼ä£¬µ«²»ÏÔÊ¾

set(gca,'XTick',[0:0.2:1],'YTick',[0:0.2:1],'ZTick',[0:0.2:1])

axis([0 1 0 1 0 1])

set(gca,'XTickLabel','','YTickLabel','','ZTickLabel','')

%±³¾°Íø¸ñ²¢ÉèÖÃ°×µ×

grid on

hold on

set(0,'defaultfigurecolor','w')

%Í¼ÏóÏÔÊ¾ÊÓ½Ç£¬ÒÔ¼°ÏàÓ¦µÄ×ø±ê±ê×¢

view([0 0]); %z-xÐ¡Í¼

xlabel('x','position',[0.8 1 0.3])

zlabel('z','position',[0.1 1 0.8],'Rotation',360)

1. **Fig 19. The influence of the bonus from dishonest of the shipper** $\boldsymbol{G}_{\mathbf{2}}$

clc,clear;

figure(19);

%G2=140

a=0.85, b=0.68, R1=800, W=80,C1=180,S1=100,S2=70,H1=240,H2=300,T1=300,T2=350, C2=120, C3=100,G1=180, G2=140;

[t,y]=ode45(@(t,y) Datefunction(t,y,a,b,R1,W,S1,S2,C1,C2,C3,T1,T2,G1,G2,H1,H2),[0 100],[0.2 0.2 0.2]);

plot3(y(:,1),y(:,2),y(:,3),'g:.','linewidth',1);

hold on ;

%G2=160

a=0.85, b=0.68, R1=800, W=80,C1=180,S1=100,S2=70,H1=240,H2=300,T1=300,T2=350, C2=120, C3=100,G1=180, G2=160;

[t,y]=ode45(@(t,y) Datefunction(t,y,a,b,R1,W,S1,S2,C1,C2,C3,T1,T2,G1,G2,H1,H2),[0 100],[0.2 0.2 0.2]);

plot3(y(:,1),y(:,2),y(:,3),'r+','linewidth',1);

%stem3(y(:,1),y(:,2),y(:,3),'r+','linewidth',1); %三维火柴棍图

hold on ;

%G2=180

a=0.85, b=0.68, R1=800, W=80,C1=180,S1=100,S2=70,H1=240,H2=300,T1=300,T2=350, C2=120, C3=100,G1=180, G2=180;

[t,y]=ode45(@(t,y) Datefunction(t,y,a,b,R1,W,S1,S2,C1,C2,C3,T1,T2,G1,G2,H1,H2),[0 100],[0.2 0.2 0.2]);

plot3(y(:,1),y(:,2),y(:,3),'k--','linewidth',1);

hold on ;

%G2=200

a=0.85, b=0.68, R1=800, W=80,C1=180,S1=100,S2=70,H1=240,H2=300,T1=300,T2=350, C2=120, C3=100,G1=180, G2=200;

[t,y]=ode45(@(t,y) Datefunction(t,y,a,b,R1,W,S1,S2,C1,C2,C3,T1,T2,G1,G2,H1,H2),[0 100],[0.2 0.2 0.2]);

plot3(y(:,1),y(:,2),y(:,3),'b-','linewidth',1);

hold on ;

%坐标刻度间隔及其区间，坐标标注

set(gca,'XTick',[0:0.2:1],'YTick',[0:0.2:1],'ZTick',[0:0.2:1])

axis([0 1 0 1 0 1])

xlabel('$x$','interpreter','latex');ylabel('$y$','interpreter','latex');zlabel('$z$','interpreter','latex','Rotation',360,'position',[-0.1 1 1.1]);

%图象网格，底图加白

grid on

hold on

set(0,'defaultfigurecolor','w')

%图例及标题

legend({'{\it\fontname{Bodoni MT} G_{2}}=140','{\it\fontname{Bodoni MT} G_{2}}=160','{\it\fontname{Bodoni MT} G_{2}}=180','{\it\fontname{Bodoni MT} G_{2}}=200'},'location','northeast');

%title('Fig. 8. The influence of the incentives cost C_{1}. ','FontWeight','bold','position',[0 0 -0.2]);

%加上标注和箭头

text(0.4 ,0.2 ,0.3,'$ESS$','interpreter','latex');

annotation('arrow',[0.55 0.35],[0.35 0.32]);

annotation('arrow',[0.58 0.58],[0.38 0.45]);

% the small figure

axes('position',[0.13 0.32 0.2 0.2]); %小图的关键语句确定小图的大小位置

% z-x小图中的线条

%小图中的线条

a=0.85, b=0.68, R1=800, W=80,C1=180,S1=100,S2=70,H1=240,H2=300,T1=300,T2=350, C2=120, C3=100,G1=180, G2=140;

[t,y]=ode45(@(t,y) Datefunction(t,y,a,b,R1,W,S1,S2,C1,C2,C3,T1,T2,G1,G2,H1,H2),[0 100],[0.2 0.2 0.2]);

plot3(y(:,1),y(:,2),y(:,3),'g:.','linewidth',1);

% stem3(y(:,1),y(:,2),y(:,3),'r+','linewidth',1);%火柴棍图

hold on

a=0.85, b=0.68, R1=800, W=80,C1=180,S1=100,S2=70,H1=240,H2=300,T1=300,T2=350, C2=120, C3=100,G1=180, G2=160;

[t,y]=ode45(@(t,y) Datefunction(t,y,a,b,R1,W,S1,S2,C1,C2,C3,T1,T2,G1,G2,H1,H2),[0 100],[0.2 0.2 0.2]);

plot3(y(:,1),y(:,2),y(:,3),'r+','linewidth',1);

% stem3(y(:,1),y(:,2),y(:,3),'g--','linewidth',1);%火柴棍图

a=0.85, b=0.68, R1=800, W=80,C1=180,S1=100,S2=70,H1=240,H2=300,T1=300,T2=350, C2=120, C3=100,G1=180, G2=180;

[t,y]=ode45(@(t,y) Datefunction(t,y,a,b,R1,W,S1,S2,C1,C2,C3,T1,T2,G1,G2,H1,H2),[0 100],[0.2 0.2 0.2]);

plot3(y(:,1),y(:,2),y(:,3),'k--','linewidth',1);

hold on

a=0.85, b=0.68, R1=800, W=80,C1=180,S1=100,S2=70,H1=240,H2=300,T1=300,T2=350, C2=120, C3=100,G1=180, G2=200;

[t,y]=ode45(@(t,y) Datefunction(t,y,a,b,R1,W,S1,S2,C1,C2,C3,T1,T2,G1,G2,H1,H2),[0 100],[0.2 0.2 0.2]);

plot3(y(:,1),y(:,2),y(:,3),'b-','linewidth',1);

% stem3(y(:,1),y(:,2),y(:,3),'g--','linewidth',1);%火柴棍图

hold on

%坐标刻度间隔及区间，但不显示

set(gca,'XTick',[0:0.2:1],'YTick',[0:0.2:1],'ZTick',[0:0.2:1])

axis([0 1 0 1 0 1])

set(gca,'XTickLabel','','YTickLabel','','ZTickLabel','')

%背景网格并设置白底

grid on

hold on

set(0,'defaultfigurecolor','w')

%图象显示视角，以及相应的坐标标注

view([0 0]); %z-x小图

xlabel('x','position',[0.8 1 0.3])

zlabel('z','position',[0.1 1 0.8],'Rotation',360)

1. **Fig 20. The influence of loss preference coefficient** $\boldsymbol{L}_{\mathbf{P}}$

clc,clear;

figure(20);

%LP=1.2

a=0.85, b=0.68, R1=800, W=80,C1=180,S1=100,S2=70,H1=240,H2=300,T1=300,T2=350, C2=120, C3=100,G1=180, G2=140, LP=1.2,LS=1.6;

[t,y]=ode45(@(t,y) Datefunction(t,y,a,b,R1,W,S1,S2,C1,C2,C3,T1,T2,G1,G2,H1,H2,LP,LS),[0 100],[0.2 0.2 0.2]);

plot3(y(:,1),y(:,2),y(:,3),'g:.','linewidth',1);

hold on ;

%LP=1.2

a=0.85, b=0.68, R1=800, W=80,C1=180,S1=100,S2=70,H1=240,H2=300,T1=300,T2=350, C2=120, C3=100,G1=180, G2=140, LP=1.7,LS=1.6;

[t,y]=ode45(@(t,y) Datefunction(t,y,a,b,R1,W,S1,S2,C1,C2,C3,T1,T2,G1,G2,H1,H2,LP,LS),[0 100],[0.2 0.2 0.2]);

plot3(y(:,1),y(:,2),y(:,3),'r+','linewidth',1);

%stem3(y(:,1),y(:,2),y(:,3),'r+','linewidth',1); %ÈýÎ¬»ð²ñ¹÷Í¼

hold on ;

%LP=1.3

a=0.85, b=0.68, R1=800, W=80,C1=180,S1=100,S2=70,H1=240,H2=300,T1=300,T2=350, C2=120, C3=100,G1=180, G2=140, LP=2.2,LS=1.6;

[t,y]=ode45(@(t,y) Datefunction(t,y,a,b,R1,W,S1,S2,C1,C2,C3,T1,T2,G1,G2,H1,H2,LP,LS),[0 100],[0.2 0.2 0.2]);

plot3(y(:,1),y(:,2),y(:,3),'k--','linewidth',1);

hold on ;

%LP=1.4

a=0.85, b=0.68, R1=800, W=80,C1=180,S1=100,S2=70,H1=240,H2=300,T1=300,T2=350, C2=120, C3=100,G1=180, G2=140, LP=2.5,LS=1.6;

[t,y]=ode45(@(t,y) Datefunction(t,y,a,b,R1,W,S1,S2,C1,C2,C3,T1,T2,G1,G2,H1,H2,LP,LS),[0 100],[0.2 0.2 0.2]);

plot3(y(:,1),y(:,2),y(:,3),'b-','linewidth',1);

hold on ;

%×ø±ê¿Ì¶È¼ä¸ô¼°ÆäÇø¼ä£¬×ø±ê±ê×¢

set(gca,'XTick',[0:0.2:1],'YTick',[0:0.2:1],'ZTick',[0:0.2:1])

axis([0 1 0 1 0 1])

xlabel('$x$','interpreter','latex');ylabel('$y$','interpreter','latex');zlabel('$z$','interpreter','latex','Rotation',360,'position',[-0.1 1 1.1]);

%Í¼ÏóÍø¸ñ£¬µ×Í¼¼Ó°×

grid on

hold on

set(0,'defaultfigurecolor','w')

%Í¼Àý¼°±êÌâ

legend({'{\it\fontname{Bodoni MT}L_{P}}=1.2','{\it\fontname{Bodoni MT}L_{P}}=1.7','{\it\fontname{Bodoni MT}L_{P}}=2.2','{\it\fontname{Bodoni MT}L_{P}}=2.7'},'location','northeast');

%title('Fig. 8. The influence of the incentives cost C_{1}. ','FontWeight','bold','position',[0 0 -0.2]);

%¼ÓÉÏ±ê×¢ºÍ¼ýÍ·

text(0.4 ,0.2 ,0.3,'$ESS$','interpreter','latex');

annotation('arrow',[0.55 0.35],[0.35 0.32]);

annotation('arrow',[0.58 0.58],[0.38 0.45]);

% the small figure

axes('position',[0.13 0.32 0.2 0.2]); %Ð¡Í¼µÄ¹Ø¼üÓï¾äÈ·¶¨Ð¡Í¼µÄ´óÐ¡Î»ÖÃ

% z-xÐ¡Í¼ÖÐµÄÏßÌõ

%Ð¡Í¼ÖÐµÄÏßÌõ

a=0.85, b=0.68, R1=800, W=80,C1=180,S1=100,S2=70,H1=240,H2=300,T1=300,T2=350, C2=120, C3=100,G1=180, G2=140, LP=1.2,LS=1.6;

[t,y]=ode45(@(t,y) Datefunction(t,y,a,b,R1,W,S1,S2,C1,C2,C3,T1,T2,G1,G2,H1,H2,LP,LS),[0 100],[0.2 0.2 0.2]);

plot3(y(:,1),y(:,2),y(:,3),'g:.','linewidth',1);

% stem3(y(:,1),y(:,2),y(:,3),'r+','linewidth',1);%»ð²ñ¹÷Í¼

hold on

a=0.85, b=0.68, R1=800, W=80,C1=180,S1=100,S2=70,H1=240,H2=300,T1=300,T2=350, C2=120, C3=100,G1=180, G2=140, LP=1.7,LS=1.6;

[t,y]=ode45(@(t,y) Datefunction(t,y,a,b,R1,W,S1,S2,C1,C2,C3,T1,T2,G1,G2,H1,H2,LP,LS),[0 100],[0.2 0.2 0.2]);

plot3(y(:,1),y(:,2),y(:,3),'r+','linewidth',1);

% stem3(y(:,1),y(:,2),y(:,3),'g--','linewidth',1);%»ð²ñ¹÷Í¼

a=0.85, b=0.68, R1=800, W=80,C1=180,S1=100,S2=70,H1=240,H2=300,T1=300,T2=350, C2=120, C3=100,G1=180, G2=140, LP=2.2,LS=1.6;

[t,y]=ode45(@(t,y) Datefunction(t,y,a,b,R1,W,S1,S2,C1,C2,C3,T1,T2,G1,G2,H1,H2,LP,LS),[0 100],[0.2 0.2 0.2]);

plot3(y(:,1),y(:,2),y(:,3),'k--','linewidth',1);

hold on

a=0.85, b=0.68, R1=800, W=80,C1=180,S1=100,S2=70,H1=240,H2=300,T1=300,T2=350, C2=120, C3=100,G1=180, G2=140, LP=2.7,LS=1.6;

[t,y]=ode45(@(t,y) Datefunction(t,y,a,b,R1,W,S1,S2,C1,C2,C3,T1,T2,G1,G2,H1,H2,LP,LS),[0 100],[0.2 0.2 0.2]);

plot3(y(:,1),y(:,2),y(:,3),'b-','linewidth',1);

% stem3(y(:,1),y(:,2),y(:,3),'g--','linewidth',1);%»ð²ñ¹÷Í¼

hold on

%×ø±ê¿Ì¶È¼ä¸ô¼°Çø¼ä£¬µ«²»ÏÔÊ¾

set(gca,'XTick',[0:0.2:1],'YTick',[0:0.2:1],'ZTick',[0:0.2:1])

axis([0 1 0 1 0 1])

set(gca,'XTickLabel','','YTickLabel','','ZTickLabel','')

%±³¾°Íø¸ñ²¢ÉèÖÃ°×µ×

grid on

hold on

set(0,'defaultfigurecolor','w')

%Í¼ÏóÏÔÊ¾ÊÓ½Ç£¬ÒÔ¼°ÏàÓ¦µÄ×ø±ê±ê×¢

view([0 0]); %z-xÐ¡Í¼

xlabel('x','position',[0.8 1 0.3])

zlabel('z','position',[0.1 1 0.8],'Rotation',360)

1. **Fig 21. The influence of loss preference coefficient** $\boldsymbol{L}_{\mathbf{S}}$

clc,clear;

figure(21);

%LS=1.2

a=0.85, b=0.68, R1=800, W=80,C1=180,S1=100,S2=70,H1=240,H2=300,T1=300,T2=350, C2=120, C3=100,G1=180, G2=140, LP=1.2,LS=1.2;

[t,y]=ode45(@(t,y) Datefunction(t,y,a,b,R1,W,S1,S2,C1,C2,C3,T1,T2,G1,G2,H1,H2,LP,LS),[0 100],[0.2 0.2 0.2]);

plot3(y(:,1),y(:,2),y(:,3),'g:.','linewidth',1);

hold on ;

%LS=1.7

a=0.85, b=0.68, R1=800, W=80,C1=180,S1=100,S2=70,H1=240,H2=300,T1=300,T2=350, C2=120, C3=100,G1=180, G2=140, LP=1.2,LS=1.7;

[t,y]=ode45(@(t,y) Datefunction(t,y,a,b,R1,W,S1,S2,C1,C2,C3,T1,T2,G1,G2,H1,H2,LP,LS),[0 100],[0.2 0.2 0.2]);

plot3(y(:,1),y(:,2),y(:,3),'r+','linewidth',1);

%stem3(y(:,1),y(:,2),y(:,3),'r+','linewidth',1); %三维火柴棍图

hold on ;

%LS=2.2

a=0.85, b=0.68, R1=800, W=80,C1=180,S1=100,S2=70,H1=240,H2=300,T1=300,T2=350, C2=120, C3=100,G1=180, G2=140, LP=1.2,LS=2.2;

[t,y]=ode45(@(t,y) Datefunction(t,y,a,b,R1,W,S1,S2,C1,C2,C3,T1,T2,G1,G2,H1,H2,LP,LS),[0 100],[0.2 0.2 0.2]);

plot3(y(:,1),y(:,2),y(:,3),'k--','linewidth',1);

hold on ;

%LS=2.7

a=0.85, b=0.68, R1=800, W=80,C1=180,S1=100,S2=70,H1=240,H2=300,T1=300,T2=350, C2=120, C3=100,G1=180, G2=140, LP=1.2,LS=2.7;

[t,y]=ode45(@(t,y) Datefunction(t,y,a,b,R1,W,S1,S2,C1,C2,C3,T1,T2,G1,G2,H1,H2,LP,LS),[0 100],[0.2 0.2 0.2]);

plot3(y(:,1),y(:,2),y(:,3),'b-','linewidth',1);

hold on ;

%坐标刻度间隔及其区间，坐标标注

set(gca,'XTick',[0:0.2:1],'YTick',[0:0.2:1],'ZTick',[0:0.2:1])

axis([0 1 0 1 0 1])

xlabel('$x$','interpreter','latex');ylabel('$y$','interpreter','latex');zlabel('$z$','interpreter','latex','Rotation',360,'position',[-0.1 1 1.1]);

%图象网格，底图加白

grid on

hold on

set(0,'defaultfigurecolor','w')

%图例及标题

legend({'{\it\fontname{Bodoni MT}L_{S}}=1.2','{\it\fontname{Bodoni MT}L_{S}}=1.7','{\it\fontname{Bodoni MT}L_{S}}=2.2','{\it\fontname{Bodoni MT}L_{S}}=2.7'},'location','northeast');

%title('Fig. 8. The influence of the incentives cost C_{1}. ','FontWeight','bold','position',[0 0 -0.2]);

%加上标注和箭头

text(0.4 ,0.2 ,0.3,'$ESS$','interpreter','latex');

annotation('arrow',[0.55 0.35],[0.35 0.32]);

annotation('arrow',[0.58 0.58],[0.38 0.45]);

% the small figure

axes('position',[0.13 0.32 0.2 0.2]); %小图的关键语句确定小图的大小位置

% z-x小图中的线条

%小图中的线条

a=0.85, b=0.68, R1=800, W=80,C1=180,S1=100,S2=70,H1=240,H2=300,T1=300,T2=350, C2=120, C3=100,G1=180, G2=140, LP=1.2,LS=1.2;

[t,y]=ode45(@(t,y) Datefunction(t,y,a,b,R1,W,S1,S2,C1,C2,C3,T1,T2,G1,G2,H1,H2,LP,LS),[0 100],[0.2 0.2 0.2]);

plot3(y(:,1),y(:,2),y(:,3),'g:.','linewidth',1);

% stem3(y(:,1),y(:,2),y(:,3),'r+','linewidth',1);%火柴棍图

hold on

a=0.85, b=0.68, R1=800, W=80,C1=180,S1=100,S2=70,H1=240,H2=300,T1=300,T2=350, C2=120, C3=100,G1=180, G2=140, LP=1.2,LS=1.7;

[t,y]=ode45(@(t,y) Datefunction(t,y,a,b,R1,W,S1,S2,C1,C2,C3,T1,T2,G1,G2,H1,H2,LP,LS),[0 100],[0.2 0.2 0.2]);

plot3(y(:,1),y(:,2),y(:,3),'r+','linewidth',1);

% stem3(y(:,1),y(:,2),y(:,3),'g--','linewidth',1);%火柴棍图

a=0.85, b=0.68, R1=800, W=80,C1=180,S1=100,S2=70,H1=240,H2=300,T1=300,T2=350, C2=120, C3=100,G1=180, G2=140, LP=1.2,LS=2.2;

[t,y]=ode45(@(t,y) Datefunction(t,y,a,b,R1,W,S1,S2,C1,C2,C3,T1,T2,G1,G2,H1,H2,LP,LS),[0 100],[0.2 0.2 0.2]);

plot3(y(:,1),y(:,2),y(:,3),'k--','linewidth',1);

hold on

a=0.85, b=0.68, R1=800, W=80,C1=180,S1=100,S2=70,H1=240,H2=300,T1=300,T2=350, C2=120, C3=100,G1=180, G2=140, LP=1.2,LS=2.7;

[t,y]=ode45(@(t,y) Datefunction(t,y,a,b,R1,W,S1,S2,C1,C2,C3,T1,T2,G1,G2,H1,H2,LP,LS),[0 100],[0.2 0.2 0.2]);

plot3(y(:,1),y(:,2),y(:,3),'b-','linewidth',1);

% stem3(y(:,1),y(:,2),y(:,3),'g--','linewidth',1);%火柴棍图

hold on

%坐标刻度间隔及区间，但不显示

set(gca,'XTick',[0:0.2:1],'YTick',[0:0.2:1],'ZTick',[0:0.2:1])

axis([0 1 0 1 0 1])

set(gca,'XTickLabel','','YTickLabel','','ZTickLabel','')

%背景网格并设置白底

grid on

hold on

set(0,'defaultfigurecolor','w')

%图象显示视角，以及相应的坐标标注

view([0 0]); %z-x小图

xlabel('x','position',[0.8 1 0.3])

zlabel('z','position',[0.1 1 0.8],'Rotation',360)
