# Supplementary material for: Data-driven mechanisms for network freight platforms: An evolutionary game perspective
Source: PLoS One. 2025 Jun 27;20(6):e0319842. doi: 10.1371/journal.pone.0319842 (PMC12204633; doi:10.1371/journal.pone.0319842)
Supplement: S1 File — (ZIP) [file pone.0319842.s001.zip › Programs/Replication dynamic equations.docx]

%%%%%%% **Replication dynamic equations**

function dydt=Datefunction(t,y,a,b,R1,W,S1,S2,C1,C2,C3,T1,T2,G1,G2,H1,H2,LP,LS)

dydt=zeros(3,1);

dydt(1)=-y(1)*(y(1)-1)*(R1-LP*C1 + W - LP*H1*a - LP*H2*a + LP*H1*b + LP*H2*b + LP*S1*a + LP*S2*a - LP*S1*b + T1*a - LP*S2*b + T2*a - T1*b - T2*b + LP*H1*a*y(2) - LP*H1*b*y(2) + LP*H2*a*y(3) - LP*H2*b*y(3) - LP*S1*a*y(2) + LP*S1*b*y(2) - T1*a*y(2) - LP*S2*a*y(3) + T1*b*y(2) + LP*S2*b*y(3) - T2*a*y(3) + T2*b*y(3));

dydt(2)=y(2)*(y(2)-1)*(C2 + G1 - S1*b - T1*b - S1*y(1) + S1*b*y(1) - T1*a*y(1) + T1*b*y(1) + S1*y(1)*y(3) - S1*a*y(1)*y(3));

dydt(3)=y(3)*(y(3)-1)*(LS*C3 + G2 - S2 - T2*LS*b + S2*y(2) - LS*T2*a*y(1)- S2*b*y(2) + LS*T2*b*y(1) - S2*a*y(1)*y(2) + S2*b*y(1)*y(2));

end
